# Transient callosal projections of L4 neurons are eliminated for the acquisition of local connectivity

**DOI:** 10.1038/s41467-019-12495-w

**Published:** 2019-10-07

**Authors:** N. S. De León Reyes, S. Mederos, I. Varela, L. A. Weiss, G. Perea, M. J. Galazo, M. Nieto

**Affiliations:** 1 Department of Cellular and Molecular Biology, Centro Nacional de Biotecnología, Consejo Superior de Investigaciones Científicas (CNB-CSIC), Campus de Cantoblanco, Darwin 3, 28049 Madrid, Spain; 2 Instituto Cajal, CSIC. Av. Doctor Arce, 37, 28002 Madrid, Spain; 3 Department of Cell and Molecular Biology and Tulane Brain Institute, Tulane University, 6400 Freret Street, Percival Stern Hall suite 2000, New Orleans, LA 70118 USA

**Keywords:** Neuroscience, Development of the nervous system, Axon and dendritic guidance, Cell fate and cell lineage, Cell type diversity

## Abstract

Interhemispheric axons of the corpus callosum (CC) facilitate the higher order functions of the cerebral cortex. According to current views, callosal and non-callosal fates are determined early after a neuron’s birth, and certain populations, such as cortical layer (L) 4 excitatory neurons of the primary somatosensory (S1) barrel, project only ipsilaterally. Using a novel axonal-retrotracing strategy and GFP-targeted visualization of Rorb^+^ neurons, we instead demonstrate that L4 neurons develop transient interhemispheric axons. Locally restricted L4 connectivity emerges when exuberant contralateral axons are refined in an area- and layer-specific manner during postnatal development. Surgical and genetic interventions of sensory circuits demonstrate that refinement rates depend on distinct inputs from sensory-specific thalamic nuclei. Reductions in input-dependent refinement result in mature functional interhemispheric hyperconnectivity, demonstrating the plasticity and bona fide callosal potential of L4 neurons. Thus, L4 neurons discard alternative interhemispheric circuits as instructed by thalamic input. This may ensure optimal wiring.

## Abstract (web summary)

It is traditionally believed that callosal and non-callosal fates are determined early after a neuron’s birth, and that cortical layer (L) 4 excitatory neurons of the primary somatosensory (S1) barrel cortex project only ipsilaterally. However, here authors demonstrate, using a novel axonal retrotracing strategy, that L4 neurons develop transient interhemispheric axons that are refined in an area- and layer-specific manner during postnatal development.

## Introduction

Neurons of the cerebral cortex establish complex circuits that mediate the high cognitive functions and social behaviors of the human brain^1^. In order to wire selectively and stereotypically during development, cortical neurons are thought to be intrinsically instructed with wiring rules that limit their connectivity^2^. However, cortical neurons also display remarkable plasticity that enables optimization of functional circuits and alternative wiring in non-canonical scenarios, such as in the case of loss of sensory inputs or brain insults in young brains^3–6^. This latter scenario requires considerable structural re-arrangement of axons. How these alternative circuits emerge is not well understood, nor is it understood why early postmitotic neurons exhibit greater plasticity upon molecular reprogramming^7^. We investigated these questions in the context of the selection of the neuronal populations that form the corpus callosum (CC). By understanding how callosal connections are selected we can identify rules of brain wiring that will allow us to better comprehend the altered circuits that emerge when developmental mechanisms fail, such as in cases of mental retardation, bipolar disorders, schizophrenia, autism spectrum disorders, or epilepsies^8,9^.

The CC, the largest axonal tract in mammals, connects the two halves of the cerebral cortex and functions as an information highway that facilitates the higher-order functions of the human brain^9,10^. The cerebral cortex is organized into six layers (L1–6), each roughly aggregating neurons with common molecular identities and patterns of connectivity^11^. In adults, only a subset of cortical neurons has callosal connections^10,12^. Classical studies in mice, cats, and primates, in which axonal retrotracers were injected into the cortical plate of the opposite (contralateral) hemisphere of adult animals, found that the distribution of callosally projecting neurons (CPNs) varies within the functional areas, and that in most areas, CPNs are predominantly located in L2/3 and L5, and some in L6. Very few callosal neurons are located in L4^10,13–17^. When similar retrotracing injections were performed in developing animals, many more CPNs were found in young animals than in adult, revealing the crucial role of axonal elimination in the process of callosal selection^13–20^. These injections also showed that refinement of callosal projections did not involve neuronal cell death, in contrast to what had been observed in the retina^14^. These foundational studies in the CC, together with those in the retina^21^ and with investigations describing cortical exuberant projections to subcortical targets^20,22^, identified axonal refinement as being a principal mechanism during the wiring of the central nervous system. Importantly, these experiments also determined that the increased number of CPN of developing brains were located preferentially in those layers that contain CPN in adults. Certain layers, such as L4, that contain few callosals in the adult, also showed sparse retrotracer labeling in the embryo and young postnatal individuals^13,14,16,17,23,24^. Studies interrogating how these restricted CPN distributions arise and how cortical callosal and non-callosal populations emerge concluded that restrictions to neuronal connectivity are established prior to axonal extension^16,20,25^. This led to the currently held assumption that there is an early preselection of the callosal and non-callosal subpopulations and that only axons of certain populations develop to cross the midline^10,12^. This model of wiring necessarily restricts the diversity of possible interhemispheric maps.

Subsequent studies supported the idea that neuronal fate determinants, particularly certain transcription factors, are responsible for the early sorting of cortical neuronal types^2,26^. More recent characterizations of the molecular profiles of callosal neurons reveal a complex scenario in which neurons exhibit a higher degree of heterogeneity than would be predicted by their shared projection patterns^24,27^. Concurrently, several studies have shown that the initial molecular profiles of postmitotic neurons are progressively modified, suggesting that neurons incorporate additional wiring instructions during their postnatal life^11,28–32^. A dynamic acquisition of neuronal fates provides a more comprehensive account of how cortical neurons are specified and helps explain their early plasticity, but poses unanswered questions about how and when cortical neurons commit their dendritic and axonal structures during the process of fate determination.

Herein, we investigated the numbers and exact laminar and area locations of exuberant CPN during development using injections of retrograde tracers to label callosal axons passing through the contralateral white matter (WM). By combining this retrograde analysis with genetic labeling of Rorb^+^ neurons, we demonstrate that L4 neurons, which in the adult are mostly restricted to local ipsilateral circuits, develop transient callosal axons during their normal differentiation. Mature non-callosal local connectivity is subsequently achieved through extensive postnatal refinement, which we showed depends on area- and order-specific thalamic nuclei inputs. Further, we demonstrated that the decision to eliminate an exuberant callosal axon is plastic and thus that early L4 postmitotic neurons have the potential for multiple wiring fates (ipsilateral-only versus interhemispheric)^29,33,34^.

## Results

### Neurons from all layers extend transient callosal axons

**Fig. 1:**
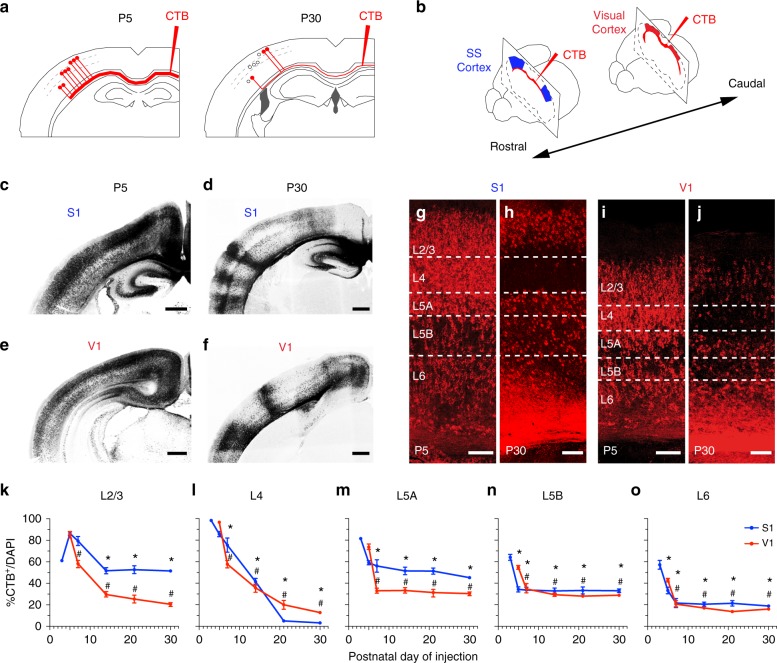
Retrotracer injections in the CC reveal transient exuberant interhemispheric projections. **a**, **b** Cartoons representing neuronal labeling upon injection of CTB in the contralateral CC (**a**) and positions of injections targeting the somatosensory (S) cortex and visual (V) cortex (**b**). **c**–**f** Coronal sections of brains injected at P5 (**c**, **e**) or P30 (**d**, **f**) in primary sensory cortices (CTB-555 shown in dark). Minor anterograde transport highlights axonal columns in P30 brains. **g**–**j** Magnifications of S1 and V1 from **c** to **f**, respectively, showing cells that were labeled upon CTB injections at P5 or P30. (Cortical layers are delineated.) **k**–**o** Quantification of the proportion of callosally projecting (CTB^+^) neurons out of total DAPI^+^ nuclei in each layer. CTB injections were made at P3, P5, P7, P14, P21, and P30. Mean ± SEM (error bars) (*n* = 300 cells; *n* = 2 sections per animal; P3, P7, P14, P21, *n* = 3 animals, and P5 and P30, *n* = 6 animals). *P* values_CTB/time_ ≤0.0001; *p* values_S1/V1_ ≤0.0001 (**k**–**o**) (two-way ANOVA (*n* = 27 mice): *F*_CTB/time_ (4, 63) = 144.0 and *F*_S1/V1_ (1, 63) = 10.16 (**k**); *F*_CTB/time_ (4, 63) = 336.9 and *F*_S1/V1_ (1, 63) = 11.14 (**l**); *F*_CTB/time_ (4, 63) = 31.87, *F*_S1/V1_ (1, 63) = 13 (**m**); *F*_CTB/time_ (4, 63) = 12.87 and *F*_S1/V1_ (1, 63) = 10.56 (**n**); *F*_CTB/time_ (4, 63) = 30.35 and *F*_S1/V1_ (1, 63) = 3.773 (**o**)). *P* value_CTB+ in S1 vs. CTB+ at P5 in S1_ *≤0.0001 (**k**), *p* value_CTB+ in S1 vs. CTB+ at P3 in S1_ *≤0.0001 (**l–o**), *p* value_CTB+ V1 vs. CTB+ V1 at P5_^#^≤0.0001 (**k–o**) (post hoc with Tukey’s test). Scale bars: 500 µm (**c**–**f**) and 100 µm (**g**–**j**). Source data are provided as a Source Data file

We set out to investigate the selection of CPN during development using a novel strategy whereby retrograde tracers (fluorescently labeled cholera toxin B (CTB)) were injected directly into the CC of one hemisphere (Fig. 1a, b, Supplementary Methods, and Supplementary Fig. 1a–h) unlike classical experiments in which CTB was injected in the cortical plate (Supplementary Fig. 1i–l)^14–17,24^. Compared to the traditional injections of CTB in the cortical plate, this procedure increases the efficiency of CPN labeling by ensuring contact with most commissural axons independent of their axonal behavior in the cortical plate. It labels both CPN with many branches in the cortical plate and those with few or no branches. Control experiments supported specificity, saturation, and efficacy of the labeling in these CC injections (see Methods and Supplementary Fig. 1 and 2). CTB injections in the CC of postnatal day (P) 30 animals and analysis of the opposite hemisphere 48 h later revealed broad labeling of different cortical areas (Supplementary Fig. 1a, b). It revealed area- and layer-specific CPN distributions (Fig. 1d, f, h, j) in agreement with previous reports^10^, including very few CPN in L4 (Fig. 1h, j). This result thus supported the use of this methodological strategy for the identification of mature callosal projections. Surprisingly, when we injected P5 animals with CTB in the CC, we observed labeling of the vast majority of cortical neurons along the medial to lateral axis, and across all layers (Fig. 1c, e, g, i). Most notably, these injections identify numerous exuberant developmental projections in L4 that have not been previously reported. We then investigated CPN developmental dynamics by performing CTB injections at representative postnatal stages and at different rostro-caudal positions (Fig. 1b), thereby labeling several areas, including primary somatosensory (S1) and visual (V1) cortices (Fig. 1c–j, Supplementary Fig. 1c, d). After injections, the number of CTB^+^ cells was quantified as a percentage of total cell nuclei at each stage (Fig. 1k–o)^35^. Quantifications were made 2 or more days after the injections, and always in animals no younger than P10. This ensured that neuronal migration had ended as well as that the initial waves of glial growth and cell death in the cortical plate had concluded (see Methods). This analysis showed that the proportions of labeled CPN are highest at early stages and gradually decreases during postnatal development, following distinct kinetics in each layer and area until the characteristic distribution of the adult is achieved (Fig. 1k–o).

**Fig. 2:**
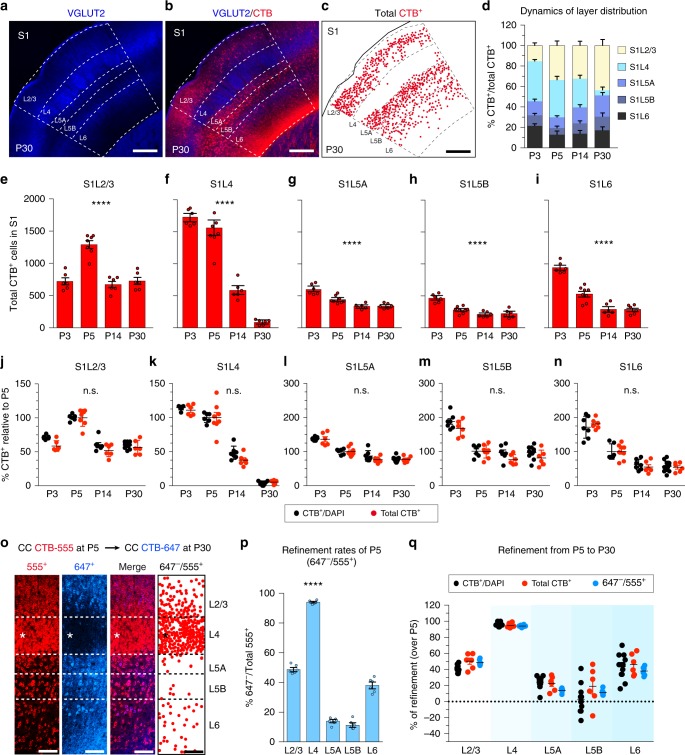
Three quantifications methods provide indistinguishable refinement rates. **a**–**c** S1 area in an injected P30 mouse. Dots in **c** indicate CTB^+^ cells. **d** Layer distribution of CTB^+^ cells within S1 after injections at indicated stages (*n* = 21 mice). **e**–**i** Total number of contralateral S1 CTB^+^ cells after injections at indicated stages (*n* = 21 mice; *n* = 3 for P3, *n* = 6 for P5, *n* = 6 for P14, *n* = 6 for P30). *P* values_CTB/time_ ****≤0.0001 (two-way ANOVA, *n* = 25 mice: *F*_CTB/time_ (3, 22) = 29.94 (**e**); *F*_CTB/time_ (3, 22) = 93.57 (**f**); *F*_CTB/time_ (3, 22) = 28.11 (**g**); *F*_CTB/time_ (3, 22) = 25.69 (**h**); *F*_CTB/time_ (3, 22) = 63.20 (**i**)). **j**–**n** Comparison of refinement rates (normalized to the respective mean values in P5) (*n* = 25 mice total CTB^+^; *n* = 18 mice CTB^+^ over DAPI). (n.s.) *P* value_CTB+ over DAPI/ total CTB+_ = nonsignificant (two-way ANOVA (*n* = 25): *F*_CTB+ over DAPI/ total CTB+_ (3, 49) = 1.504 (**j**); *F*_CTB+ over DAPI/ total CTB_+ (3, 50) = 1.130 (**k**); *F*_CTB+ over DAPI/ total CTB+_ (3, 50) = 0.758 (**l**); *F*_CTB+ over DAPI/ total CTB+_ (3, 50) = 0.732 (**m**); *F*_CTB+ over DAPI/ total CTB_+ (3, 50) = 0.422 (**n**)). **o** P32 S1 cortex injected sequentially at P5 (CTB-555) and at P30 (CTB-647). Dots represent cells labeled at P5 but not at P30. **p** Quantifications showing the fractions of CTB-555+, CTB-647^−^ as in **o** (*n* = 1000 neurons per layer, *n* = 3 mice). *P* value_refinement/layer_ ≤0.0001 (one-way ANOVA (*n* = 3): *F*_refinement/layer_ (4, 25) = 499.6). ****P value ≤0.0001 compared to any layer (Mann–Whitney test). **q** Comparison of refinement rates of CPN labeled at P5 and P30. Values are expressed as % of refined P5 CTB^+^ cells (*n* = 15 mice total; *n* = 3 double injected, *n* = 6 CTB+ over DAPI, and *n* = 6 total numbers of CTB^+^). *P* value_refinement rates/quantification method_ = nonsignificant (two-way ANOVA (*n* = 15): *F*_refinement rates/quantification method_ (8, 105) = 1.80). Data show mean ± SEM (error bars) (**d**–**q**). Scale bars: 200 µm in **a** and 100 µm in **o**. Source data are provided as a Source Data file

To evaluate if postnatal proliferation of non-neuronal cells could alter the observed refinement rates, we counted the absolute number of CTB^+^ neurons in the S1 region as delimited by the anatomical landmarks of this area (i.e., the barrels, Fig. 2a–i). Both the contributions of each layer to interhemispheric connectivity (Fig. 2d) and the variation in the total number of CPN in each layer (Fig. 2e–i) reflected a significant decrease in callosal projections during development. Importantly, when comparing these two quantification procedures by normalizing to the respective P5 mean values, refinement rates were indistinguishable (Fig. 2j–n).

We also assessed the possible contributions of cell death to refinement rates. To do so, we performed dual sequential injections of CTB coupled to distinct fluorescent probes in the same animal at P5 and P30. In these S1 double-injected animals, we counted the fraction of cells labeled with the first fluorophore and not with the second, thus reflecting the elimination of neurons that had an early callosal projection and survive in the mature circuit (Fig. 2o, p, Supplementary Fig. 2c–f). As with the single injections, the highest refinement rates were observed in S1L4. Further, these double injections indicated that most neurons that comprise L4 in P30 animals have eliminated a P5 callosal projection (Fig. 2o, p).

All three quantification methods showed equivalent refinement rates (Fig. 2q). However, sequential injections in the same mouse require extended animal manipulation, and while counting absolute numbers is precise, this method requires the accurate definition of the area of interest at all stages of development^36^. Delimiting areas, such as S2, V1, or V2, where specific cytoarchitectures only develop at later stages, can be difficult and may lead to errors in the measurements^36^. Therefore, although quantification of CTB-labeled CPN as percentages of cell nuclei includes all cell types in the denominator, we considered this method as the most appropriate for comparing the dynamics of refinement between functional areas and layers (Fig. 1k–o)^35^.

Injections in the CC indicated that the reductions in the number of CTB^+^ cells observed throughout postnatal development are the result of refinement of existing neuronal projections. For each layer within the same area (S1 or V1), final connectivity is established at different rates and terminates at different postnatal stages (Figs. 1k–o and 2d–i, p). For example, the proportion of CTB^+^ S1L4 neurons, which are only locally connected in the adult^37^, presents a maximum labeling of nearly all neurons within the layer at P3, and decreases sharply and steadily during development until P21, when only a few callosal projections are left (Figs. 1l and 2f). Refinement of L2/3 neurons, on the other hand, terminates earlier, namely by the second postnatal week (Figs. 1k and 2e), resulting in a much higher proportion of CPN^10^. CTB labeling in L5A, L5B, and L6 also indicates refinement, but this refinement concludes within the first postnatal week (Figs. 1m–o and 2g–i). Altogether, these results show, in agreement with previous studies, that subsets of cortical neurons from all layers extend transient exuberant interhemispheric axons during development that are later refined. However, they indicate much higher numbers of early CPN and higher refinement rates than previously reported.

### Visualization of S1L4 transient callosal projections

**Fig. 3:**
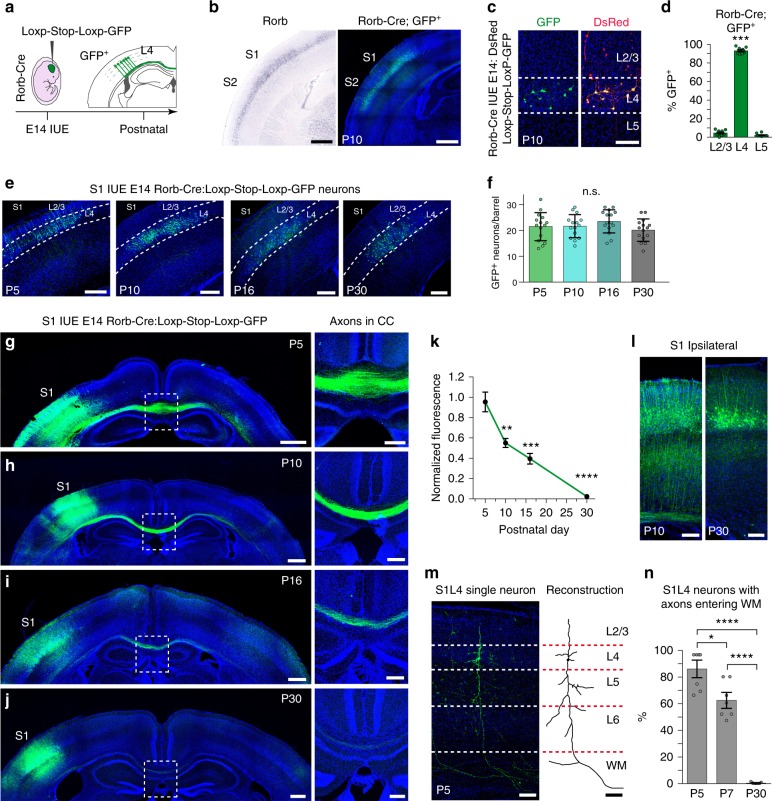
Transient callosal projections extend from S1L4 Rorb neurons. **a** Electroporation of Rorb-Cre embryos with a floxed-GFP plasmid at E14. **b** Left panel, expression of endogenous Rorb expression (Image credit: Allen Institute). Right panel, GFP (green) in a P10 Rorb-Cre animal electroporated at E14 in S1 and S2. DAPI (blue). **c** GFP illuminates a subset of L4 neurons, while DsRed labels all electroporated neurons after co-electroporation at E14. **d** Layer distribution of total Rorb-GFP^+^ cells in S1 (*n* = 850 neurons, *n* = 2 sections per animal, *n* = 5 animals). ***P value_GFP+ cell/layer_ ≤0.0001 (one-way ANOVA (*n* = 5), *F*_GFP+ cell/layer_ (2, 18) = 2629). **e**, **f** Images and quantifications of the number of GFP^+^ cells per barrel after analysis at the indicated stage (*n* = 5 barrels, *n* = 12 mice total; *n* = 3 mice for each postnatal stage). (n.s.) *P* value_GFP+/postnatal stage_ = nonsignificant (two-way ANOVA (*n* = 12): *F*_GFP_^+^_/postnatal stage_ (3, 56) = 1.34). **g–j** Coronal sections of electroporated Rorb-Cre brains. Right panels show magnification of the outlined box areas. GFP (green) and DAPI (blue). **k** Quantification of the ratio of GFP signal in CC to ipsilateral GFP signal of somas, normalized to mean value at P5 (*n* = 3 mice per stage). *P* value_Fluorescence/time_ ≤ 0.0001 (two-way ANOVA (*n* = 12), *F*_Fluorescence/time_ (3, 6) = 108.8), *p* value _vs. P5_ **≤0.01, ***≤0.001, and ****≤0.0001 (post hoc with Tukey’s test). **l** S1L4 ipsilateral projections in P10 and P30 brains. **m** Single neuron GFP labeling in P5 brains show S1L4 neurons with axons entering the white matter (WM). Reconstruction of that same neuron shown in right panel. **n** Percentage of S1L4 single neurons with axons entering the WM (*n* = 80 neurons, *n* = 2 sections per animal, *n* = 3 animals per stage). *P* value_% axons/time_, ≤0.0001 (two-way ANOVA (*n* = 9): *F*_% axons/time_ (2, 12) = 59.02). *P* value *≤0.05; ****≤0.0001 (post hoc with Tukey’s test). Scale bars: 500 µm (**b** and **g–j**, left panels), 200 µm (**e** and **g–j** right panels), and 100 µm (**c**, **l**, and **m**). Data show mean ± SEM (error bars) (**d**, **f**, **k**). Source data are provided as a Source Data file

The CTB injections indicated the existence of widespread transient callosal projections, including projections from several populations that have been reported to have limited contralateral projections. However, we could not fully discard caveats in the methodology, such as, for example, possible transneuronal processing of CTB or differences in CTB uptake due to heterogeneity in myelination. Importantly, one of the most striking observations was evidence of transient callosal projections in the majority of L4 neurons. We, therefore, aimed to unequivocally demonstrate the existence of these transient callosal projections. S1L4 neurons provide an excellent experimental subpopulation for this purpose since virtually all of them lose their interhemispheric projections according to our CTB injections (Figs. 1l and 2f, p). The use of a knock-in *Rorb* allele that directs Cre recombinase expression in L4 cells (Rorb-Cre)^38^, combined with in utero electroporation (IUE) at embryonic day (E) 14 of a floxed green fluorescent protein (GFP) plasmid, allowed the selective illumination of S1L4 somas and axons (Fig. 3a–d). Analysis of electroporated brains at P5, P10, P16, and P30 demonstrated comparable number of GFP^+^ cells in all animals, indicating similar electroporation efficiency and undistinguishable neuronal survival rates (Fig. 3e, f). Most notably, brains at P5 and P10 revealed GFP axons crossing the midline through the CC and extending contralaterally (Fig. 3g, h). These S1L4 callosal projections were greatly reduced in P16 animals (Fig. 3i), and very few axons were detected at P30 (Fig. 3j). Quantifications of GFP axons in the CC at midline showed that the refinement of these developmental callosal projections was significant (Fig. 3k). These transient S1L4 callosal axons likely retract or disassemble, as we did not observe major changes in the number of GFP^+^ neurons that would indicate neuronal death, or broken GFP axonal fragments in the CC or cortical plate (Fig. 3e–j) during their period of refinement. We also found that GFP^+^ S1L4 neurons exhibit ipsilateral projections with radial trajectories at early postnatal stages but not at the mature P30 stage (Fig. 3l). Morphological reconstructions of single GFP^+^ S1L4 neurons followed by quantifications demonstrated that L4 neurons possess early axons that enter the WM, and then they lose these projections during postnatal development (Fig. 3m, n). Hence, analysis of the GFP^+^ S1L4 population demonstrated both extension and refinement of transient callosal axons from P5 to P30 that paralleled the results we observed with CTB injections in the CC and that reflected an unreported remodeling of S1L4 axons. Taken together, these results demonstrate that during development the majority of L4 excitatory neurons of the S1 cortex send exuberant transient interhemispheric axonal projections that are gradually refined to give rise to the known adult connectivity.

### S1L4 transient callosal axons fail to terminate innervation

**Fig. 4:**
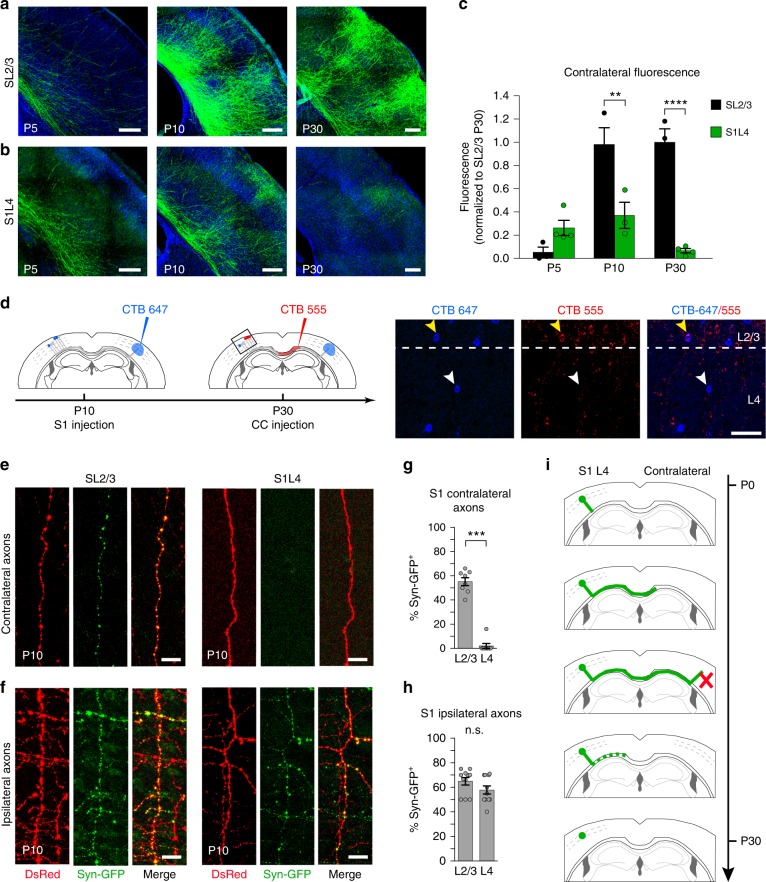
Transient S1L4 callosal axons do not progress to terminal innervation. **a**, **b** Comparison of the dynamics of contralateral SL2/3 and S1L4 axons. SL2/3 axons labeled with GFP progress to terminal innervation. S1L4 contralateral axons labeled with GFP following E14 IUE in Rorb-Cre mice are unable to form contralateral columns. **c** Quantification of GFP axons in the contralateral plate as shown in **a**, **b**. Values are normalized to the mean fluorescence of SL2/3 at P30. Mean ± SEM (error bars) (*n* = 3 animals per stage and condition, *n* = 18 mice total). *P* value_SL2/3/S1L4_ ≤0.000, *p* value_Fluorescence/time_ ≤0.0001 (two-way ANOVA (*n* = 18): *F*_SL2/3/S1L4_ (1, 14) = 39.19, *F*_Fluorescence/time_ (2, 14) = 18.96). *P* value **≤0.01; ****≤0.0001 (post hoc with Tukey’s test). **d** Scheme of experiments performing sequential injections of CTB in the cortical plate and CC. The boxed area indicates the approximated location of S1 region corresponding to images shown on the right. White arrows indicate an L4 neuron of a P30 brain that pruned its P10 contralateral projection (blue only neuron); yellow arrows indicate an L2/3 neuron that reached the contralateral plate at P10 and stabilized this projection (blue and red). **e** Images of axons from SL2/3 or S1L4 neurons electroporated with CMV-synaptophysin-GFP (Syn-GFP; green) and pCAG-DsRed (DsRed; red) invading the contralateral plate. **f** Ipsilateral axons extending from SL2/3 or S1L4 neurons electroporated with CMV-synaptophysin-GFP and DsRed. **g**, **h** Quantifications of the percentage of contralateral axons (**g**) or ipsilateral axons (**h**) containing synaptophysin. Mean ± SEM (error bars) (*n* = 100–150 axons per condition, *n* = 3 animals per condition). ***P value ≤0.001 and (n.s.) non significant (*χ*^2^ test). **i** Working model of S1L4 axonal refinement during postnatal development. Scale bars represent 200 µm (**a**, **b**), 50 µm (**d**), and 10 µm (**e**, **f**). Source data are provided as a Source Data file

In order to understand why some axons remain callosal and others do not, we compared the branching and synaptic behavior of GFP^+^ S1L4 transient exuberant axons to callosal axons of the SL2/3 population, which are capable of integrating in the adult and can be visualized during development after IUE of a GFP plasmid at E15 (Fig. 4a)^10,39,40^. SL2/3 and S1L4 callosal axons demonstrated distinct behaviors in the contralateral cortical plate (Fig. 4a–c). While GFP^+^ SL2/3 branching is appreciable in the cortical plate after P5 (Fig. 4a, c), we never detected more than a few GFP^+^ S1L4 axons ascending into the contralateral cortical plate, and the number of these axons decreased after P10 (Fig. 4b, c). Further, sequential CTB injections in the cortical plate at P10 and in the CC at P30 detected S1L4 neurons that had callosal axons that invaded the contralateral cortical plate at P10 and were later pruned (Fig. 4d, Supplementary Fig. 3). Thus, S1L4 callosal axons can reach contralateral targets, but unlike SL2/3 axons, they do not accumulate contralaterally. This likely explains the low frequency of S1L4 labeling by CTB injections in the developing cortical plate (Supplementary Fig. 1i–l) and why these exuberant projections had not been noted in previous studies^13–18,25^. We also found that at P10, synaptophysin clusters are absent from nearly all GFP^+^ S1L4 axons found in the contralateral cortical plate (Fig. 4e, g), but present in S1L4 ipsilateral branches (Fig. 4f, h), as well as in their P10 SL2/3 ipsilateral and contralateral counterparts (Fig. 4e–h). These results indicate that S1L4 neurons do not establish synapses in contralateral territories. Collectively, these data show that transient exuberant L4 callosal projections are capable of invading the contralateral plate but are unable to synapse and terminate innervation in this area (Fig. 4i).

### Sensory thalamic input determines L4 callosal connectivity

**Fig. 5:**
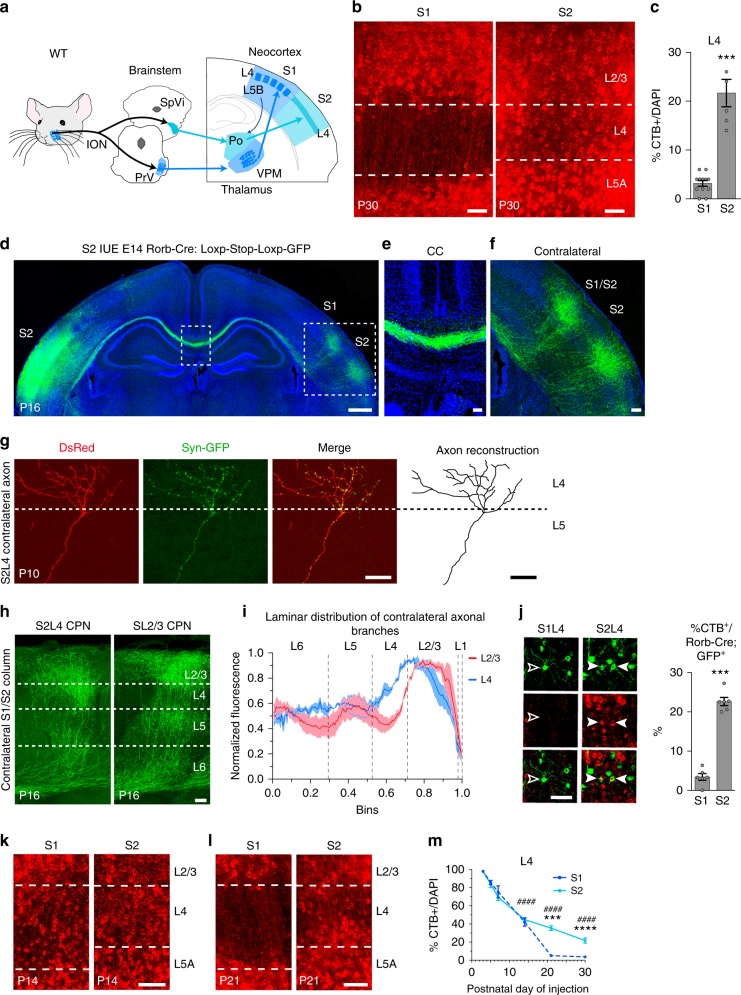
S2L4 Rorb^+^ neurons establish contralateral connectivity in somatosensory cortex. **a** Schematic representation of S1 and S2 circuits. ION = infraorbital nerve, PrV = principal trigeminal nuclei, SpVi = spinal trigeminal nuclei, Po = posterior nucleus, VPM = ventral posteromedial nucleus. Dark shaded blue dots and boxes represent whisker topographic fields. **b** CTB^+^ neurons after callosal injections at P30. **c** Quantifications of CTB^+^/DAPI cells (*n* = 300 cells, *n* = 2 sections per animal, *n* = 3 animals per condition). ***P value ≤0.001 (Student’s *t* test). **d–f** Coronal section of a P16 Rorb-Cre mouse electroporated in S2. Boxes indicate areas magnified in **e**, **f**). S1/S2 indicates the border between both areas (**f**). **g** Syn-GFP expression in a DsRed-labeled S2L4 axon found in the contralateral plate of a P10 Rorb-Cre brain. **h** GFP^+^ contralateral axonal columns formed by S2L4 or SL2/3 neurons at S1/S2 border. **i** Normalized GFP signal along the radial axis of the cortical plate (WM, bin 0; pial surface, bin 1) in S1/S2 columns as in **h**, (143 measurements, *n* = 3 mice per condition). *P* value_SL2/3/S2L4_ ≤0.0001 (two-way ANOVA (*n* = 6): *F*_SL2/3/S2L4_ (1, 572) = 56.71). **j** Rorb-Cre;GFP^+^ neurons after CTB injections at P30. Outlined and white arrowheads indicate CTB^−^ and CTB^+^ neuron, respectively. At right, quantifications (*n* = 400 neurons per condition, *n* = 2 sections per animal, *n* = 3 animals). ***P value ≤0.001 (Student’s *t* test). **k**, **l** CTB^+^ neurons after callosal injections at P14 (**k**) and P21 (**l**). **m** Refinement dynamics (*n* = 300 cells, *n* = 2 sections per animal, *n* ≥ 3 animals per stage) (dashed line, data shown in Fig. 1l). *P* value_S1/S2_ ≤0.0001, *p* value_CTB+/time_ ≤0.0001 (two-way ANOVA (*n* = 24): *F*_S1/S2_ (1, 53) = 20.82, *F*_CTB+/time_ (4, 53) = 216.4). *P* value_S1 vs. S2_ ***≤0.001; ****≤0.0001, *p* value_S2CTB+ vs. S2 CTB+ at P5 _^####^≤0.0001 (post hoc with Tukey’s test). Scale bars: 500 µm (**d**), 100 µm (**f**, and **h**), and 50 µm (**b**, **e**, **g**, **j**, **k**, and **l**). Data show mean ± SEM (error bars). Source data are provided as a Source Data file

Sensory information from the facial whiskers and the whisker pad are integrated into the SS cortex by the lemniscal and paralemniscal pathways. While S1L4 is the main target of the lemniscal pathway via the ventral posteromedial nucleus (VPM) thalamocortical axons, S2L4 is mainly innervated by the medial posterior nucleus (Po). The VPM primarily conveys input from the principal trigeminal nuclei (lemniscal pathway) and the Po conveys input from the spinal trigeminal nuclei (paralemniscal pathway) (Fig. 5a). Even though these two trigemino-thalamo-cortical pathways share many common afferents, S1L4 and S2L4 neurons are differentially activated by tactile and nociceptive stimuli. While S1L4 neurons respond to tactile stimuli in vivo and are not activated by nociceptive input from the whisker pad, S2L4 neurons respond to both nociceptive and tactile stimuli^5,41,42^. Hence, S1L4 and S2L4 neurons exhibit distinct functional integration into the cortical circuit.

CTB and IUE experiments demonstrated that S1L4 neurons do not maintain their callosal projections, but CTB injections did indicate that a fraction of S2L4 neurons preserves their interhemispheric axons (Fig. 5b, c). We therefore performed IUE to selectively target S2 in Rorb-Cre mice. Visualization of GFP^+^ neurons revealed that mature projections from S2L4 establish homotopic columns in the contralateral somatosensory cortex (Fig. 5d–f and Supplementary Fig. 4b, c), and showed that they are capable of branching and forming synapses in the contralateral territory (Fig. 5g) in agreement with CTB labeling. The positions of these contralateral columns are the same as the reported locations of SL2/3 callosal columns (Supplementary Fig. 4a–c)^39,40^, but S2L4 CPN innervate distinct cortical layers (Fig. 5h, i). Thus, S2L4 neurons integrate into specific interhemispheric circuits that are distinct from those formed by SL2/3 neurons. Quantifications of CTB^+^ neurons in S1 and S2 GFP^+^ neurons after CC injections at P30 of Rorb-Cre-electroporated animals demonstrated that GFP^+^ contralateral branches originate from a neuronal subpopulation located almost exclusively in S2L4 (Fig. 5j, Supplementary Fig. 4d). Interestingly, analysis of CTB distributions during development revealed that S1L4 and S2L4 initially follow identical kinetics of callosal refinement, and that mature S2L4 CPN arise from reduced refinement of their interhemispheric projections after P14 (Fig. 5k–m). Similar developmental dynamics of S2L4 axons were observed in IUE Rorb-Cre brains (Supplementary Fig. 4e–h). These results describe previously unreported interhemispheric connectivity of L4 neurons and demonstrate how the distinct outcomes of callosal refinement in S1L4 and S2L4 shape two different cortical circuits. They suggested that functional input may play an integral role in callosal L4 selection.

Each primary and secondary sensory areas of the cortex receive input from sensory organs via their first-order and higher-order thalamic nuclei. Our data revealed area-specific differences in dynamics of refinement, not only between S1L4 and S2L4 (Fig. 5m, Supplementary Fig. 4e–h) but also between V1 and V2 areas (Supplementary Fig. 5), and between different sensory areas (S1 vs. V1; Fig. 1k–o). On the other hand, we observed few differences in the distributions of CPN in auditory areas (A1 vs. A2; Supplementary Fig. 6), which exhibit less topographic and functional segregation in the cortex and thalamus than visual and somatosensory circuits^5,43^. Together, these data strongly suggested that distinct inputs from different nuclei define the outcome of the default callosal projections extended by cortical neurons.

**Fig. 6:**
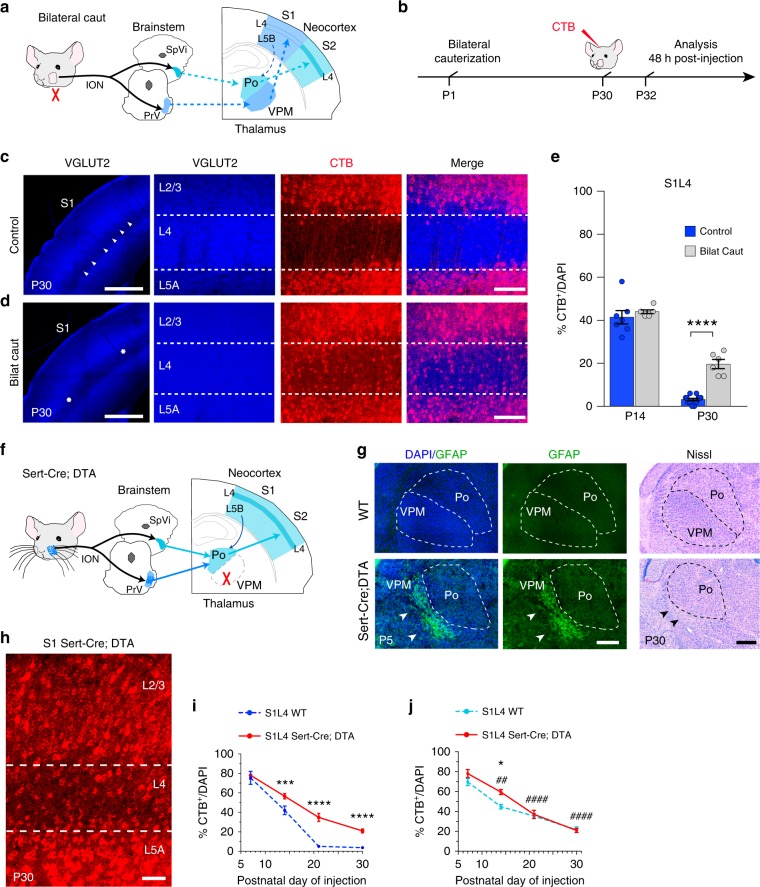
Sensory input determines terminal stabilization of L4 callosal projections. **a**, **b** Scheme of whisker ablation experiments. ION = infraorbital nerve, PrV = principal trigeminal nuclei, SpVi = spinal trigeminal nuclei, Po = posterior nucleus, VPM = ventral posteromedial nucleus. **c**, **d** VGlut2 staining in control (**c**) and bilaterally cauterized animals (**d**). Arrowheads demarcate barrels. Stars delineate presumptive barrelfield. In red, CTB labeling. **e** Quantifications (*n* = 300 cells, *n* = 2 sections per animal, *n* = 3 animals per stage and condition). *P* value_control vs. bilateral_ ≤0.0001 (two-way ANOVA (*n* = 12): *F*_control vs. bilateral_ (1, 25) = 24.51). ****P value_control vs. bilateral_ ≤0.0001 (post hoc with Tukey’s test). **f** Scheme depicting genetic elimination of VPM. **g** Left and center panels show GFAP staining. Glial scars indicated by white arrowheads mark neuronal loss. At right, Nissl staining of P30 somatosensory thalamic nuclei in WT and Sert-Cre;DTA. In Sert-Cre;DTA the VPM is lost (black arrowheads). **h** CTB^+^ in S1L4 of P30 Sert-Cre;DTA animals. **i** Refinement dynamics of S1L4 projections from WT and Sert-Cre;DTA (*n* = 300 cells, *n* = 2 sections per animal, *n* ≥ 3 animals per stage and condition) (dashed line indicates data also shown in Fig. 1l). *P* value_WT/Sert-Cre;DTA_ ≤0.0001, *p* value_%CTB+/time_ ≤0.0001 (two-way ANOVA (*n* = 18): *F*_WT/Sert-Cre;DTA_ (1, 46) = 59.62, *F*_%CTB+/time_ (3, 46) = 156.1). *P* value _WT vs. SertCre;DTA_ ***≤0.001; ****≤0.0001 (post hoc with Tukey’s test). **j** Refinement dynamics of S2L4 projections from WT and S1L4 Sert-Cre;DTA (*n* = 300 cells, *n* = 2 sections per animal, *n* = 3 mice per stage and condition) (dashed line indicates data shown in Fig. 5m). *P* value_WT vs. Sert-Cre;DTA_ ≤0.05, *p* value_%CTB+/time_ ≤ 0.0001 (two-way ANOVA (*n* = 24): *F*_WT vs. Sert-Cre;DTA_ (1, 36) = 7.769, *F*_%CTB+/time_ (3, 36) = 99.39). *P* value_P14 WT vs. P14 SertCre;DTA_ *≤0.05, *p* value_Sert-Cre;DTA vs. P7 Sert-Cre;DTA_^##^≤0.01, ^####^≤0.0001 (post hoc with Tukey’s test). Scale bars: 500 µm (**c**, **d**, left panels), 100 µm (**c**, **d**, right panels, and **g**), and 50 µm (**h**). Data show mean ± SEM (error bars). Source data are provided as a Source Data file

We decided to directly investigate the role of sensory input in CPN selection and to explore the potential for plasticity and terminal connectivity of exuberant developmental callosal projections. We first tested the effect of manipulating sensory-evoked activity. We again focused on S1L4 because it is a direct recipient of sensory information from the whiskers, and their cauterization eliminates both transmission of sensory-derived activity to S1L4 and the topographic assembly of S1 circuits (Fig. 6a, b, Supplementary Fig. 7a–d)^37,40,44^. We found that unilateral cauterization reduces the number of S1L2/3 CPN as a consequence of the unbalanced activity between the two hemispheres as reported^40^, but does not measurably affect the low number of CPN in S1L4 (Supplementary Fig. 7). In contrast, we observed that bilateral whisker cauterization did not significantly affect the number of S1L2/3 CPN (Supplementary Fig. 7h)^40^, but it resulted in a marked increase in S1L4 CPN compared to control animals (20% compared to 3.5%) (Fig. 6c–e). The increase in the number of S1L4 CPN in cauterized animals was the result of reduced refinement rates, specifically after P14 (Fig. 6e). These experiments demonstrate the instructive role of sensory-evoked activity in CPN selection.

We speculated that we could alter the terminal fate of S1L4 callosal projections by changing the sensory nucleus of origin of their thalamic innervation, and thus their attributed input type, using a genetic approach. S1 receives primary tactile information from whiskers via the VPM entering at L4, while S2 receives nociceptive information through the Po (Fig. 5a). Targeted activation of a floxed diphtherotoxin (DTA) allele using serotonin transporter (Sert) as a genetic driver for the recombinase Cre (Sert-Cre mice) results in ablation of VPM neurons (Sert-Cre;DTA mice) (Fig. 6f, g)^45^. This triggers plasticity in the higher-order Po neurons, which then project to S1L4 neurons in addition to S2L4, and causes them to process both tactile and nociceptive information (Fig. 6f)^30,46^. We found that this input switch led to an increase (to 20%) in the number of S1L4 CPN (Fig. 6h, i). This increase was the result of a sustained reduction in refinement during postnatal development (Fig. 6i) and resulted in a more S2-like pattern of refinement (Fig. 6j). These experiments, in which input was either obstructed or switched, together demonstrate that the information that instructs callosal refinement is conveyed by first-order and higher-order sensory nuclear inputs, likely in the form of intrinsic and evoked activity. These manipulations exhibit the plasticity of S1L4 exuberant callosal projections and demonstrate their capacity to integrate into the mature circuit.

### Plasticity of the early callosal fate of cortical neurons

**Fig. 7:**
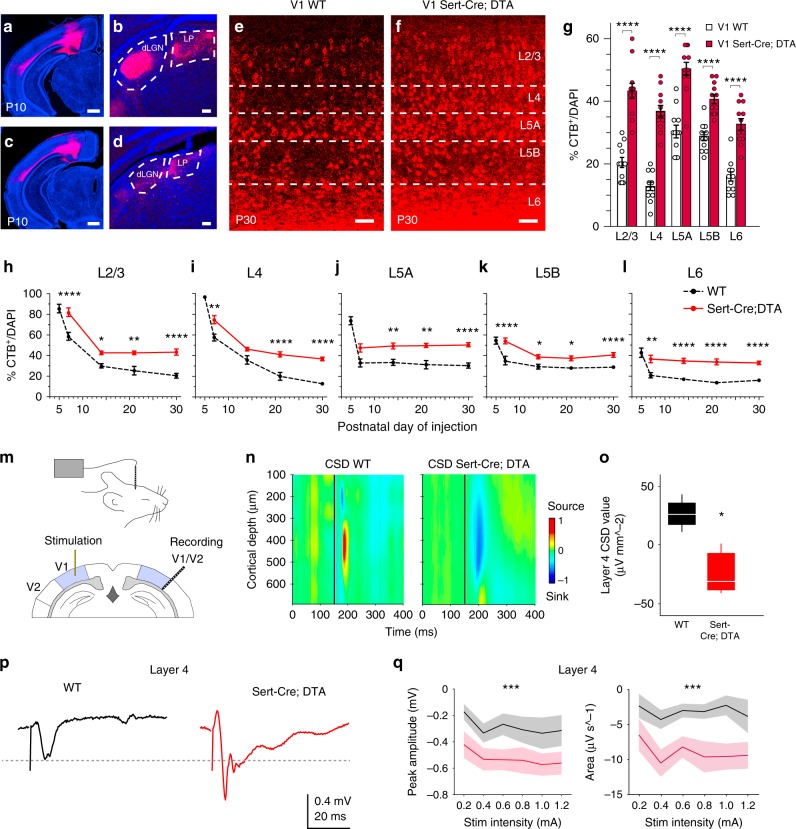
Elimination of thalamic input increases interhemispheric connectivity in the visual cortex. **a–d** Cortex and thalamus of WT (**a**, **b**) and Sert-Cre;DTA (**c**, **d**) after injections of CTB in V1 cortex. Dashed lines delineate thalamic nuclei (**b**, **d**). **e**, **f** CTB^+^ neurons (red) in P30 V1 cortex after callosal injections. Scale bars: 500 µm (**a**, **c**) and 100 µm (**b**, **d**–**f**). **g** Quantifications of CTB^+^ cells after CC injections in adult mice (*n* = 6 animal per condition) (WT data from Fig. 1). ****P value _WT vs. SertCre;DTA_ ≤ 0.0001 (two-way ANOVA, post hoc with Tukey’s test). **h**–**l** Refinement dynamics in V1 (*n* = 300 cells, *n* = 2 sections per animal, *n* ≥ 3 mice per stage and condition). Dashed line (WT data from Fig. 1k–o). No data from P3 or P5 Sert-Cre;DTA mice were collected because at these stages, and mutants recover poorly after the injections. *P* value_WT vs. Sert-Cre;DTA_ ≤0.0001 (**h**–**l**) (two-way ANOVA (*n* = 33): *F*_WT vs. Sert-Cre;DTA_ (1, 50) = 102.7 (**h**), *F*_WT vs Sert-Cre;DTA_ (1,5 0) = 100.6 (**i**); *F*_WT vs. Sert-Cre;DTA_ (1, 50) = 75.88 (**j**); *F*_WT vs. Sert-Cre;DTA_ (1, 50) = 83.64 (**k**); *F*_WT vs. Sert-Cre;DTA_ (1, 50) = 119.7 (**l**)). *P* value_WT vs. Sert-Cre;DTA_ *≤ 0.05, **≤0.01, and ****≤0.0001 (post hoc with Tukey’s test). **m** Scheme of electrophysiological recordings. **n** Representative in vivo current–source density (CSD) distribution of evoked neuronal activity recorded in V1/V2 after stimulation in contralateral V1. **o** Box plot of L4 CSD values. Central mark: median; box limits: upper and lower quartiles; whiskers: minimum and maximum values. *P value_WT vs. Sert-Cre;DTA_ ≤0.05 (one-way ANOVA: *F*_WT vs. Sert-Cre;DTA_ (1, 6) = 14.4). **p** Representative evoked local field potential traces recorded in L4. **q** Mean values of peak amplitude and area (*n* = 9 replicates per mouse, *n* = 3 WT mice, and *n* = 4 Sert-Cre;DTA mice (including WT and Sert-Cre;DTA littermates)). ***P value_WT vs. Sert-Cre;DTA_≤ 0.001 (three-way ANOVA (*n* = 7): *F*_WT vs. Sert-Cre;DTA amplitude_ (1, 124) = 4.25; *F*_WT vs. Sert-Cre;DTA area_ (1, 124) = 10.91). Data show mean ± SEM (shaded) (**g**, **h**–**l**). Source data provided as a Source Data file

The plasticity we observed in S1L4 neurons suggested that there exists broad potential for terminal callosal connectivity in L4 cortical neurons. We set out to explore this possibility in the context of a drastic loss of innervation by again utilizing Sert-Cre;DTA animals and focusing on the visual cortex. Sert is expressed in visual thalamic nuclei^44,45^. Histology of Sert-Cre;DTA brains showed depletion of both the first-order lateral geniculate nucleus and of the higher-order visual lateroposterior nucleus (Supplementary Fig. 8). This correlated with the extensive depletion of ascending thalamic input in V1 Sert-Cre;DTA mutants compared to wild-type (WT) (Fig. 7a–d). Analysis of the visual cortex of P30 Sert-Cre;DTA animals demonstrated no neuronal death and showed that, overall, lamination and early molecular specifications (Satb2 and Ctip2 expression) are not altered, while expression of postmitotic thalamic-dependent transcription factors (Cux1 and Rorb)^44^ is reduced (Supplementary Fig. 9). CTB injections in the CC showed that lack of visual thalamic input results in significantly increased number of CPN in all V1 layers (almost two-fold in total) compared to WT (Fig. 7e–g) or to control heterozygous mice (Supplementary Fig. 10). Notably, the largest increase was observed in L4, where 40% of V1L4 neurons showed callosal axons in mutants (a 2.8-fold increase compared to WT) (Fig. 7g). This dramatic increase likely reflects the immediate synaptic connections of L4 neurons with the thalamus. Comparisons of the developmental dynamics of callosal projections indicated that the differences between Sert-Cre;DTA and WT mice originate from early stages of development, as the number of CPN was higher in mutants at all comparable stages of the analysis (Fig. 7h–l). From P7 on, CPN refinement follows almost parallel dynamics in most layers of mutant and WT mice (Fig. 7h–l), underscoring a role for neuronal intrinsic information in CPN selection^10,12^. In V1L4, the differences in CPN number continue to increase after P15, possibly reflecting the effect of eye-opening and visually evoked activity in WT animals at P14 (Fig. 7i). These increases in CPN numbers show that cortical neurons are capable of sustaining and stabilizing their exuberant developmental callosal axons when thalamic input is reduced.

Because CTB injections revealed callosal hyperconnectivity in V1 of Sert-Cre;DTA animals, we tested whether this increased structural connectivity extrapolated to increased functional connectivity, particularly in L4 where we observed the largest increases in CPN. Interhemispheric-evoked activity was evaluated by measuring local field potentials using a multielectrode array inserted in the V1/V2 border area in WT and Sert-Cre;DTA animals after local stimulation of the contralateral V1 (Fig. 7m). Analysis of those responses showed changes in the current–source density (CSD) profile in Sert-Cre;DTA compared to WT mice (Fig. 7n). Analysis of the values per individual layer demonstrated increased interhemispheric response in V1L4 (Fig. 7o). Further examination of the peak amplitudes and areas demonstrated significant hyperexcitability and increased interhemispheric responses in all layers of Sert-Cre;DTA mice (Fig. 7p, q, Supplementary Fig. 11). Altogether, these data demonstrate that L4 callosal projections detected in Sert-Cre;DTA animals are able to functionally integrate into and contribute to a non-canonical network.

In sum, our experiments demonstrate both extension and refinement of L4 cortical neurons. Moreover, we demonstrate that transient callosal axons possess the potential to establish bona fide mature callosal connections. The results show that cortical L4 neurons achieve their local or long-range connectivity due to activity-dependent refinement of initial exuberant callosal projections.

## Discussion

Neuronal wiring is a key concept in our understanding of brain functions and pathologies, as well as our own diversity. However, much remains to be elucidated regarding how networks are specified during development. Here we report a previously unrecognized developmental step that precedes the formation of mature L4 local circuitries. We demonstrate that L4 cortical neurons extend transient callosal axons that are subsequently eliminated. Importantly, we also demonstrate that these neurons, which are paradigms of locally connected neurons, are capable of remaining callosal, maturing, and forming functional connections in response to changes in thalamic input. Together these indicate that the development of these exuberant axons allows a plasticity, both structural and functional, that translates thalamic inputs into differential wiring efficiently, and that endows the cortex with potential to generate alternative circuits in non-canonical scenarios.

In addition to the early abundance of callosal projections of L4 neurons, our CTB injections in the CC identified numerous callosal neurons in all cortical layers. These callosal axons are subsequently refined to degrees and at rates that vary depending on the layer and area of the cortex. The number of these developmental exuberant projections appear greater than those reported by earlier retrotracing injections in the cortical plate of rodents and other mammals^13–17,19,20,24^. However, even in these earlier studies, it was noted that more neurons were labeled by the retrotracer in those animals in which injections were performed closer to the WM than in those in which injections were limited exclusively to the gray matter^18,19^. These high CPN numbers do not seem to result from unspecific labeling. We assessed the selectivity and efficiency of our WM injections (see Methods and Supplementary Figs. 1 and 2, and Supplementary Methods) and found results supporting the specificity of the CTB labeling, such as lack of labeling of L2/3 neurons at P1 (Supplementary Fig. 2d), or fewer labeled L2/3 neurons at P3 than at P5 (Figs. 1k and 2e). Further, the patterns and distributions of CPN we observed in the adult are in accordance with the known connectivity^10,37^. Nevertheless, it is possible that our injection methodology could result in some degree of promiscuous or transneuronal labeling, or neurons could take up CTB with higher efficiencies at younger stages or depending on the degree of axonal myelinization. Hence, we cannot discard that these and other unforeseen methodological issues could affect the reported refinement rates. Thus, the CTB labeling and refinement dynamics we report for layers other than L4 should be confirmed by independent methods. In contrast, the use of Rorb-Cre mice allowed us to visualize the presence of L4 transient axons, thus providing a highly reliable and independent confirmation of the results we obtained for L4 populations in our CTB experiments.

We demonstrate that the mature callosal and non-callosal fates of L4 neurons in sensory areas are accomplished through the action of thalamic input. We also show that L4 exuberant callosal axons are capable of being stabilized, which indicates an uncommitted fate of early postmitotic L4 populations. This is an important mechanistic insight because it shows that the unfolding of L4 fates progresses by discarding already primed alternative connectivity in a context-dependent manner. In this regard, we did not assess whether final adult connectivity emerges from the selective elimination of callosal axons and the concomitant stabilization of other projections, or from the elimination of failed callosal connections followed by the growth of new projections to other targets. In either case, the extension of early exuberant projections helps to explain how intrinsic or extrinsic changes can be rapidly translated into the neurons alternative wiring and why postmitotic neurons show a greater capacity for reprogramming at early stages of differentiation^47,48^. The identification of L4 exuberant callosal projections also indicates that these neurons possess the required genetic information to extend and guide axons contralaterally^49–51^.

The initial potential for callosal connectivity of L4 neurons is in line with recent reports proposing that the early molecular fate of neurons becomes gradually restricted during differentiation, rather than being fixed at birth^30,33,34,52^ and challenge the idea that the subsets of non-callosal cortical neurons are established prior to axonal extension. They are also in accordance with earlier interpretations that suggested that axonal elimination of exuberant CPN enables a variety of circuits while minimizing the necessity to encode information early in development^14,15,18,19,21,22,25^. Molecularly, transcription factors such as Brn1/2, which inhibits Rorb and several L4 characteristics in L2/3^53^, or Foxg1, required for interhemispheric connections^54^, might be involved in the maturation of L4 neurons as callosal or ipsilateral-only connected neurons in specific sensory circuits (see also Supplementary Discussion).

The mechanisms described here provide a novel understanding of how L4 connectivity and mature fate is achieved and broaden our perspective of how the brain wires. They help explain how non-canonical circuits can emerge in the context of neurodevelopmental diseases or, as a consequence of abnormal extracortical circuits, impaired sensory integration^9,17,40,55–57^, early damage of sensory organs^58^, or defects in neuromodulatory signals during development^59,60^, and suggest new therapeutic possibilities based on this cortical exuberance and plasticity.

## Methods

### Mice

The morning of the day of the appearance of a vaginal plug was defined as E0.5. *Slc6a4tm1(cre)Xz/J* (Sert-Cre) mice (Jax stock #014554 and #009669), and *R26:lacZbpA(flox)DTA* (DTA) heterozygous mice^61^ were maintained in heterozygosis in the C57BL/6JRccHsd background (Envigo laboratories, formerly Harlan). Sert-CRE;DTA mutants were obtained crossing heterozygous mice. WT animals were obtained from these colonies. *Rorb-IRES2-Cre-D* knock-in (Rorb-Cre) mice were obtained from Jackson Mice repository (Jax stock #023526) and maintained in heterozygosis in the C57BL/6JRccHsd background. In this mouse Cre recombinase expression is directed by Rorb-expressing cells, without disrupting endogenous RAR-related orphan receptor β-beta expression^38^. Animals were housed and maintained following the guidelines from the European Union Council Directive (86/609/ European Economic Community). All procedures for handling and sacrificing animals complied with all relevant ethical regulations for animal testing and research. All animal procedures followed the European Commission guidelines (2010/63/EU) and were approved by the CSIC and the Community of Madrid Ethics Committees on Animal Experimentation in compliance with national and European legislation (PROEX 118-14; 233/16;124-17; 234-16; 123-17; 065/19).

### CTB injections for retrograde labeling

Retrograde labeling from the CC was performed by injections of CTB subunit conjugated to Alexa Fluor 555 or 647 (Invitrogen C-34776 and C-34778, Thermo Fisher Scientific). An ultrasound guided back-scatter microscopy and injection guidance system (Vevo 770, VisualSonics, Toronto) was used to inject into the CC close to the midline in P3, P5, and P7 animals (230 nl per injection site). The earliest time to clearly visualize the CC by ultrasound was P3 for the rostral somatosensory areas, and P5 for caudal visual areas. Stereotaxic coordinates were also used to inject animals of all stages. For this, animals were anesthetized during the surgical procedure with isoflurane/oxygen and placed on a stereotaxic apparatus (Harvard Apparatus, UK). The injections were made directly into the CC at an angle of 18°. For ultrasound and stereotaxic injections, CTB particles (0.5% in phosphate-buffered saline (PBS)) were injected with a Drummond Nanoject II Auto-Nanoliter Injector using 30 mm pulled glass micropipettes (Drummond Scientific Co. 3000205A and 3000203G/X). To minimize damage, injections were made progressively at 23 nl per injection pulse with a maximum frequency of one pulse per second (hence 23 nl s^−1^) until the desired total volume was achieved. Although we cannot disregard that some injury may occur during our craniotomies and injection procedures, we never detected obvious signs of relevant damage in the cortical plate or CC or in callosal GFP axons after our injections. The total volume for each stage was experimentally determined as the minimum volume required to maximize labeling of the area of interest as described in detail in the Supplementary Methods (CTB injections). These volumes are 230 nl (P1–P7), 460 nl (P14), or 575 nl (P21–Adult). Coordinates (mm) in the antero-posterior (AP), medio-lateral (ML), and dorso-ventral (DV) axes were determined from Bregma. For the analysis of somatosensory CPN, coordinates were: for P1 (−1.1 AP; +0.4 ML and −0.9 DV), for P3–P5 (−1.2 AP; +0.4 ML and −1.3 DV); for P7 (−1.2 AP; +0.5 ML and −1.6 DV); for P14 (−1.4 AP; +0.7 ML and −1.8 DV); for P21–Adult (−1.4 AP; +0.7 ML and −2 DV). For the analysis of visual and auditory CPN coordinates were: for P7 (−2 AP; +0.5 ML and −1.2 DV); for P14 (−2.4 AP; +0.7 ML and −1.4 DV); for P21–Adult (−2.4 AP; +0.7 ML and −1.5 DV). For retrograde labeling from the cortex in P10 mice a total volume of 120 nl of CTB solution was injected at 4 nl s^−1^ in S1 of one hemisphere (coordinates −1.4 AP; +3 ML; −0.3 DV). We followed the same procedure for CTB injections at visual levels (coordinates −2.4 AP; +2.5 ML; +0.6 DV). After the specified period post injection (see below Confocal Imaging and Quantifications), mice were perfused with formalin and brains were fixed overnight in formalin at 4 °C. Brains were cryoprotected with 30% sucrose and 50 μm coronal sections were used for immunofluorescence and histological analyses. Additional detailed explanations of the coordinates and anatomical references can be found in the Supplementary Methods.

### In utero electroporation

Plasmids were pCAG-GFP, pCALNL-GFP (LoxP-Stop-LoxP-GFP; floxed-GFP), pCALNL-DsRed (LoxP-Stop-LoxP-DsREd; floxed DsRed), and pCAG-Cre (Addgene plasmids #11150, #13770, #13769, and #13775 gifts from Connie Cepko)^62^. CMV-synaptophysin-GFP plasmid was obtained from Addgene (plasmid #27235)^63^. For IUE^64^, timed pregnant mice were anesthetized with isoflurane/oxygen, and a solution containing a mixture of the specified plasmids at a concentration of 1 µg µl^−1^ each was injected into the embryo’s lateral ventricle using a 30 μm pulled glass micropipette. Five voltage pulses (38 mV, 50 ms) were applied using external paddles oriented in order to target S1, S2, or auditory cortex. After birth, pups were pups were anesthetized with carprofen (5 mg kg^−1^ bodyweight), or by induction of hypothermia, and fixed by intracardiac perfusion with ice-cold 4% paraformaldehy in PBS, pH 7.4, and at the specified developmental stages^39^. Reconstructions of single neurons were performed by electroporating pCALNL-GFP (1 µg µl^−1^) and a low concentration of pCAG-Cre (20 ng µl^−1^) to label neurons sparsely^64^.

### Whisker cauterization

P1 pups were anesthetized with ice and underwent complete cauterization of their mystacial whiskers and follicles on one or both sides of the face (Thermal Cautery Unit, Geiger Medical Technologies). No animal that underwent this procedure exhibited any sign of whisker regrowth throughout the course of the experiment. Animals were then returned to their mother and allowed to develop normally.

### Histology and immunohistochemistry

Fifty micrometers free-floating brain cryosections were used for immunohistochemistry or mounted for Nissl. For Nissl staining, mounted sections were dehydrated with ascending grades of alcohol and cleared with xylene using 0.1% Cresyl Violet (Sigma-Aldrich C5042) Acetate solution^65^. Primary antibodies used were rabbit anti-GFP (Thermo Fisher Scientific, Invitrogen A11122), rabbit anti-GFAP (Abcam, ab7260), mouse monoclonal anti-NeuN (Chemicon MAB377 clone A60), guinea pig anti-VGLUT2 (Merk Millipore AB2251-I), mouse monoclonal anti-Satb2 (Abcam, ab51502), rat monoclonal anti-Ctip2 (Abcam, ab18465), rabbit polyclonal anti-Cux1 (Santa Cruz Biotechnology, sc-13024), and mouse monoclonal anti human Rorb (Perseus Proteomics, PP-N7927-00); and secondary antibodies used were goat anti-rabbit-Alexa 488 (Life Technologies, catalog #A-11034), goat anti-guinea pig-Alexa 647 (Thermo Fisher Scientific, catalog #A21450), goat anti-rat Alexa 594 (Thermo Fisher Scientific, catalog #A-11007), and goat anti-rabbit Alexa 594 (Thermo Fisher Scientific, catalog #A-11037). Nuclei were stained with 4′,6-diamidino-2-phenylindole (DAPI) (D9542 Sigma).

### Confocal imaging and quantification

Confocal microscopy was performed with a TCS-SP5 or TCS-SP8 (Leica) Laser Scanning System on Leica DMI8 microscopes. Sections (50 µm) were obtained by taking 0.5 µm serial optical sections with LAS AF v1.8 software (Leica) and Tilescan images with Leica LAS AF software. Images of synaptophysin were acquired using a 1024 × 1024 scan format with a 63× objective. First, randomized red axons were identified in the region of interest and then the presence or absence of synaptophysin-GFP was identified.

Quantification of CTB^+^ cells was performed on single confocal images from z-stacks using DAPI and CTB staining. In each layer, the proportions of CTB^+^ cells were calculated among randomly selected cell nuclei in the defined cortical columns. Data are provided as the percentage of CTB^+^ cells out of selected DAPI^+^ cells. For quantifications of absolute numbers of CTB-labeled neurons, we counted the total number of CTB^+^ neurons per layer in the S1 region as delimited by the barrel anatomical landmarks. Data are provided as absolute numbers. To obtain refinement rates after the sequential injections of CTB coupled to two distinct fluorescence probes in the same mice, we calculated the fraction of cells labeled with the second probe among randomly selected neurons labeled with the probe used in the first injection, or vice versa. Quantifications were performed by three different observers. Quantifications were performed in the hemisphere contralateral to the site of injection. This ensures that all neurons that are labeled with CTB possess at the moment of injection at least one interhemispheric projection in the contralateral territory, regardless of the growth or injury status of these projections. To facilitate the equivalent quantification of CTB-labeled cells at different developmental stages, quantifications were performed at P10 following injections at P3, P5, and P7, and 2 days post surgery following injections at P14, P21, and P30. This allowed proper cortical lamination and avoids potential interference from early proliferation, since proliferation in the cortical layers is minor between P10 and P32^66^. It also avoids effects from incomplete neuronal migration of pyramidal and interneuron populations, or from early waves of cell death. To identify the locations of functional areas and regions in the adult we used the atlas of Paxinos^67^. To localize and delimit the equivalent areas in the developing brain, we used the Developing Brain from Allen (http://atlas.brain-map.org/). Additional coordinates for cortical areas can be found in the Supplementary Methods.

For acquisition and quantifications of fluorescence signal^39,64^, detectors were set to ensure linearity and equivalent conditions between all samples by determining the minimum and maximum threshold signals. The threshold for background noise was determined using regions outside of electroporated areas^39,64,68^. The maximum threshold signal was set by ensuring that no pixels were saturated. This avoids an artifactual increase in low signals in order to ensure linearity. Quantifications were performed by converting pixel intensity into binary values using Fiji^69^. Contralateral fluorescence values are given relative to the values of fluorescence of the ipsilateral electroporated areas that were outlined manually and normalized to control conditions.

### Electrophysiological recordings in vivo

Adult mice (2–5 months) were anesthetized by isoflurane and placed into a stereotaxic frame (0.2–0.5% in oxygen as necessary). Ophthalmic ointment was used to protect the animal’s eyes during the surgery and replaced with silicon oil during recordings. Body temperature was kept at 37 °C by a controlled heating blanket. Surgery and insertion of the recording and stimulating electrodes were made under a surgical lens. After resection of the scalp, a bilateral craniotomy of ~1 mm diameter for recording and stimulation was performed above the visual cortex. A multichannel linear silicon probe (A1x16-5mm-100-703; NeuroNexus) was placed at an angle of 30° relative to the coronal plane to target the V1/V2 area^70^ (stereotaxic coordinates were AP: −2.88 to −2.98; ML: 2.95); and the tungsten stimulating electrode (0.5 MΩ) was placed in V1 (AP: –2.88 to 2.98; ML: 2.5; at 300 ± 50 µm depth). A subcutaneous silvered wire was placed in the neck as a reference electrode. The linear array of the multielectrode covered the entire cortical column (~800 µm). Impedances were checked for each channel separately and found to be in the range of 0.32–0.47 kΩ. The electrodes were slowly inserted into the tissue. Recordings were acquired ~20 min after the desired electrode position was acquired. The signals were pre-amplified, digitized at 10 kHz, recorded and stored with an AC amplifier (Multichannel Systems). Evoked responses were obtained for a range of stimulation intensities (0.2–1.2 mA), and peak amplitude and area of local field potentials (LFPs) were measured. One-dimensional CSD signals were calculated from the second spatial derivative of LFP (100 µm resolution). Smoothing was only applied to CSD signals for visualization purposes. Tissue conductivity was considered isotropic. Stripes in the background CSD due to impedance and offset differences between sites were clearly separated from the relevant CSD responses. CSD mean values of layer 4 (50 ms range) were calculated 60 ms after stimulus. Data analysis was performed using routines written in MATLAB 10 (MathWorks). Arbitrary values from −1 to 1 were assigned to express CSD signals as µV mm^−2^ in the heat map.

### Statistical analysis

There was no specific statistical method to determine sample size. Sample size was determined to be adequate based on the magnitude and consistency of measurable differences between groups. In principle, data were only excluded for failed injections. Experiments were reliably reproduced. Each experimental condition was carried out with a minimum of three biological replicates, a minimum of two coronal sections from each brain, and a minimum total number of 300 cells. Investigators were not blinded to mouse genotypes during experiments. Results were reliably reproduced by three different observers. Results are expressed as the mean ± standard error of the mean. Normality was tested using D’Agostino and Pearson omnibus normality test. Results were compared using three-way analysis of variance (ANOVA), two-way ANOVA, one-Way ANOVA with post hoc comparison with Tukey’s or Sidak’s test, a *χ*^2^ test, or a two-tailed Student’s unpaired *t* test as indicated in the corresponding figures. *F* values and degrees of freedom (DFn, DFd) of ANOVA analyses are provided in figure legends. Groups were assigned based on area, treatment (whisker depletion), or genotypes.

### Reporting summary

Further information on research design is available in the Nature Research Reporting Summary linked to this article.

## Supplementary information

Supplementary Information

Peer Review File

Reporting Summary

Source Data

## Data Availability

The authors declare that the data supporting the findings of this study are available within the paper and its Supplementary Information files. The source data underlying main Figs. 1–7, and Supplementary Figs. 1–11, are provided as a Source Data file and also available at: https://osf.io/m5sby/.

## References

[1] Hill RS, Walsh CA (2005). Molecular insights into human brain evolution. Nature.

[2] Blockus H, Polleux F (2018). Circuit wiring: neurite speed dating versus stable synaptic matchmaking. Dev. Cell.

[3] Krageloh-Mann I, Lidzba K, Pavlova MA, Wilke M, Staudt M (2017). Plasticity during early brain development is determined by ontogenetic potential. Neuropediatrics.

[4] Lokmane L, Garel S (2014). Map transfer from the thalamus to the neocortex: inputs from the barrel field. Semin. Cell Dev. Biol..

[5] Frangeul L (2016). A cross-modal genetic framework for the development and plasticity of sensory pathways. Nature.

[6] Jung WB (2016). Neuroplasticity for spontaneous functional recovery after neonatal hypoxic ischemic brain injury in rats observed by functional MRI and diffusion tensor imaging. Neuroimage.

[7] Lodato S, Shetty AS, Arlotta P (2015). Cerebral cortex assembly: generating and reprogramming projection neuron diversity. Trends Neurosci..

[8] Doi H, Shinohara K (2017). fNIRS studies on hemispheric asymmetry in atypical neural function in developmental disorders. Front Hum. Neurosci..

[9] Fenlon LR, Richards LJ (2015). Contralateral targeting of the corpus callosum in normal and pathological brain function. Trends Neurosci..

[10] Fame RM, MacDonald JL, Macklis JD (2011). Development, specification, and diversity of callosal projection neurons. Trends Neurosci..

[11] Lodato S, Arlotta P (2015). Generating neuronal diversity in the mammalian cerebral cortex. Annu. Rev. Cell Dev. Biol..

[12] Suarez R, Gobius I, Richards LJ (2014). Evolution and development of interhemispheric connections in the vertebrate forebrain. Front. Hum. Neurosci..

[13] Innocenti GM, Fiore L, Caminiti R (1977). Exuberant projection into the corpus callosum from the visual cortex of newborn cats. Neurosci. Lett..

[14] O’Leary DD, Stanfield BB, Cowan WM (1981). Evidence that the early postnatal restriction of the cells of origin of the callosal projection is due to the elimination of axonal collaterals rather than to the death of neurons. Brain Res..

[15] Innocenti GM, Clarke S (1983). Multiple sets of visual cortical neurons projecting transitorily through the corpus callosum. Neurosci. Lett..

[16] Meissirel C, Dehay C, Berland M, Kennedy H (1991). Segregation of callosal and association pathways during development in the visual cortex of the primate. J. Neurosci..

[17] Dehay C, Kennedy H, Bullier J (1986). Callosal connectivity of areas V1 and V2 in the newborn monkey. J. Comp. Neurol..

[18] Innocenti GM, Clarke S (1984). The organization of immature callosal connections. J. Comp. Neurol..

[19] Clarke S, Innocenti GM (1986). Organization of immature intrahemispheric connections. J. Comp. Neurol..

[20] O’Leary DD, Koester SE (1993). Development of projection neuron types, axon pathways, and patterned connections of the mammalian cortex. Neuron.

[21] O’Leary DD (1987). Remodelling of early axonal projections through the selective elimination of neurons and long axon collaterals. Ciba Found. Symp..

[22] Stanfield BB, O’Leary DD, Fricks C (1982). Selective collateral elimination in early postnatal development restricts cortical distribution of rat pyramidal tract neurones. Nature.

[23] Innocenti GM, Clarke S, Koppel H (1983). Transitory macrophages in the white matter of the developing visual cortex. II. Development and relations with axonal pathways. Brain Res..

[24] Molyneaux BJ (2009). Novel subtype-specific genes identify distinct subpopulations of callosal projection neurons. J. Neurosci..

[25] Koester SE, O’Leary DD (1993). Connectional distinction between callosal and subcortically projecting cortical neurons is determined prior to axon extension. Dev. Biol..

[26] Custo Greig LF, Woodworth MB, Galazo MJ, Padmanabhan H, Macklis JD (2013). Molecular logic of neocortical projection neuron specification, development and diversity. Nat. Rev. Neurosci..

[27] Fame RM, Dehay C, Kennedy H, Macklis JD (2017). Subtype-specific genes that characterize subpopulations of callosal projection neurons in mouse identify molecularly homologous populations in Macaque cortex. Cereb. Cortex.

[28] Molyneaux BJ (2015). DeCoN: genome-wide analysis of in vivo transcriptional dynamics during pyramidal neuron fate selection in neocortex. Neuron.

[29] Azim E, Shnider SJ, Cederquist GY, Sohur US, Macklis JD (2009). Lmo4 and Clim1 progressively delineate cortical projection neuron subtypes during development. Cereb. Cortex.

[30] Pouchelon G (2014). Modality-specific thalamocortical inputs instruct the identity of postsynaptic L4 neurons. Nature.

[31] Telley L (2016). Sequential transcriptional waves direct the differentiation of newborn neurons in the mouse neocortex. Science.

[32] Nowakowski TJ (2017). Spatiotemporal gene expression trajectories reveal developmental hierarchies of the human cortex. Science.

[33] Telley L, Jabaudon D (2018). A mixed model of neuronal diversity. Nature.

[34] Mancinelli S, Lodato S (2018). Decoding neuronal diversity in the developing cerebral cortex: from single cells to functional networks. Curr. Opin. Neurobiol..

[35] Harb K (2016). Area-specific development of distinct projection neuron subclasses is regulated by postnatal epigenetic modifications. Elife.

[36] Keller D, Ero C, Markram H (2018). Cell densities in the mouse brain: a systematic review. Front Neuroanat..

[37] Schubert D, Kotter R, Staiger JF (2007). Mapping functional connectivity in barrel-related columns reveals layer- and cell type-specific microcircuits. Brain Struct. Funct..

[38] Harris JA (2014). Anatomical characterization of Cre driver mice for neural circuit mapping and manipulation. Front. Neural Circuits.

[39] Rodriguez-Tornos FM (2016). Cux1 enables interhemispheric connections of layer II/III neurons by regulating Kv1-dependent firing. Neuron.

[40] Suarez R (2014). Balanced interhemispheric cortical activity is required for correct targeting of the corpus callosum. Neuron.

[41] Bosman LW (2011). Anatomical pathways involved in generating and sensing rhythmic whisker movements. Front. Integr. Neurosci..

[42] Goldin MA, Harrell ER, Estebanez L, Shulz DE (2018). Rich spatio-temporal stimulus dynamics unveil sensory specialization in cortical area S2. Nat. Commun..

[43] Smith PH, Uhlrich DJ, Manning KA, Banks MI (2012). Thalamocortical projections to rat auditory cortex from the ventral and dorsal divisions of the medial geniculate nucleus. J. Comp. Neurol..

[44] Li H (2013). Laminar and columnar development of barrel cortex relies on thalamocortical neurotransmission. Neuron.

[45] Narboux-Neme N, Pavone LM, Avallone L, Zhuang X, Gaspar P (2008). Serotonin transporter transgenic (SERTcre) mouse line reveals developmental targets of serotonin specific reuptake inhibitors (SSRIs). Neuropharmacology.

[46] Anton-Bolanos N, Espinosa A, Lopez-Bendito G (2018). Developmental interactions between thalamus and cortex: a true love reciprocal story. Curr. Opin. Neurobiol..

[47] Rouaux C, Arlotta P (2013). Direct lineage reprogramming of post-mitotic callosal neurons into corticofugal neurons in vivo. Nat. Cell Biol..

[48] De la Rossa A (2013). In vivo reprogramming of circuit connectivity in postmitotic neocortical neurons. Nat. Neurosci..

[49] Srinivasan K (2012). A network of genetic repression and derepression specifies projection fates in the developing neocortex. Proc. Natl. Acad. Sci. USA.

[50] Paolino A, Fenlon LR, Suarez R, Richards LJ (2018). Transcriptional control of long-range cortical projections. Curr. Opin. Neurobiol..

[51] Zhou J (2013). Axon position within the corpus callosum determines contralateral cortical projection. Proc. Natl. Acad. Sci. USA.

[52] Stricker SH, Gotz M (2018). DNA-methylation: master or slave of neural fate decisions?. Front. Neurosci..

[53] Oishi K, Aramaki M, Nakajima K (2016). Mutually repressive interaction between Brn1/2 and Rorb contributes to the establishment of neocortical layer 2/3 and layer 4. Proc. Natl. Acad. Sci. USA.

[54] Hou PS, Miyoshi G, Hanashima C (2019). Sensory cortex wiring requires preselection of short- and long-range projection neurons through an Egr-Foxg1-COUP-TFI network. Nat. Commun..

[55] De Rubeis S (2014). Synaptic, transcriptional and chromatin genes disrupted in autism. Nature.

[56] Parikshak NN, Gandal MJ, Geschwind DH (2015). Systems biology and gene networks in neurodevelopmental and neurodegenerative disorders. Nat. Rev. Genet..

[57] Huang Y (2013). Sensory input is required for callosal axon targeting in the somatosensory cortex. Mol. Brain.

[58] Ten Tusscher MPM, Houtman AC, De Mey J, Van Schuerbeek P (2018). Cortical visual connections via the corpus callosum are asymmetrical in human infantile esotropia. Strabismus.

[59] Dayer A (2014). Serotonin-related pathways and developmental plasticity: relevance for psychiatric disorders. Dialogues Clin. Neurosci..

[60] Lesch KP, Waider J (2012). Serotonin in the modulation of neural plasticity and networks: implications for neurodevelopmental disorders. Neuron.

[61] Brockschnieder D, Pechmann Y, Sonnenberg-Riethmacher E, Riethmacher D (2006). An improved mouse line for Cre-induced cell ablation due to diphtheria toxin A, expressed from the Rosa26 locus. Genesis.

[62] Matsuda T, Cepko CL (2007). Controlled expression of transgenes introduced by in vivo electroporation. Proc. Natl. Acad. Sci. USA.

[63] Dittgen T (2004). Lentivirus-based genetic manipulations of cortical neurons and their optical and electrophysiological monitoring in vivo. Proc. Natl. Acad. Sci. USA.

[64] Briz, C. G., Navarrete, M., Esteban, J. A. & Nieto M. In utero electroporation approaches to study the excitability of neuronal subpopulations and single-cell connectivity. *J. Vis. Exp.*10.3791/55139 (2017). 10.3791/55139 PMC5409320 28287556

[65] Cubelos B (2008). Cux-2 controls the proliferation of neuronal intermediate precursors of the cortical subventricular zone. Cereb. Cortex.

[66] Tabata H (2015). Diverse subtypes of astrocytes and their development during corticogenesis. Front. Neurosci..

[67] Paxinos, G., Franklin, K. B. J. *The Mouse Brain in Stereotaxic Coordinates: Compact* 2nd edn (Elsevier Academic Press, 2004).

[68] Plas DT, Visel A, Gonzalez E, She WC, Crair MC (2004). Adenylate cyclase 1 dependent refinement of retinotopic maps in the mouse. Vis. Res..

[69] Schindelin J (2012). Fiji: an open-source platform for biological-image analysis. Nat. Methods.

[70] Mizuno H, Hirano T, Tagawa Y (2007). Evidence for activity-dependent cortical wiring: formation of interhemispheric connections in neonatal mouse visual cortex requires projection neuron activity. J. Neurosci..

