# Supplementary material for: Transient callosal projections of L4 neurons are eliminated for the acquisition of local connectivity
Source: Nat Commun. 2019 Oct 7;10:4549. doi: 10.1038/s41467-019-12495-w (PMC6779895; doi:10.1038/s41467-019-12495-w)
Supplement: Supplementary file 1 — Supplementary Information [file 41467_2019_12495_MOESM1_ESM.pdf]

Supplementary Information

Transient callosal projections of L4 neurons are eliminated for the acquisition of local connectivity

De León Reyes et al.,

Supplementary figures

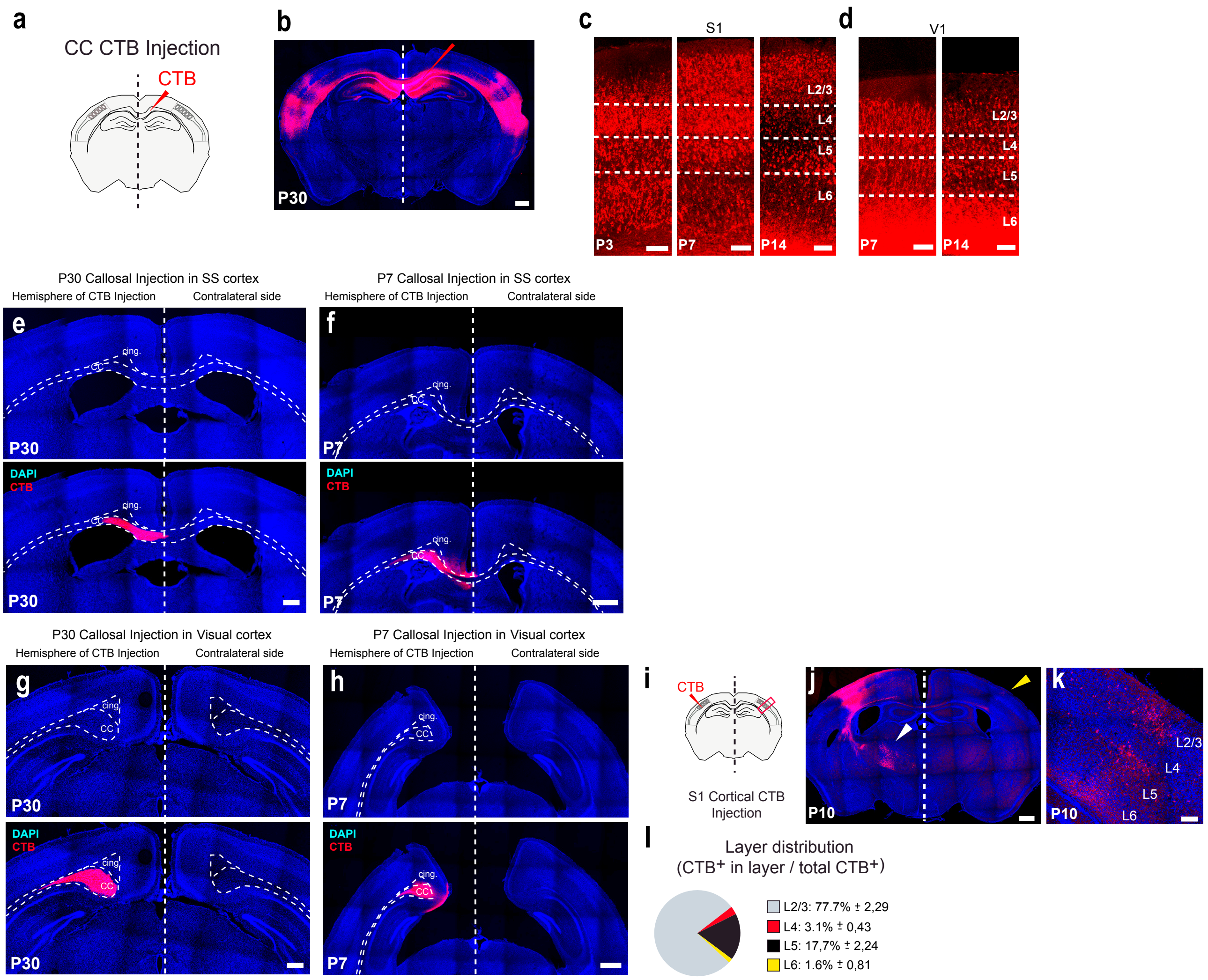

Supplementary Figure 1: CTB injections for in vivo detection of inter-hemispheric projections.
a Schematic representation of CTB-555 injection in the CC. **b** Coronal section of a mouse brain 48h after callosal injection of CTB-555 at P30. CTB (red), DAPI (blue). Red arrows indicate site of injection in (a) and (b). **c** Detail of S1 cortex following CTB-555 (red) injections in the CC performed at P3, P7 or P14. **d** Detail of V1 cortex after CTB-555 injections in the CC performed at P7 or P14. Both S1 and V1 show a gradual loss over time in the number of CTB^{+} cells. **e-h** Coronal sections of mouse brains injected with stage-specific volumes (see Methods and Supplementary text) of CTB-555 in the SS cortex (e-f) or visual cortex (g-h) and perfused within two hours after injections demonstrate equivalent spreading in P30 (e, g) and P7 (f, h). Spreading of CTB completely fills and is restricted to the CC, and does not enter other brain regions or axonal tracks such as the cingulum bundle (cing). **i** Schematic representation of CTB injections in the cortical plate. The red rectangle indicates the location of contralateral homotopic labeled S1 neurons. **j** Coronal section of a P10 brain injected in the cortical plate in S1 with CTB-555. Retrograde labeling is observed in ipsilateral somatosensory thalamic nuclei as indicated by the white arrow, and in contralateral homotopic S1, as indicated by the yellow arrow. **k** Magnification of contralateral S1 after injections in (i). CTB^{+} neurons in the contralateral homotopic S1 are mainly located in L2/3 and L5. **l** Quantification of the distribution of total CTB^{+} neurons identified in homotopic contralateral S1 after S1 CTB cortical injections at P10. (n=300 CTB^{+} neurons, n= 6 sections, n= 3 mice). Data shows mean ± SEM. Scale bars represent 500 µm (b, e, f, g, h, j) and 100 µm (c, d, k). Source data are provided as a Source Data file.

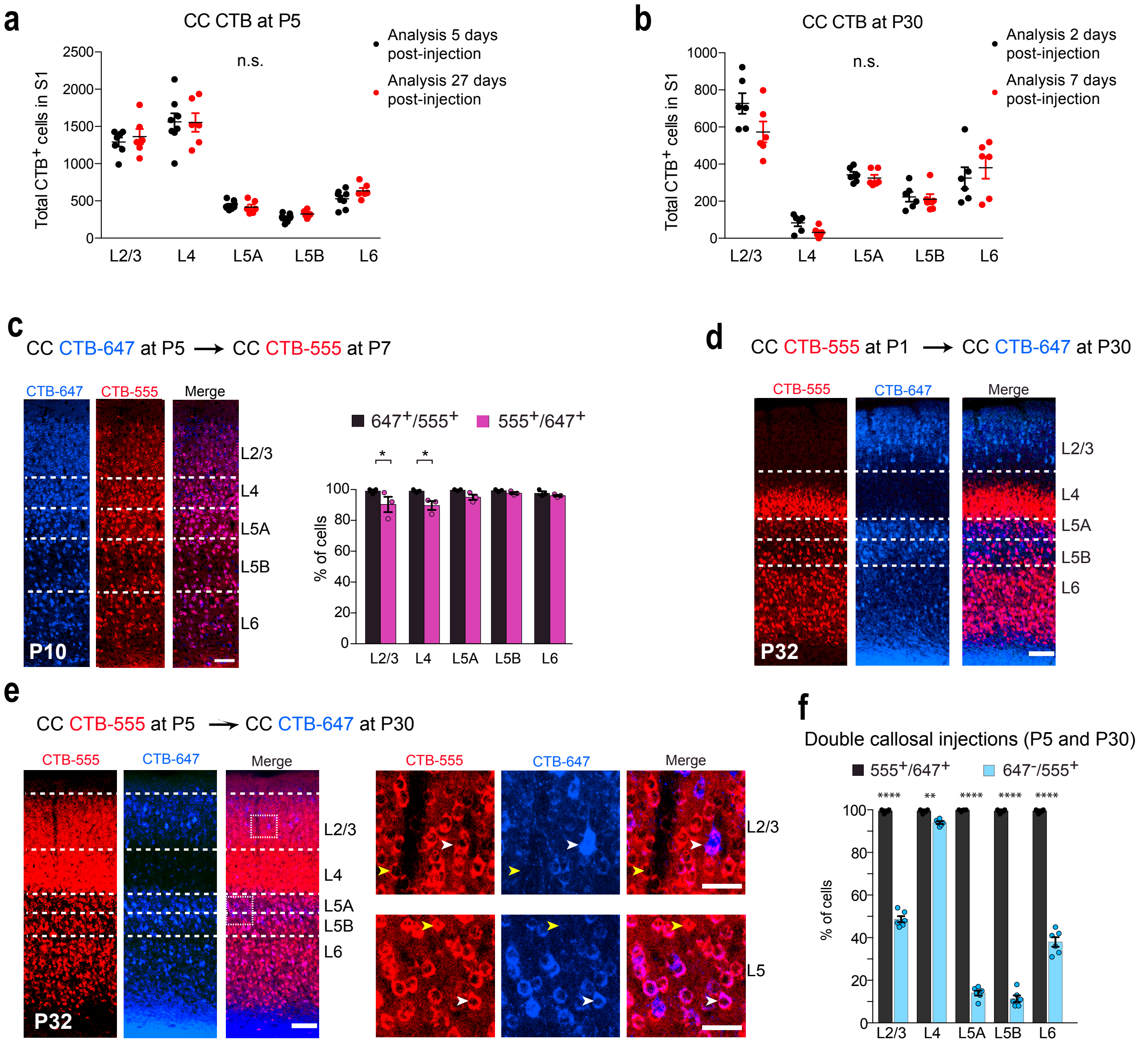

Supplementary Figure 2: Saturating and specificity of CTB injections.
a Absolute numbers of CTB$^+$ neurons in S1 quantified after injections at P5 and brain collection 5 or 27 days post-injection. p-value CTB+/day of analysis= non-significant (Two-Way ANOVA (n= 6): F CTB$^+$/day of analysis (1, 59) = 0.377). **b** Absolute numbers of CTB$^+$ neurons in S1 quantified after injections at P30 and brains collection 2 or 7 days post-injection.) (n=3 mice per condition, n= 2 sections per animal) (a, b). p-value CTB$^+$/day of analysis= non-significant (Two-Way ANOVA (n= 6): F CTB$^+$/day of analysis (1, 50) = 1.874). **c-e** Injections of CTB-647 (blue) and CTB-555 (red) performed in the same animal at two developmental stages. **c** Detail of S1 cortex after sequential injections at P5 and P7 and brains collected at P10. Graph ahos the fraction of 555$^+$ P7 labeled cells that were also labeled with 647$^+$ at P5 (647$^+$/555$^+$) and of cells labeled at P5 with 647$^+$ also positive for 555$^+$ (555$^+$/647$^+$) (n= 300 neurons, n= 3 mice per stage). p-value * ≤0.05 (Student's t-test). **d** Detail of S1 cortex following injections at P1 (CTB-555; red) and at P30 (CTB-647; blue). **e** S1 following injections at P5 (CTB-555; red) and at P30 (CTB-647; blue). Magnifications of outlined regions are shown in right panels. White arrows: examples of cells labeled with CTB-647 (blue) also labeled with CTB-555 (red). Yellow arrows: cells labeled with CTB-555 (red) negative for CTB-647 (blue). **f** Quantifications after injections as in (e). Dark columns: fractions of CTB-647 labeled cells positive for CTB-555 (555$^+$/647$^+$). Blue columns: fractions of CTB-555-labeled cells negative for CTB-647 (647$^-$/555$^+$; single labeled) representing refinement rates. (n= 1000 neurons per layer, n= 2 section per mice, n= 3 mice). p-value double labeled/single labeled ≤ 0.0001; p-value single labeled/layer ≤0.0001 (Two-Way ANOVA (n= 3): F double labeled/single labeled (1, 50) = 7269; F single labeled/layer (4, 25) = 499.6); p-value vs double labeled **≤ 0.01 ****≤ 0.0001 (Post-hoc comparison with Sidak's test). Scale bars represent 100 µm in (c-e) and 20 µm in right panels in (e). Data shows mean ± SEM (error bars). Source data are provided as a Source Data file.

De León-Reyes et al., Figure S2

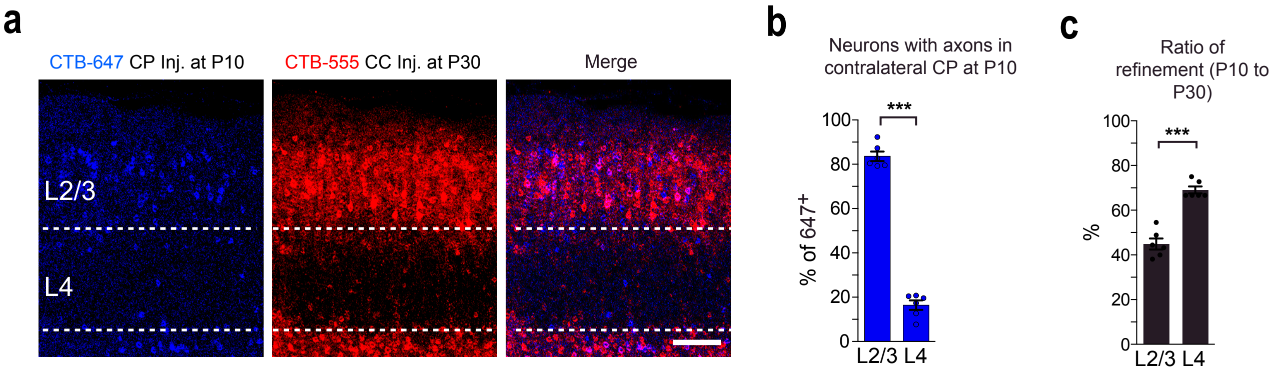

Supplementary Figure 3: Dynamics of L4 callosal projections in S1.

a Images showing upper layers of the cortex labeled with CTB by injections performed sequentially in the contralateral cortical plate at P10 (CTB-647, blue) and in the CC at P30 (CTB-555, red) in the same mice. Cells labeled only in blue indicate refinement, as they were labeled at P10 but not labeled by injections in the CC at P30 (single labeled: 647^{+}; 555^{-}). Cells in blue and red indicate neurons that have integrated into the final circuit and whose callosal axons were detected in the cortical plate already at P10 (neurons which stabilize their callosals). **b** Layer distribution of cells labeled from injections in the cortical plate at P10 reveals that some L4 neurons can be detected invading the CP. **c** Refinement rate in the P10-P30 window (calculated as the fraction of CTB-647 single labeled neurons out of the total number of CTB-647^{+} neurons) is greater in L4 than in L2/3 neurons. (n= 300 neurons, n= 6 sections, n= 3 mice per condition). Data shows mean ± SEM (error bars). p-value: ***≤ 0.001 (Student's t-test). Scale bar represents 100 µm. Source data are provided as a Source Data file.

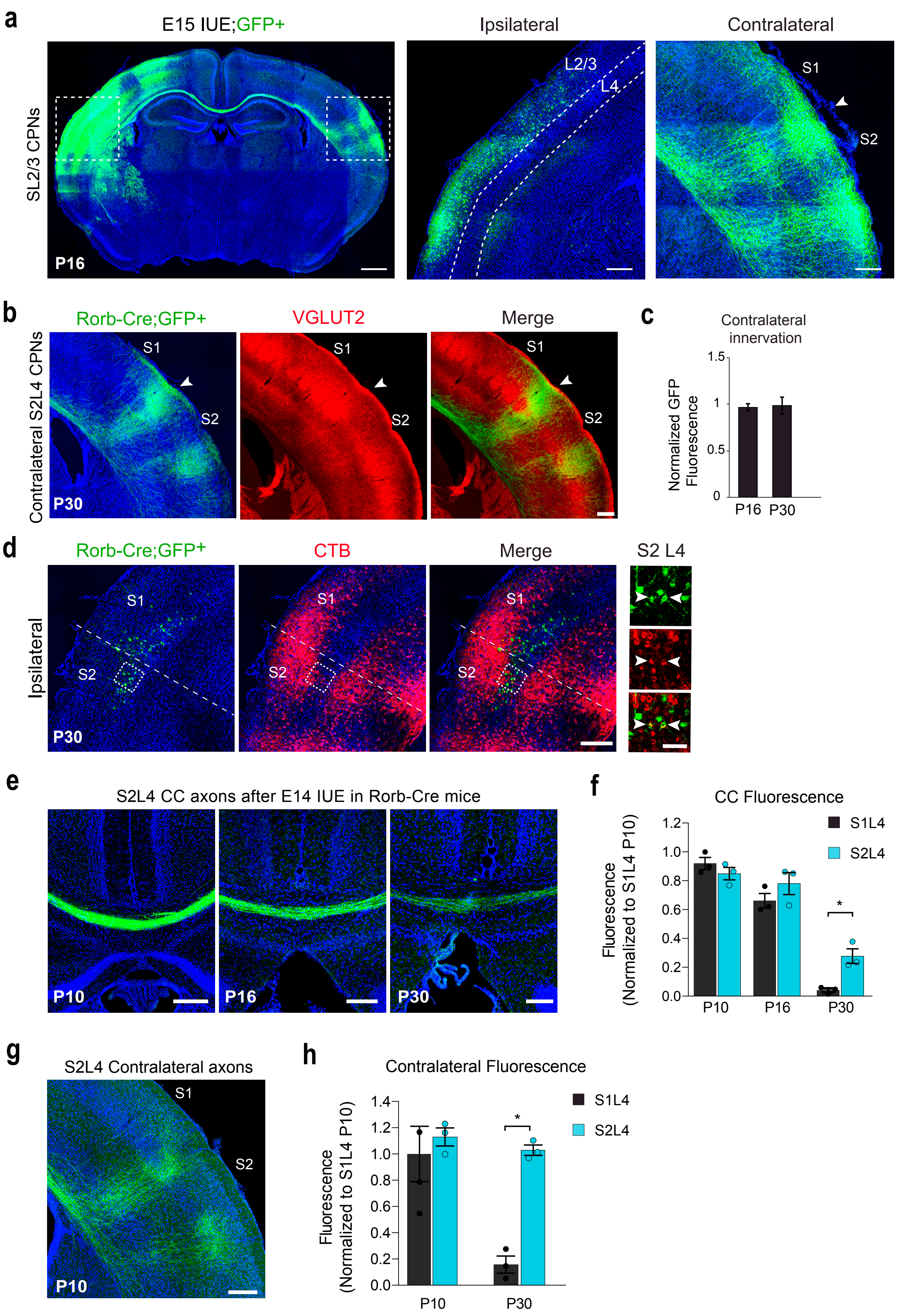

De León-Reyes et al., Figure S4

Supplementary Figure 4: Comparison of SL2/3 and S2L4 callosal projections.

a Electroporation of WT embryos labeling SL2/3 (S1 plus S2) shows contralateral columns at the border of S1/S2 (white arrowhead in the right panel) and in S2 in P30 brains. Boxes indicate magnified ipsilateral (center) and contralateral (right) areas. **b** Contralateral innervation of S2L4 callosals in a P30 Rorb-Cre mouse electroporated with floxed-GFP showing contralateral columns in S1/S2 border (white arrowhead) and S2. VGlut2 labeling (red) shows barrels. **c** Quantification of S2L4 contralateral axonal invasion normalized to the ipsilateral signals at P16 and P30. (n= 9 sections, n= 3 mice per condition). **d** Coronal section from a Rorb-Cre mouse brain electroporated with floxed-GFP at E14 and injected with CTB-555 in the CC at P30 corresponding to the insets shown in main Fig. 5j. Dashed line indicates S1-S2 border. In the right, magnification of outlined region in S2 as in Fig. 5j shows co-labeled cells. Scale bars represent 500 µm (a, left panel) and 200 µm (a, middle and right panels, b and d). e-h Dynamics of refinement of S2L4 axons. **e** Images of the CC in animals electroporated in S2 analyzed at P10, P16 and P30. **f** Quantification of refinement dynamics showing the ratio of S1L4 and S2L4 GFP axons in the CC to ipsilateral GFP neurons normalized to the mean value of S1L4 at P10. (n= 18 mice, 3 per condition). Mean ± SEM (error bars). p-value S1L4/S2L4 *$\leq$0.05 (Two-Way ANOVA (n= 18): F S1L4/S2L4 value (1, 12) = 5.40). p-value vs. S1 *$\leq$0.05 (Post-hoc with Tukey test). **g** Contralateral axons of S2L4 Rorb-Cre electroporated neurons at P10. **h** Quantification of refinement dynamics showing the ratio of S1L4 and S2L4 contralateral axons to ipsilateral GFP neurons normalized to the mean value of S1L4 at P10. Mean ± SEM (error bars) (n= 3 mice per condition). p-value S1L4/S2L4 *$\leq$0.01 (Two-Way ANOVA (n= 12). F S1L4/S2L4 (1, 9) = 12.13). p-value vs. S1 *$\leq$0.05 (Post-hoc with Tukey test). Scale bars represent 200 µm (e and g). Data shows mean ± SEM. Source data are provided as a Source Data file.

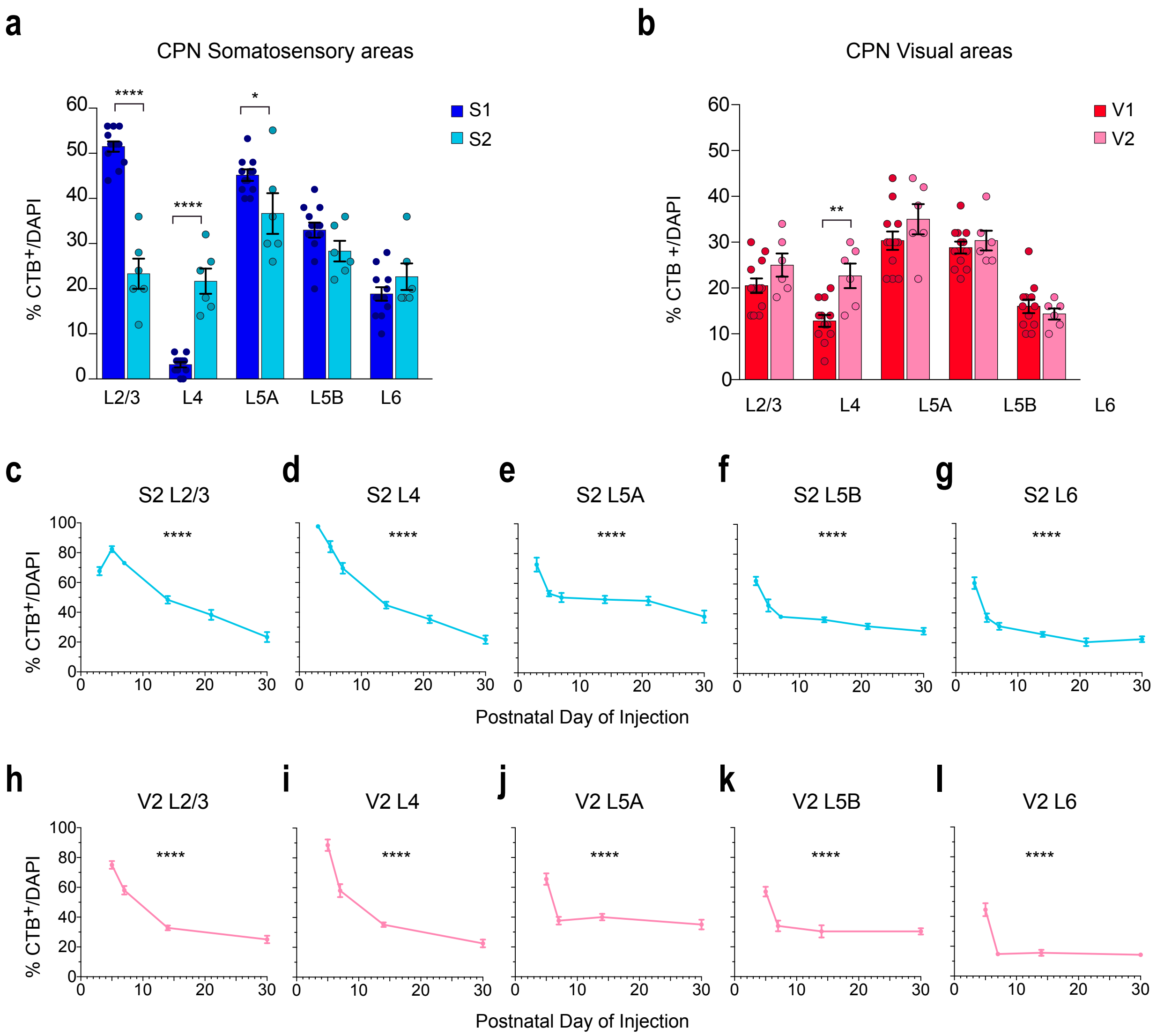

Supplementary Figure 5: Dynamics of refinement in S2 and V1 cortex.

a Comparison of the distributions of CPN in S1 versus S2 of adult brains as measured by CTB labeling upon injection in the CC at P30. p-value S1/S2 ≤0.0001. (Two-Way ANOVA factor interaction: F S1/S2 (1, 80) = 8.625). p-value vs. S1 ****≤0.0001, *≤0.05 (Post-hoc with Tukey test). **b** Comparison of the distributions of CPN in different cortical layers of adult V1 versus V2 as measured by CTB labeling upon injection in the CC at P30. p-value V1/V2 ≤0.01. (Two-Way ANOVA: F V1/V2 (1, 80) = 9.331). p-value vs. V1 **≤0.01 (Post-hoc with Tukey test). **c-l** Changes in the distribution of CTB^{+} CPN neurons in S2 (c-g) and V2 (h-l) during development (n=300 CTB^{+} neurons, n= 6 sections n=3 mice per developmental stage per stage. Data shows mean ± SEM (error bars). (c-l) ****p-value ≤0.0001 (Two-Way ANOVA (n= 18): F CTB^{+} over DAPI/time (4, 21)= 78.03 (c); F CTB+ over DAPI/time (4, 21)= 142.7 (d); F CTB^{+} over DAPI/time (4, 21)= 13,76 (e); F CTB^{+} over DAPI/time (4, 21)= 23.94 (f); F CTB^{+} over DAPI/time (4, 21)= 26.31 (g); F CTB^{+} over DAPI/time (3, 16) = 47.22 (h); F CTB+ over DAPI/time (3, 16)= 64.79 (i); F CTB^{+} over DAPI/time (3, 16)= 30.25 (j); F CTB^{+} over DAPI/time (3, 16) = 29.57 (k); F CTB^{+} over DAPI/time (3, 16) = 40.08 (l). Source data are provided as a Source Data file.

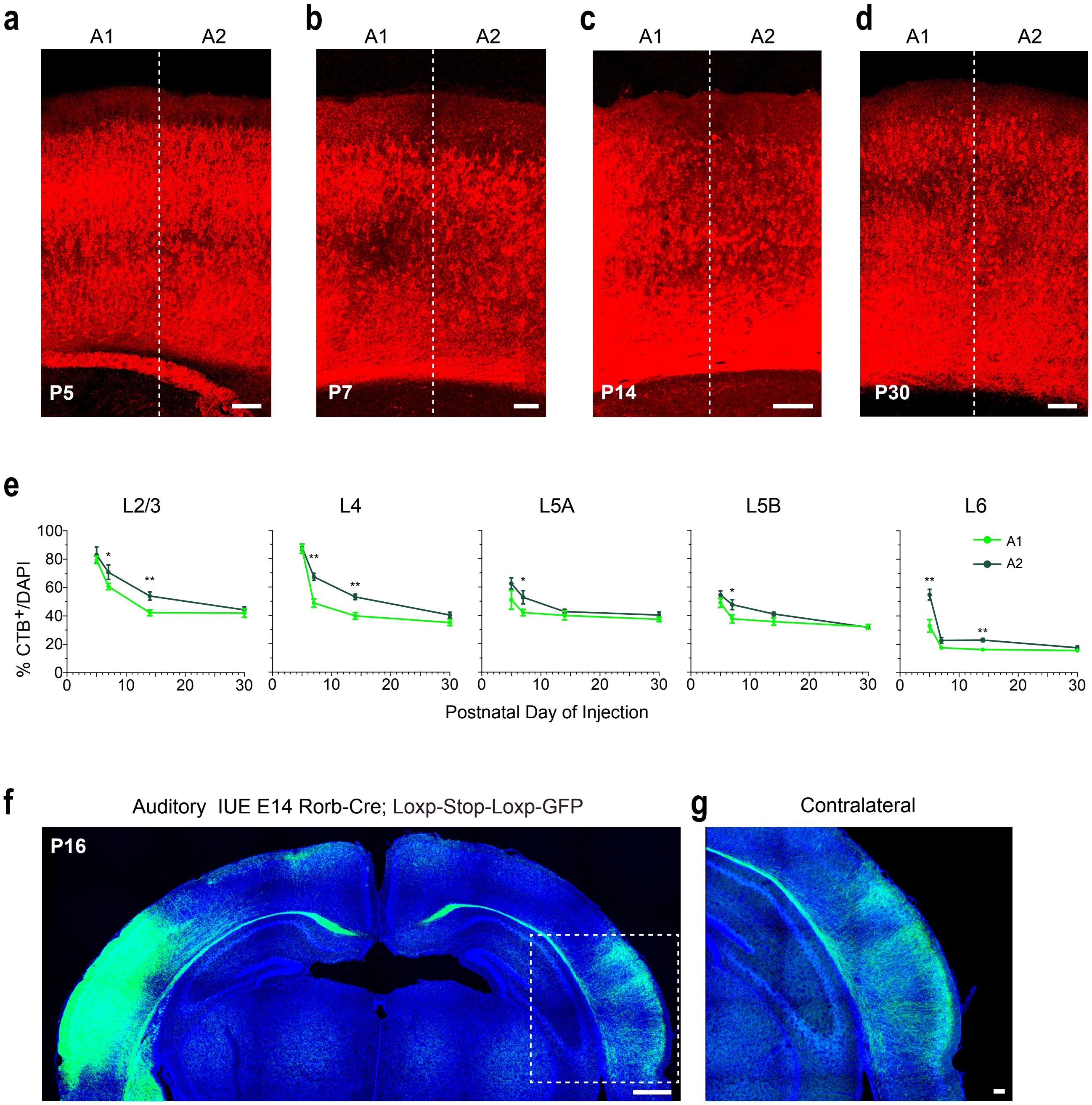

Supplementary Figure 6: Inter-hemispheric connectivity in A1.

a-d CTB-555 labeling in the auditory cortex after callosal injections at P5, P7, P14 and P30. A1 refers to primary auditory cortex, and A2 to secondary auditory cortex. **e** Developmental dynamics of refinement of callosal projections of neurons in A1 and A2 areas (n= 300 CTB+ neurons, n= 2 sections per mice, n= 12 mice in A1 and n= 12 mice in A2). Data shows mean ± SEM (error bars). p-value CTB/time ≤0.0001 for all layers (Two-Way ANOVA(n= 24): F CTB/time L2/3 (3, 34)= 53.52; F CTB/time L4 (3, 34)= 122.2; F CTB/time L5A (3, 34)= 9.841; F CTB/time L5B (3, 34)= 21.83; F CTB/time L6 (3, 34)= 66.97. p-value A1 vs A2 *≤0.05, **≤0.01 (Post-hoc with Tukey test). **f** Coronal brain section from a P16 Rorb-Cre mouse electroporated in the auditory cortex at E14 with floxed-GFP. **g** Contralateral auditory cortex from (f). Scale bars represent 500 µm (f) and 100 µm (a-d, g). Source data are provided as a Source Data file.

De León-Reyes et al., Figure S6

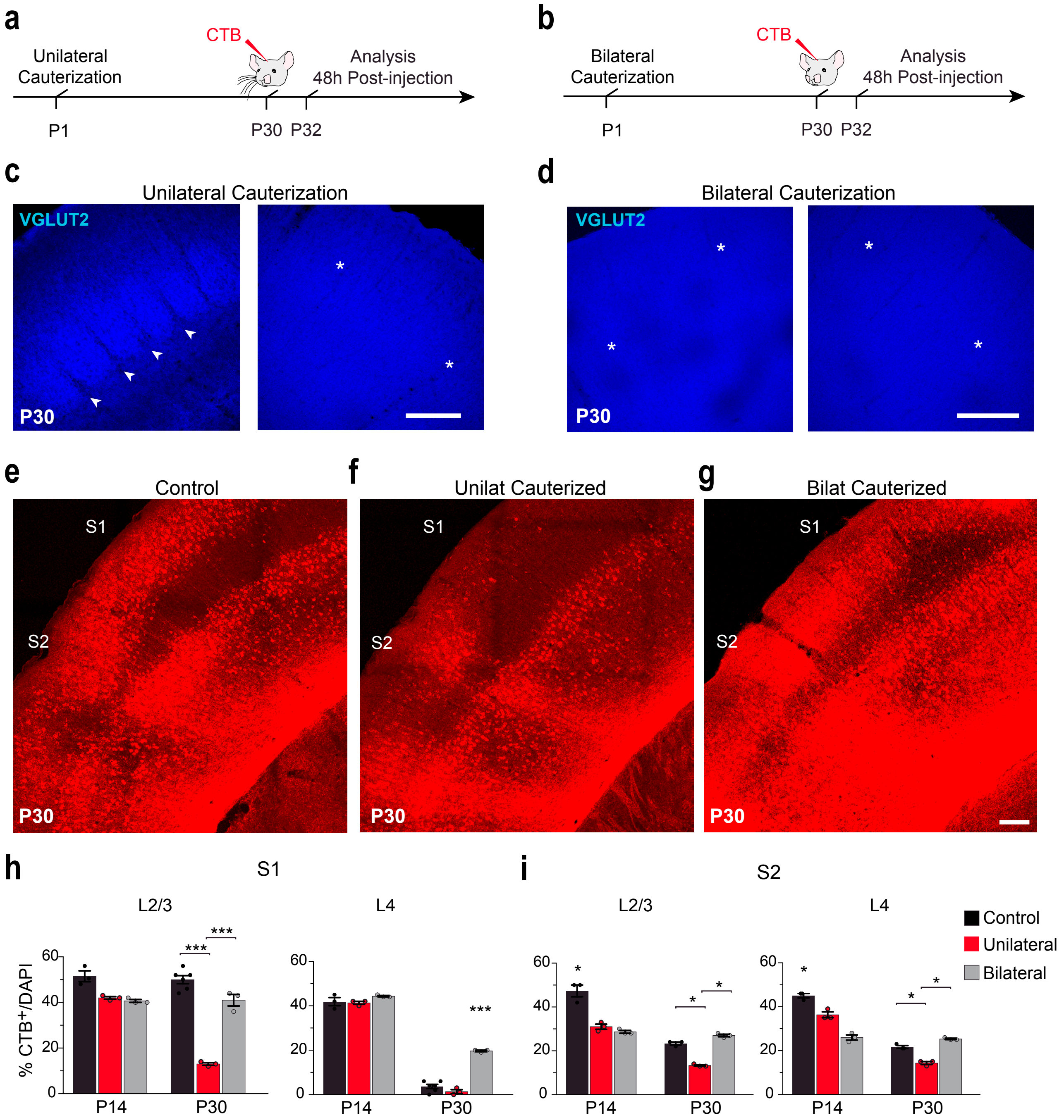

Supplementary Figure 7: Effect of whisker derived activity in callosal refinement.
a, b Schemes of experimental procedures to analyze the effect of early unilateral (a) and bilateral (b) whisker deprivation generating unbalanced (unilateral) and balanced (bilateral) activity. Injections of CTB-555 in the CC were performed at P30 after cauterization at P1. Quantification of CPN was performed 48h post-injection. **c** VGlut2 staining in both brain hemispheres of unilaterally cauterized mice. Individual barrels are demarcated by white arrows and are lost in the hemisphere contralateral to cauterization. Stars indicate the limits of S1. **d** VGlut2 staining in both brain hemispheres of bilaterally cauterized mice. Barrels are lost in both hemispheres. Stars indicate the limits of S1. **e-g** Coronal sections of P30-injected brains showing CTB$^+$ cells in S1 and S2 in control (e), unilaterally- (f), and bilaterally- (g) cauterized mice. **h-i** Quantifications of CTB$^+$ cells in L2/3 and L4 in S1 and S2 at P14 and P30 in control, unilaterally- and bilaterally-cauterized mice (n = 300 CTB$^+$ neurons, n = 2 sections per mice, n≥ 3 mice per stage and condition). Data shows mean ± SEM (error bars). p-value CTB$^+$ over DAPI/condition ≤0.0001 (Two-Way ANOVA: (h, i). F CTB$^+$ over DAPI/condition L2/3 (2, 28)= 48.20; F CTB$^+$ over DAPI/condition L4 F (2, 28)= 12.93; i, F CTB+ over DAPI/condition L2/3 (2, 27)= 17.91; F CTB+ over DAPI/condition L4 (2, 27) = 6.782). p-value vs WT *≤0.05, *** ≤0.001 (Post-hoc with Tukey test). Scale bars represent 500 µm (c, d) and 200 µm (e-g). Source data are provided as a Source Data file.

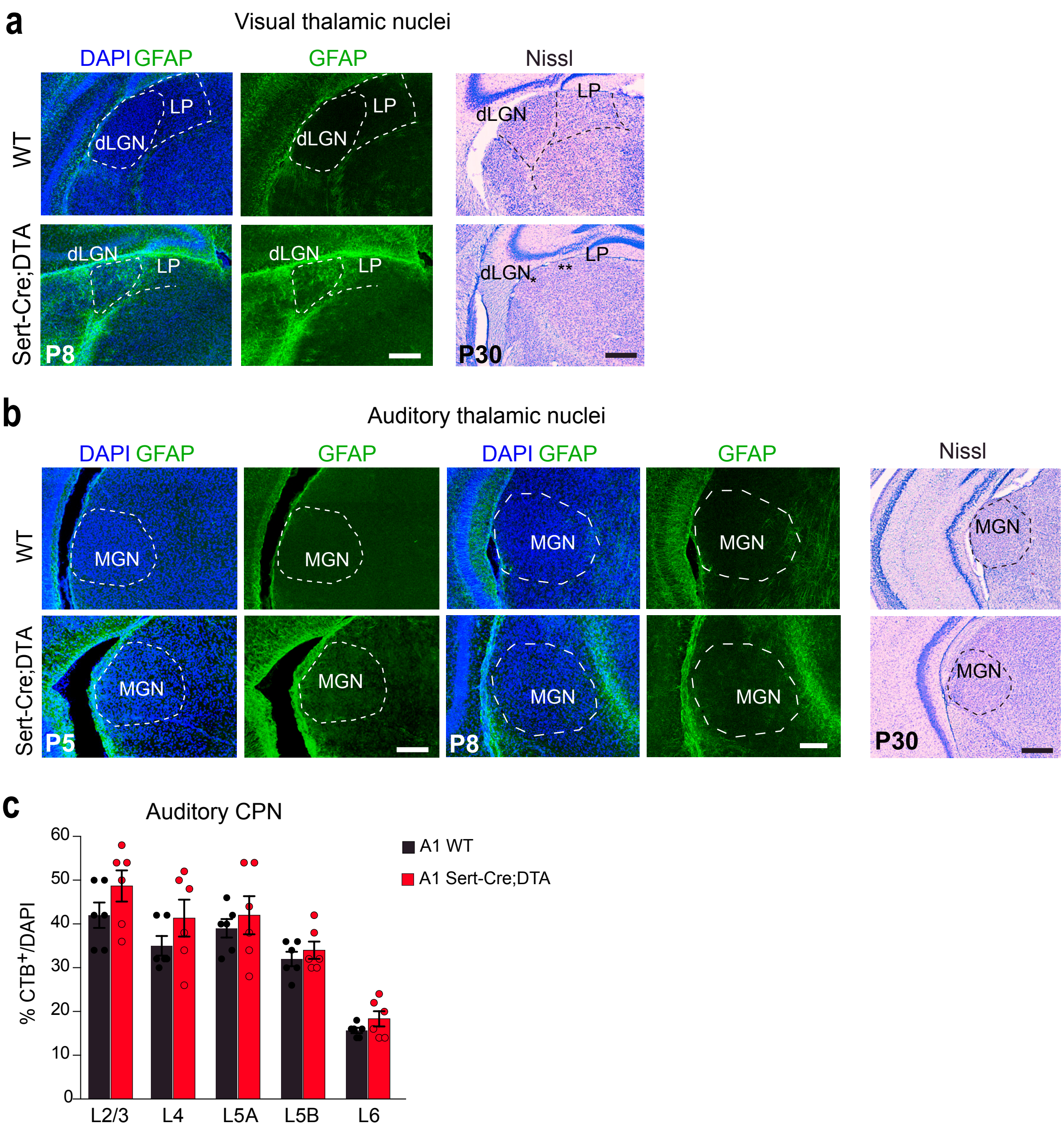

Supplementary Figure 8: Histology of Sert-Cre;DTA thalamic nuclei. a Left and center panels, GFAP staining in the dLGN and LP visual thalamic nuclei in WT and Ser-Cre;DTA at P8. Activated astrocytes are clearly visible in both dLGN and LP of Sert-Cre;DTA mice. Right panels show Nissl staining of P30 visual thalamic nuclei in WT and Sert-Cre;DTA. dLGN and LP are significantly reduced and damaged in Sert-Cre;DTA mice compared to WT. **b** GFAP staining in the medial geniculate nucleus (MGN) of WT and Sert-Cre;DTA at P5 and P8. No activated astrocytes were detected in WT or Sert-Cre;DTA in this thalamic region. Nissl staining (right panels) of MGN in P30 WT and Sert-Cre;-DTA mice revealed no significant differences in size or structure of MGN. **c** Quantifications of auditory CTB^{+} CPN in WT or Sert-Cre;DTA mice revealed no differences in the number or distribution of CPN among layers (n=300 CTB^{+} neurons, n= 6 sections, n= 3 mice). Data shows mean ± SEM (error bar). p-values not significant (Two-Way Anova). Scale bars represent 100 μm. Source data are provided as a Source Data file.

De León-Reyes et al., Figure S8

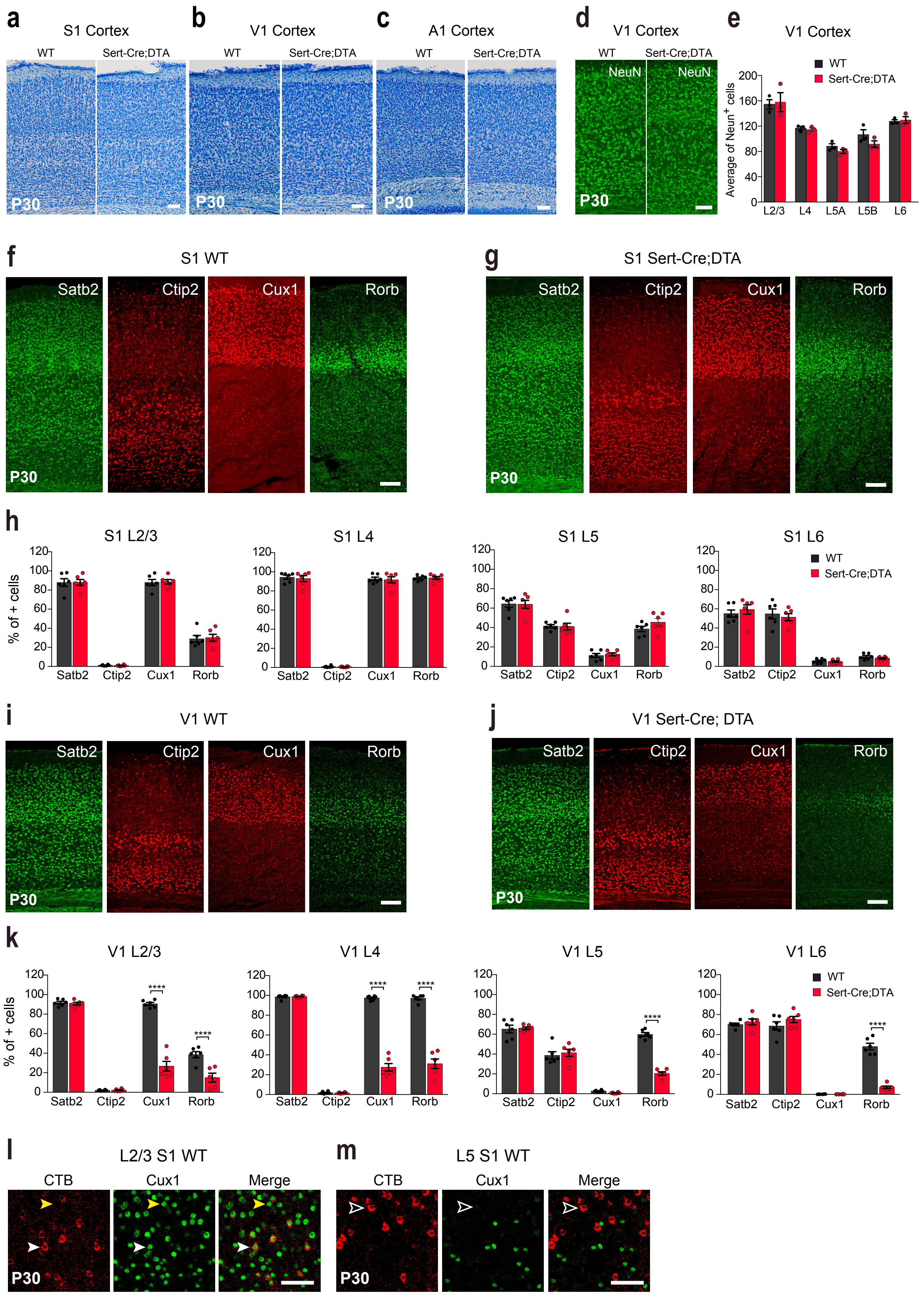

De León-Reyes et al., Figure S9

Supplementary Figure 9: Layer-specific transcription factors in WT and Sert-Cre;DTA.
a-c Nissl staining in P30 WT and Sert-Cre;DTA mice. No visible changes in cortical lamination and cortical numbers are detected between the two conditions. **d-e** Quantifications of NeuN+ cells in P30 V1 cortex revealed no significant differences in neuronal numbers between WT and Sert-Cre;DTA mice. (n=3500 neurons, n= 3 mice per condition).. p-values not significant (Student's t-test). **f-g** Laminar markers in S1 in P30 mice. **h** Quantification of cells stained with the indicated marker in the different layers as shown in (f, g). No significant differences (Two-Way ANOVA: F S1/S1-Sert-Cre; DTA L2/3 (1, 40) = 0.0438; F S1/S1Sert-Cre; DTA L4 (1, 40) = 0.183, F S1/S1Sert-Cre; DTA L5 (1, 40) = 0.73, F S1/S1Sert-Cre; DTA L6 (1, 40) = 0.0221). **i-j** Laminar markers in V1 cortex of P30 mice. **k** Quantification of cells stained with the indicated marker in the different layers as shown in (i, j). p-value laminar marker/Genotype ≤0.0001 (Two-Way ANOVA: F V1/ V1Sert-Cre; DTA L2/3 (1, 40) = 127.4, F V1/ V1Sert-Cre; DTA L4 (1, 40) = 450.4, F V1/ V1Sert-Cre; DTA L5 (1, 40) = 28.66, F V1/ V1Sert-Cre; DTA L6 (1, 40) = 20.57). p-value **** ≤0.001 (Post-hoc comparison with Sidak's test). **l** Co-labeling of Cux1 immunostaining and CTB+ in L2/3 neurons after P30 callosal injections shows that all CTB+ neurons in L2/3 are Cux1+ (white arrows) but not all Cux1+ neurons are CTB+ (yellow arrows). m In the same brain as (l), callosal neurons in L5 layer (CTB+) are not Cux1+ (outlined arrows). Data shows mean ± SEM (error bars). Scale bars represent 100 µm in all panels except 50 µm in (l, m). Source data are provided as a Source Data file.

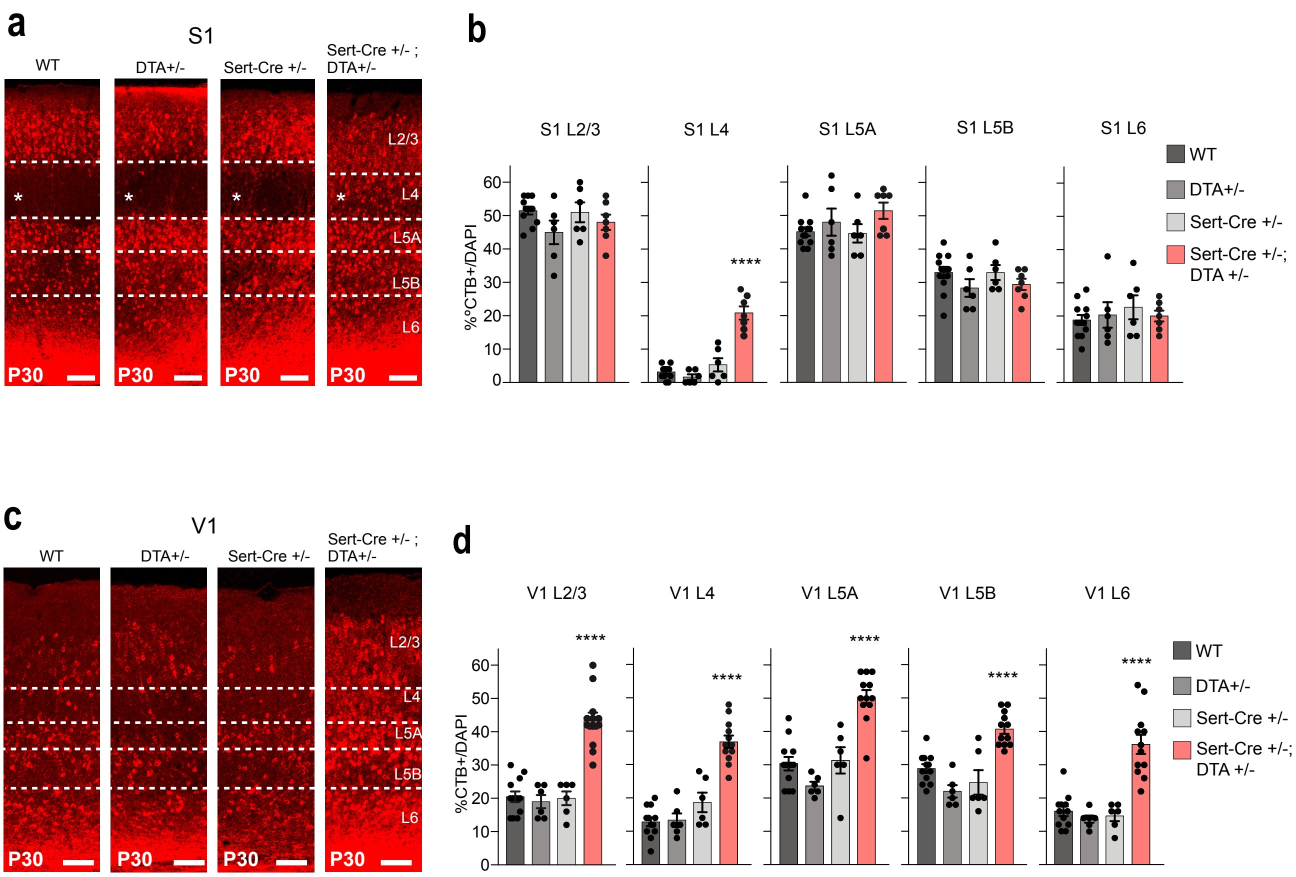

Supplementary Figure 10: CTB labeling in WT, control mice and DTA;Sert-Cre mutants. CTB labeling was analyzed in WT, control mice (single heterozygous, DTA$^{+/-}$ or Sert-Cre$^{+/-}$) and DTA;Sert-Cre mutants (double heterozygous DTA$^{+/-}$; Sert-Cre$^{+/-}$). **a** Coronal sections of brains injected in the CC at the somatosensory level at P30 with CTB-555. **b** Quantification of the proportion of CTB^{+} per DAPI^{+} nuclei in each layer and in each condition. Differences in CTB^{+} neurons are only detected in the Sert-Cre;DTA mice. (n = 300 cells; n = 2 sections per animal; n= 3 animals). ****p-value CTB L4/Genotype ≤0.0001 (One-Way ANOVA (comparing all genotypes): F CTB/Genotype L4 (3, 27 = 44.20. Non-significant for the rest of the layers, F CTB/Genotype L2/3 (3, 27) = 1.719; F CTB/Genotype L5A (3, 27) = 1.646; F CTB/Genotype L5B (3, 27) = 1.368; F CTB/Genotype L6 (3, 27) = 0.431. p-value CTB/Genotype non-significant (One-Way ANOVA (WT and control hets; n= 9 mice): F CTB/Genotype L2/3 (2, 21)= 2.344; F CTB/Genotype L4 (2, 21)= 2.38, F CTB/Genotype L5A (2, 21)= 0.465; F CTB/Genotype L5B (2, 21)= 1.403; F CTB/Genotype L6 (2, 21)= 0.551). **c** Coronal sections of brains injected in visual CC with CTB-555 at P30. **d** Quantification of the proportion of CTB^{+} per DAPI^{+} nuclei in each layer and in each condition. Differences in CTB^{+} neurons are only detected in the Sert-Cre;DTA mice. (n = 300 cells; n = 2 sections per animal; WT and mutants, n= 6 animals; DTA$^{+/-}$ and Sert-Cre$^{+/-}$, n= 3 animals each). ****p-value CTB/Genotype ≤0.0001 (One-Way ANOVA (n= 18 mice, all genotypes): F CTB/Genotype L2/3 (3, 32) = 34.39, F CTB/Genotype L4 (3, 32)= 42; F CTB/Genotype L5A (3, 32)= 25. 36; F CTB/Genotype L5B (3, 32)= 20. 24; F CTB/Genotype L6 (3, 32)= 25.86). p-value CTB/Genotype non-significant (One-Way ANOVA (n= 12 mice, WT and control hets.): F CTB/Genotype L2/3 (2, 21)= 0.162; F CTB/Genotype L4 (2, 21)= 2.52; F CTB/Genotype L5A (2, 21) =2.251; F CTB/Genotype L5B (2, 21) =2.921; F CTB/Genotype L6 (2, 21) =0.619). Scale bars represent 100 µm in a and b. Data shows mean ± SEM (error bars). Source data are provided as a Source Data file.

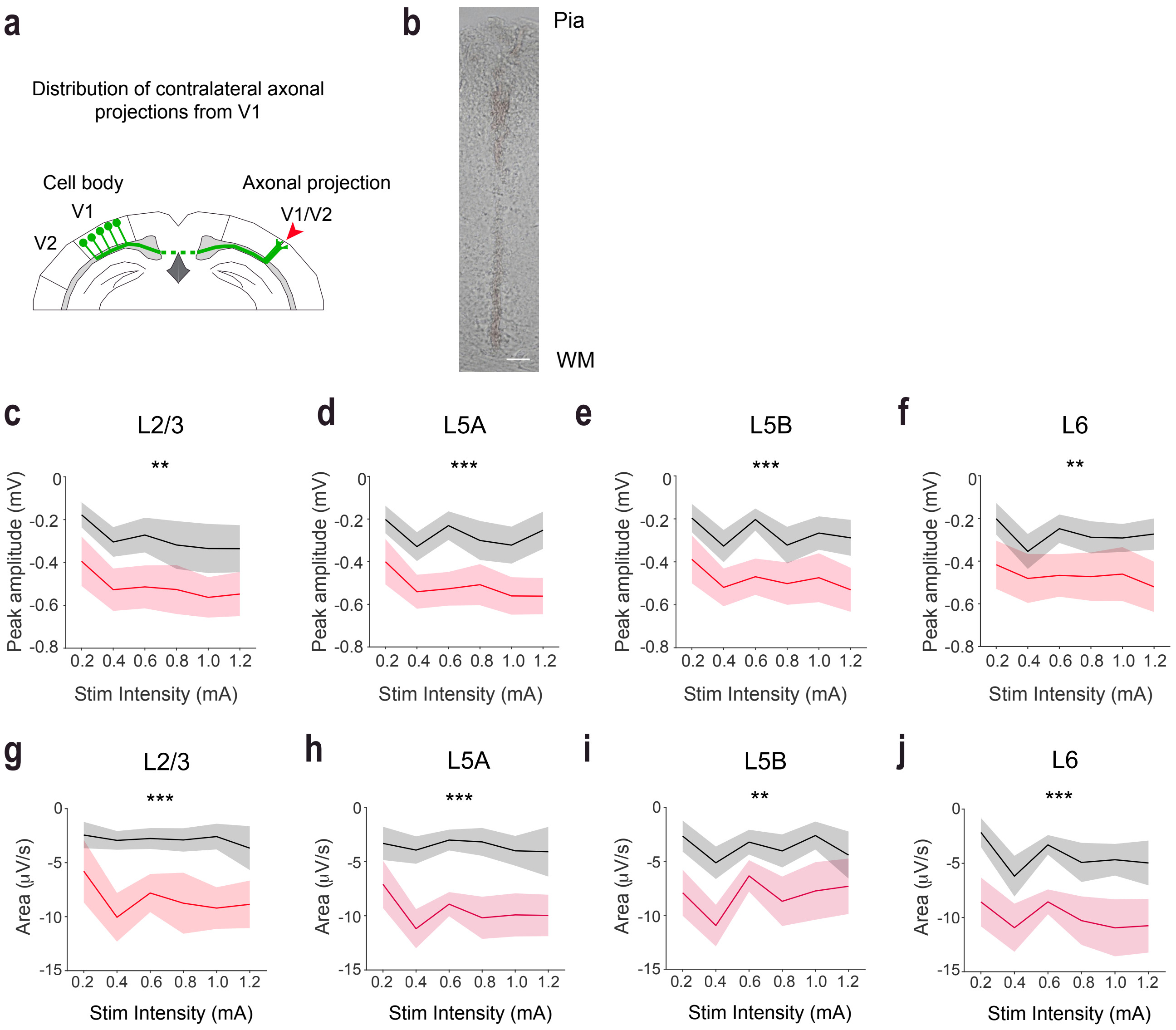

Supplementary Figure 11: Interhemispheric evoked activity in WT and Sert-Cre;DTA. a Schematic representation of interhemispheric connectivity in visual cortex. Recordings were performed at V1/V2, the known preferential location of V1 callosal projections. b Image of the linear probe location in V1/V2 cortical region after histological analysis of a mouse brain used for in vivo recordings confirming the proper cortical depths of recordings. **c-f** Peak amplitude for evoked LFP recordings in WT (black) and Sert-Cre;DTA (red) mice. (n = 9 replicates per mouse, n= 4 mice for WT and n= 3 mice for Sert-Cre;DTA). Data shows mean ± SEM (shaded). p-value Amplitude/Genotype ** ≤0.01, *** ≤0.001 (Three-Way ANOVA, F Amplitude/Genotype L2/3 (1, 124) =3.03, F Amplitude/Genotype L5A (1, 124) =7.58, F Amplitude/Genotype L5B (1, 124) =4.92, F Amplitude/Genotype L6 (1, 124) =3.06); **g-j**, Mean values of area for evoked LFP recordings at different layers in WT (black) and Sert-Cre;DTA (red). Note that Sert-Cre;DTA mice showed an increase in the area of evoked-LFP for all layers, which indicates a boosted interhemispheric excitability. (n = 9 replicates per mouse, n= 4 mice for WT and n= 3 mice for Sert-Cre;DTA). Data shows mean ± SEM (shaded). p-value Area/Genotype ** ≤0.01, *** ≤0.001 (Three-Way ANOVA: F Area/Genotype L2/3 (1, 124) =9.12, F Area/Genotype L5A (1, 124) =14.97, F Area/Genotype L5B (1, 124) =4.45, F Area/Genotype L6 (1, 124) =8.84. Scale bar represents 50 µm (b). Source data are provided as a Source Data file.

Supplementary Discussion

The analysis of the expression of layer-specific transcription factors (TF) in Sert-Cre;DTA and WT animals does not identify any transcription factor signature that defines CPN connectivity (Supplementary Fig. 9). We observed that the presence of callosal and non-callosal neurons in each layer of WT or Sert-Cre;DTA mutants does not correlate with the expression or absence of Satb2, Ctip2, Rorb, or Cux1. For example, in WT animals we identified positive and negative Cux1 expression in the CPN of S1 (Supplementary Fig. 9l-m). We found that callosal neurons of L5 WT animals do not express Cux1 (Supplementary Fig. 9m), while expression of Cux1 is required for callosal innervation of L2/3 neurons in WT animals [1]. Further, loss of Cux1 expression in V1L2/3 and V1L4 of Sert-Cre;DTA animals (Supplementary Fig. 9k) correlates with an increase in the number of CPN (Fig. 7e-l). Thus, these findings suggest that the expression of layer-specific TF can lead to either callosal or non-callosal fates depending on the circuit. This is in agreement with the idea that the postnatal determination of interhemispheric projections depends on multiple overlapping genetic networks. Our data helps to understand the origin of the heterogeneity of CPN populations and it is in clear agreement with studies in mice and monkeys that suggest there are multiple levels of complexity in the molecular identity of callosal neurons [2, 3, 4].

Supplementary Methods

<u>Determination of total CTB volumes of injection for different developmental stages:</u> The selected volumes were the minimum volumes required to maximize labeling of the area of interest and to fill both the dorsal and ventral areas of the CC track. Increasing these volumes of CTB did not increase the number of CTB labeled neurons in each area. The axons that cross through the CC are highly organized and fasciculate along the CC according to their cortical location. CTB volumes that do not completely span the CC or are injected only at the dorsal or ventral CC lead to preferential labeling of neurons that are positioned more medially or more laterally, respectively, in the cortex. Thus, the volumes we selected fulfilled the following criteria upon subsequent histological analysis. i) When observing the site of injection, CTB signal spread throughout the entire dorso-ventral width of the CC yet was restricted to only the CC and was not found in the cortical plate, subcortical territories or adjacent tracks such as the cingulum bundle. Supplementary figure 1 shows brains perfused

within two-hours post-injection to illustrate this restricted spreading. These images of P7 and P30 brains also show that the different volumes used for different developmental stages exhibit equivalent degrees of spreading. ii) For the rostro-caudal axis, we selected the minimum volume of injection that completely covered the CC path corresponding to the functional area of interest and that also labeled neurons in these areas in saturating numbers. For example, in adult mice, injecting 575 nl of CTB at Bregma -1.4 mm labeled somatosensory areas; after sectioning the brain coronally, we observed CTB spreading in sections spanning 1250 μm, which included the somatosensory barrels. In P7 mice, a volume of 230 nl injected at Bregma -1.1 mm was sufficient to cover 950 μm along the antero-posterior axis and to efficiently label equivalent somatosensory areas.

Anatomical references and coordinates to define the locations of functional areas for quantification: For adult coordinates, we used the atlas of Paxinos [5]. To set the coordinates to target the same areas in the developing brain we used the Developing Brain from Allen (http://atlas.brain-map.org/). For the somatosensory cortex of brains older than P21 we analyzed somatosensory areas located between Bregma -0.22 mm and -1.94 mm. S1 and S2 were identified based on the presence of the barrel structures in S1 labeled by VGLUT2 staining and/or by the coordinates along the medio-lateral (ML) axis: S1 from +2.5 mm to +3.8 mm, and S2 from +3.8 mm to +4.25 mm. Visual areas were quantified in regions from Bregma -2.46 mm to -3.64 mm along the antero-posterior (AP) axis: V1 from +1.3 mm to +3 mm, and V2 from +3 mm to +3.8 mm along the ML axis. For the auditory cortex in brains of animals older than P21 we analyzed areas from Bregma -2.18 mm to -3.08 mm along the AP axis and from +3.75 mm to +4.75 mm along the ML axis. Due to the horizontal orientation of the auditory field in the neocortex we added an additional reference to delineate A1 from A2 by measuring the dorso-ventral (DV) axis: A1 from +1.75 mm to +2.5 mm, and for A2 from +2.5 mm to +3.25 mm. To set the corresponding equivalent areas in the developing brain we used the Developing Brain from Allen (http://atlas.brain-map.org/).

Specificity and saturation of CTB injections: Compared to injections of CTB in the cortical plate, injections in the CC increase the efficiency of CPN labeling by ensuring contact with most commissural axons, including those with few or no branches in the cortical plate, and also label bundles of axons that innervate multiple cortical areas. Several controls and results supported the specificity and efficacy of injections in the CC. First, in CTB injections in the CC, symmetric labeling is detected in both hemispheres and no significant difference

was observed in the distributions of CTB^+ cells between hemispheres. CTB labeling is restricted to the cortex (Supplementary Fig. 1b) and contrary to CTB cortical plate injections (Supplementary Fig. 1j, white arrow), no labeling was detected in thalamic nuclei. This demonstrates that injections are restricted to inter-hemispheric axonal tracts located in the midline and cortical labeling was primarily restricted to callosal projections. Importantly, the numbers of callosal neurons in the different layers that we obtain when we inject CTB at P30 and later, are consistent with those obtained previously by traditional injections in the cortical plate, as well as with functional data obtained from electrophysiology [6, 7, 8, 9], indicating that we are not over- or underestimating the number of callosal projections with our CTB injections.

Second, we observe selective and specific distributions of callosal neurons in the different areas in the same animal at the same stage, and this was independent of the number of intervening days between the injection and the analysis (Supplementary Fig. 2a, b). This independence from the period of exposure to CTB was demonstrated by the following experiments: when CTB injections were performed at P3 and analyzed at P10 (seven days later) very few L2/3 cells were labeled (Supplementary Fig. 1c). However, when injections were performed at P5 and analyzed five days later (also at P10), most L2/3 were CTB^+ (Supplementary Fig. 2c and quantifications in Fig. 1 and 2). This is in agreement with the lack of callosal axons in L2/3 neurons at P3. At this stage, L2/3 neurons have just ended migration and initiated axonal extension. Similarly, injections at P1 and observed one month later at P32, revealed CTB labeling only in deeper layers (Supplementary Fig. 2d). These results do not support non-specific CTB labeling due to longer periods of CTB exposure or significant amounts of transneuronal labeling. Double injections at P5 and P7 (Supplementary Fig. 2c), P1 and P30 (Supplementary Fig. 2d), and P5 and P30 (Fig. 2e) using two fluorophores showed that all neurons labeled by the second CTB injections were positive for the first CTB injections. This demonstrates saturating conditions and high efficiency of CTB labeling. In contrast, significant proportions of neurons were labeled only by the first injections but not by the second injections, indicating that these fractions of neurons had eliminated their axons from the midline CC and thus demonstrating selectivity (Supplementary Fig. 2).

Finally, the visualization of the development of axonal projections using the Rorb-Cre line led to the same conclusions as those we drew from the CTB injections, and the refinement of GFP-labeled Rorb L4 axons mimicked the developmental dynamics observed with CTB injections (Figure 3). On the other hand, our control injections in the cortical plate in developing postnatal animals reproduced previously reported results, including a near-

complete absence of callosal L4 neurons (Supplementary Fig. 1i-l) [7]. We interpret that previous studies failed to detect all transient exuberant axons because projections with rapid dynamics in the cortical plate are inefficiently labeled using traditional injections in the cortical plate.

References

1. Rodriguez-Tornos FM*, et al.* Cux1 Enables Interhemispheric Connections of Layer II/III Neurons by Regulating Kv1-Dependent Firing. *Neuron* **89**, 494-506 (2016).

2. Molyneaux BJ, Arlotta P, Fame RM, MacDonald JL, MacQuarrie KL, Macklis JD. Novel subtype-specific genes identify distinct subpopulations of callosal projection neurons. *J Neurosci* **29**, 12343-12354 (2009).

3. Fame RM, Dehay C, Kennedy H, Macklis JD. Subtype-Specific Genes that Characterize Subpopulations of Callosal Projection Neurons in Mouse Identify Molecularly Homologous Populations in Macaque Cortex. *Cereb Cortex* **27**, 1817-1830 (2017).

4. Srinivasan K*, et al.* A network of genetic repression and derepression specifies projection fates in the developing neocortex. *Proc Natl Acad Sci U S A* **109**, 19071-19078 (2012).

5. Paxinos GaF, K.B.J. *The Mouse Brain in Stereotaxic Coordinates: Compact Second Edition.* . Elsevier Academic Press (2004).

6. Dehay C, Kennedy H, Bullier J. Callosal connectivity of areas V1 and V2 in the newborn monkey. *J Comp Neurol* **254**, 20-33 (1986).

7. Fame RM, MacDonald JL, Macklis JD. Development, specification, and diversity of callosal projection neurons. *Trends Neurosci* **34**, 41-50 (2011).

8. Meissirel C, Dehay C, Berland M, Kennedy H. Segregation of callosal and association pathways during development in the visual cortex of the primate. *J Neurosci* **11**, 3297-3316 (1991).

9. Schubert D, Kotter R, Staiger JF. Mapping functional connectivity in barrel-related columns reveals layer- and cell type-specific microcircuits. *Brain Struct Funct* **212**, 107-119 (2007).
