# Supplementary material for: Transient callosal projections of L4 neurons are eliminated for the acquisition of local connectivity
Source: Nat Commun. 2019 Oct 7;10:4549. doi: 10.1038/s41467-019-12495-w (PMC6779895; doi:10.1038/s41467-019-12495-w)
Supplement: Supplementary file 3 — Reporting Summary [file 41467_2019_12495_MOESM3_ESM.pdf]

nature research

Corresponding author(s): Marta Nieto

Last updated by author(s): Sep 5, 2019

# Reporting Summary

Nature Research wishes to improve the reproducibility of the work that we publish. This form provides structure for consistency and transparency in reporting. For further information on Nature Research policies, see Authors & Referees and the Editorial Policy Checklist.

## Statistics

For all statistical analyses, confirm that the following items are present in the figure legend, table legend, main text, or Methods section.

| n/a | Confirmed | |
|---|---|---|
| ☐ | ☒ | The exact sample size (*n*) for each experimental group/condition, given as a discrete number and unit of measurement |
| ☐ | ☒ | A statement on whether measurements were taken from distinct samples or whether the same sample was measured repeatedly |
| ☐ | ☒ | The statistical test(s) used AND whether they are one- or two-sided<br>*Only common tests should be described solely by name; describe more complex techniques in the Methods section.* |
| ☒ | ☐ | A description of all covariates tested |
| ☐ | ☒ | A description of any assumptions or corrections, such as tests of normality and adjustment for multiple comparisons |
| ☐ | ☒ | A full description of the statistical parameters including central tendency (e.g. means) or other basic estimates (e.g. regression coefficient) AND variation (e.g. standard deviation) or associated estimates of uncertainty (e.g. confidence intervals) |
| ☐ | ☒ | For null hypothesis testing, the test statistic (e.g. *F*, *t*, *r*) with confidence intervals, effect sizes, degrees of freedom and *P* value noted<br>*Give P values as exact values whenever suitable.* |
| ☒ | ☐ | For Bayesian analysis, information on the choice of priors and Markov chain Monte Carlo settings |
| ☒ | ☐ | For hierarchical and complex designs, identification of the appropriate level for tests and full reporting of outcomes |
| ☒ | ☐ | Estimates of effect sizes (e.g. Cohen's *d*, Pearson's *r*), indicating how they were calculated |

*Our web collection on statistics for biologists contains articles on many of the points above.*

## Software and code

Policy information about availability of computer code

| | |
|---|---|
| Data collection | *Provide a description of all commercial, open source and custom code used to collect the data in this study, specifying the version used OR state that no software was used.* |
| Data analysis | Fiji was used for quantifications on images (Nature methods 9(7): 676-682, PMID 22743772, doi:10.1038/nmeth.2019) |

For manuscripts utilizing custom algorithms or software that are central to the research but not yet described in published literature, software must be made available to editors/reviewers. We strongly encourage code deposition in a community repository (e.g. GitHub). See the Nature Research guidelines for submitting code & software for further information.

## Data

Policy information about availability of data

All manuscripts must include a data availability statement. This statement should provide the following information, where applicable:

- Accession codes, unique identifiers, or web links for publicly available datasets
- A list of figures that have associated raw data
- A description of any restrictions on data availability

The authors declare that the data supporting the findings of this study are available within the paper and its supplementary information files. The source data underlying main figures 1-7, and supplementary figures 1-11, are provided as a Source Data file available at: https://osf.io/m5sby/.

# Field-specific reporting

Please select the one below that is the best fit for your research. If you are not sure, read the appropriate sections before making your selection.

☒ Life sciences ☐ Behavioural & social sciences ☐ Ecological, evolutionary & environmental sciences

For a reference copy of the document with all sections, see nature.com/documents/nr-reporting-summary-flat.pdf

# Life sciences study design

All studies must disclose on these points even when the disclosure is negative.

| | |
|---|---|
| Sample size | There was no specific statistical method to determine sample size. Sample size was determined to be adequate based on the magnitude and consistency of measurable differences between groups. |
| Data exclusions | On principle, data were only excluded for failed injections. |
| Replication | Experiments were reliably reproduced. Each experimental condition was carried out with a minimum of three biological replicates. |
| Randomization | Not relevant to this study. No randomization of mice. Sample groups were assigned by genotype and treatment. |
| Blinding | Investigators were not blinded to mouse genotypes during experiments. |

# Reporting for specific materials, systems and methods

We require information from authors about some types of materials, experimental systems and methods used in many studies. Here, indicate whether each material, system or method listed is relevant to your study. If you are not sure if a list item applies to your research, read the appropriate section before selecting a response.

## Materials & experimental systems

| n/a | Involved in the study | |
|---|---|---|
| ☒ | ☐ | Antibodies |
| ☒ | ☐ | Eukaryotic cell lines |
| ☒ | ☐ | Palaeontology |
| ☐ | ☒ | Animals and other organisms |
| ☒ | ☐ | Human research participants |
| ☒ | ☐ | Clinical data |

## Methods

| n/a | Involved in the study | |
|---|---|---|
| ☒ | ☐ | ChIP-seq |
| ☒ | ☐ | Flow cytometry |
| ☒ | ☐ | MRI-based neuroimaging |

## Animals and other organisms

Policy information about studies involving animals; ARRIVE guidelines recommended for reporting animal research

| | |
|---|---|
| Laboratory animals | Slc6a4tm1(cre)Xz/J (Sert-Cre) mice (Jax stock #014554 and #009669), and R26:lacZbpA(flox)DTA (DTA) heterozygous mice 62 were maintained in heterozygosis in the C57BL/6JRccHsd background (Envigo laboratories, formerly Harlan). Sert-CRE;DTA mutants were obtained crossing heterozygous mice. WT animals were obtained from these colonies. Rorb-IRES2-Cre-D knockin (Rorb-Cre) mice were obtained from Jackson Mice repository (Jax stock #023526) and maintained in heterozygosis in the C57BL/6JRccHsd background. |
| Wild animals | *Provide details on animals observed in or captured in the field; report species, sex and age where possible. Describe how animals were caught and transported and what happened to captive animals after the study (if killed, explain why and describe method; if released, say where and when) OR state that the study did not involve wild animals.* |
| Field-collected samples | *For laboratory work with field-collected samples, describe all relevant parameters such as housing, maintenance, temperature, photoperiod and end-of-experiment protocol OR state that the study did not involve samples collected from the field.* |
| Ethics oversight | All animal procedures followed the European Commission guidelines (2010/63/EU) and were approved by the CSIC and the Community of Madrid Ethics Committees on Animal Experimentation in compliance with national and European legislation |

Note that full information on the approval of the study protocol must also be provided in the manuscript.
