# Supplementary material for: Transient callosal projections of L4 neurons are eliminated for the acquisition of local connectivity
Source: Nat Commun. 2019 Oct 7;10:4549. doi: 10.1038/s41467-019-12495-w (PMC6779895; doi:10.1038/s41467-019-12495-w)
Supplement: Supplementary file 2 — Peer Review File [file 41467_2019_12495_MOESM2_ESM.pdf]

Reviewers' comments:

Reviewer #1 (Remarks to the Author):

This is an important finding and the studies described in the paper explore both the phenomenon and its functional implications in admirable detail and thoroughness. Congratulations on a really excellent series of studies.

There are several issues which need to be addressed to make the claims and interpretations of the paper more robust and reliable.

The primary results of this paper depend on the uptake and retention of CTB from injections placed in the corpus callosum. Several considerations arise from the use of large quantities of CTB in the environment of developing neurons: for example, CTB has been shown to influence neurite outgrowth in culture by binding ganglioside GM1 and altering membrane properties (e.g. Rodriguez et al. J Neurosci. 2001;21:8387–95); although the techniques used to corroborate the tracing seem to validate this belief, it would be good to mention the possibility that the technique has influenced the outcome. Likewise, literature suggests that sprouting and injured axons are more likely to take up CTB, and that CTB may even spread transneuronally under conditions of injury (Lai et al., PLoS ONE 2015;10(12):e0144030). Placing pressure injections in a compact tissue such as white matter will injure axons, and so injection volume may be a determinant of the degree of uptake. How were the various injection volumes determined, and did the injection sites spread to an approximately equal extent of the cc at different ages?

Additionally, the span of P5 to P30 includes the most rapid phase of myelination in the mouse corpus callosum (Sturrock. Neuropathol Appl Neurobiol. 1980;6:415–20) – there is no discussion of this change or whether the drastically reduced free membrane area on myelinated axons would alter their uptake of CTB.

The use of t tests in a series of pairwise comparisons (i.e. Fig 1 k-o, Fig 6 h-l) is inappropriate for the conclusions drawn: the use of this many independent t-tests with no alpha correction risks type I errors, and each test’s significance refers only to the single timepoint at which the comparison is made. To examine the difference between S1 and V1 (Fig 1 k-o) or Sert-Cre;DTA vs WT conditions (Fig 6 h-l), across all timepoints, those factors need to be part of a model such as a 2-way ANOVA, to evaluate their overall effect.

Figures 4a and 5a are misleading: inputs to Po are represented as direct connections from the mystacial pad, and to VPM directly from whisker tips (the peripheral receptors and what drives them are also not represented in the diagram). Of course both of these nuclei are driven by many common afferents, rather than completely distinct sets as the diagram implies; the statement in the text that VPM is driven by tactile afferents and Po is driven by nociceptors is a questionable dichotomy and no supporting references are cited. These afferents of course terminate in the spinal trigeminal nucleus where second order neurons process them to activate the thalamic nuclei, which the diagrams should show rather than suggesting that the thalamus is driven by primary afferents. The major cortical drive to Po is worth adding since peripheral deafferentation will not reduce activity in Po to the same extent that it would in VPM, an important consideration when attributing axonal pruning guidance to thalamocortical activity.

It is also quite plausible that cautery of the mystacial pad may lead to chronic nociceptor activity or central sensitization – is there any evidence for or against this?

I found the argument generalising from layer 4 S1 targeted by Rorb-Cre electroporation, to the assertion that "all" pyramidal neurons make transient callosal connections difficult to accept; it should be made more explicit with justification. Even if so, in the discussion the claim that "all" neurons make

callosal connections should only refer to pyramidal neurons, of course.

Throughout the manuscript there are dozens of references to anatomical structures and stereotaxic coordinates, without any anatomy reference cited at any stage. This seems a major omission for a paper fundamentally grounded in anatomy.

Lastly, although it is appreciated that the finding of callosal exuberance followed by refinement is of great significance and illuminates a novel aspect of cortical development, the novelty of the idea of exuberance and refinement is considerably overstated throughout the paper. It has been known for nearly three decades that cortical neurons in rodents make exuberant and wildly diverse connections early in development before a process of refinement converges on their role in maturity – see for example Dennis O'Leary's papers on multi-targeted corticofugal axonal exuberance in early development, discussed in O'Leary & Koester, Neuron. 1993;10(6):991–1006. In this context, I was pleased but unsurprised to learn that a similar principle applies to transcallosal projection when I read this new set of studies. I think you have done a thorough and admirable job of describing and analyzing the phenomenon, and exploring its anatomical and functional consequences, but the claims of novelty need to be contextualized in the preceding literature, and the statement that this study "obligate[s] a paradigm shift" is excessive.

Reviewer #2 (Remarks to the Author):

During brain development, the phenomena of axonal exuberance followed by pruning is thought to play a role in establishing precise functional circuits. This paper presents further evidence for this very interesting and highly relevant phenomenon. Previous work by Innocenti and others have shown that an excess of cortical neurons send projections to the contralateral cortex through the corpus callosum, and that these exuberant axonal projections are then pruned (the majority retaining their ipsilateral projections) to form a final and stable pattern of contralateral innervation. The major addition of this study is the proposition that these exuberant projections come from a greater diversity of cortical layers than previously assumed, particularly layer 4, which, in the adult, predominantly contributes to short-range, local projection fates in the adult mouse.

This principal finding of callosal projections from layer 4 is the most novel part of the study and the intellectual contribution of the interpretations is important and interesting. There are a number of issues with the study however, and while many aspects of this manuscript are striking and novel to the field, the evidence for the claims made is unfortunately limited at present. An increased rigour with regards to the data and its analysis is needed and the conclusions should better reflect the results as presented, as there is currently a tendency for sweeping statements that are not borne out by the current data.

There are four major issues that impact interpretation of the data as presented. First, the n values on all experiments are too low for statistical comparisons which require the data to have a Gaussian distribution (such as a t-test). Only n=3 animals are quantified and only 2 sections per animal are counted in most experiments. In figure 3 (only) the authors switch to using Chi-squared analysis – but it's not clear why they switched to this non-parametric test even though n=3 animals is still used. It is also unclear why they use a Chi-squared test, which is appropriate for discrete/categorical data, on a seemingly continuous dataset. Second, as much of the data requires fluorescence levels, it is essential that no manipulation of the images occurred (such as increasing the brightness and contrast in some images in a dataset and not others, or in changing the imaging parameters on the microscope when collecting the images from different experimental groups that must be compared). Third, a significant issue needs to be addressed with regards to how the percentage of CTB+ neurons are determined. The authors have determined this by counting the number of DAPI-positive cells (as an indication of total cells) as the denominator and the number of CTB retrogradely labelled cells as the numerator at two different stages (P5 and P30). However, if the numerator remained the same, but the

denominator increased, then the % would appear to decrease, but this would not be due to pruning. As there is a significant increase in the number of cells in the cortex added between P5 and P30 (mostly glia- but nevertheless DAPI-positive) then the authors may be mis-interpreting their findings. Fourth, comparisons between groups are made which are not in line with the rationale and interpretation in the text. Why statistically compare S1 and V1 but not the proportion of cells labelled in each layer in one region, or the absolute number of cells labelled at each age?
Each of these points are addressed further below, with potential ways of addressing the issue provided.

To address point 1, further experiments are required on more animals to replicate the findings, provide a measure of the variance (at least n=6), and test for Gaussian distribution. Non-parametric tests could be used as an alternative but the minimal replication is still an issue.

To address point 2, the authors should provide assurance in the methods that the brightness and contrast has not been altered on any images in one group compared to another to enhance the result (see Fig. 2 compare DAPI and GFP in panels e and f compared g and h which are lighter overall). In Fig2 I – provide quantification of these images. It is not clear why quantification of k was performed in l as axons entering the white matter may not be callosal. Similarly, for the panels showing dynamics of contralateral innervation comparing L2/3 and L4 of S1 (Fig 3A-B) the exposure/brightness contrast is clearly different for these panels. E.g. there is some green fluorescence in S1 L4 P30 in the upper layers, but no axons in the white matter can be seen. It needs to be clarified whether and how these images were manipulated (same immuno conditions/microscopy exposure/post-capture manipulation with software etc?) and ideally the effect should be quantified (e.g. fluorescence intensity) with an appropriate normalisation (e.g. ipsilateral fluorescence intensity or background brain intensity, as has been done for other analysis within the manuscript).

To address point 3, the authors need to entertain the possibility that an alternative explanation for the main findings of axonal exuberance and pruning is that the number of neurons remain stable (or have altered dynamics to those shown) and that the composition of the surrounding cortical plate is changing, as is well known to occur throughout these periods. This problem arises because all of the quantifications are expressed as a percentage of DAPI positive nuclei, which does not reflect a stable number or population throughout development. It is therefore unsurprising that the percentage of CTB labelled neurons decreases, as the other later-born populations of the cortical plate (eg glia) increase in number. A complete switch to (or addition of) data presented as absolute cell numbers is critical to conclusively demonstrate the results reported, and without this, the manuscript's entire conclusions would need to be altered to reflect the uncertainty of this effect.

Additional specific comments below are predicated upon the assumption that a switch in all parts to a quantification of absolute cell number would reveal the same patterns as those reported by percentage:

- Interpretation: Throughout the introduction, results and discussion, there are repeated iterations of the statement (or variations thereof) that "all cortical neurons project callosally initially/ by default". The results of the paper themselves show that this is not true – e.g. L6 neurons of S1 never exhibit more than 60% of interhemispherically-projecting neurons out of all DAPI-positive nuclei. These conclusions should be modified to reflect the true nature of the findings, that select populations of all cortical layers (of unknown molecular identity) have the potential to project callosally initially.
- Many of the most striking and novel results presented by this paper are not the ones actually quantified and/or statistically analysed. For instance, nowhere is there a quantification of the absolute number of retrogradely labelled cells from the callosal tract in any cortical area throughout development, with a statistical comparison over time. The closest to this is Fig 1k-o, which compares areas (not ages), and which quantifies %CTB neurons. This quantification is not appropriate to show that there is an exuberance and refinement of callosally-projecting neurons, as it relies on a stability of cortical composition that simply isn't there. E.g., there is increased production of glia and

interneurons throughout development in the cortex that could mean a stable absolute number of callosally-projecting cells would appear to decrease as a percentage of total number, simply because of a progressive increase in other cell types. The qualitative images seem like this is not the case, but an appropriate quantification and statistical analysis comparing absolute number of contralaterally-projecting neurons throughout development is absolutely critical to conclusively demonstrate the results purported. This change to absolute number of cells would also increase the validity of subsequent analyses throughout the paper (e.g. sensory deprivation/thalamic manipulation etc), as they face the same problem of potentially manipulating cortical composition rather than refinement of callosal populations, as concluded by the authors. A figure/quantification showing the changing distribution of labelled cells as proportions per layer throughout development would also be welcome.

• Figure 2e-i shows qualitative descriptions of a striking decrease in contribution of L4 neurons to the callosal tract throughout development. It is unclear why the authors have not quantified this phenomena, instead quantifying (in a different set of ages) L4 neurons with axons entering the white matter (which could conceivably be projecting ipsilaterally; Fig 2 k,l). A rigorous quantification of this phenomena, showing supplementary insets of the electroporated cells at each age (such that cell body distribution can be assessed) and with appropriate normalisation, would greatly enhance the impact of this figure and the interpretation of the entire manuscript. Cell counts of the number of electroporated cells (absolute numbers, not percentages) would also help to interpret the issue of cell death raised on page 5 and go towards evidencing the statement that "These transient S1L4 callosal axons likely retract or disassemble, as we did not observe broken GFP axonal fragments in the CC or cortical plate, or neuronal death, during their period of refinement".

• Given that Fig. 4d-f aims to demonstrate that the refinement of contralateral projections from L4 S2 neurons is less than for L4 S1 neurons, it would be beneficial to quantify this effect and directly compare to L4 S1 quantifications throughout development (e.g. Fig 2e-i).

• In panels Fig1k-o, and again for Fig6h-l, the earliest age of injection is missing for one of the conditions. Upon cursory perusal of the figure, this may give an impression of differing dynamics between conditions that may not be the case. The reason for the absence of these datapoints should be clarified, and they should be incorporated if possible.

• On page 6, the text states that "The location of these contralateral columns is the same as SL2/3 callosal columns". It is unclear from this whether or not the authors have performed an electroporation in S1 or S2 (presumably at E15) to make this comparison. Further, the figures referred to in order to evidence this statement (Fig. 4h, i) do not show location at all in the context of medial-lateral position in the contralateral cortex, but rather demonstrate altered laminar distribution (again, unfortunately the proposed similarities/differences in location or laminar distribution are not demonstrated by quantification and statistical comparison).

• The representative images of CTB cells traced following bilateral cauterization (Fig. 5c and d) show a striking increase in labelled S1 L2/3 cells after bilateral cauterization at P30. However, this phenomena is not reflected in the quantifications, which reveal a decrease (Supplementary fig h). This is concerning because it is well known that the callosal tract is topographically arranged with respect to layers and mediolateral areas, such that a change in depth of injection could change the layer distribution and affect the results. These inconsistencies highlight the need for clear quantification of absolute numbers of retrogradely labelled cells, as well as sufficient animal numbers and appropriate statistical tests to be certain of the effects described.

• In Figure 5i, it would be of interest to statistically compare the dynamics of the Sert-Cre;DTA animals with the data collected from S2, as the authors are implying that this manipulation causes S1 to respecify to a more S2-like pattern of elimination. Also, are the absolute numbers of CTB positive neurons different between P5 and P30? – these should be compared within a group.

• For supplementary figure 8, cell counts and/or normalised fluorescent analysis of the transcription factors described is needed to recognise a significant change between conditions, as the effect appears to be more subtle than would be expected with a normal range of variability in staining among control animals.

• Text - Page 2: introduction "that facilitates the high functions of the human brain" Do the authors mean "that facilitates higher order functions of the human brain"? "capacity to emit interhemispheric projections" – wrong use of the term "emit"

• The English grammar needs to be checked throughout the manuscript.
• The title is not very informative and the abstract is quite confusing. It is recommended that the English grammar should be checked throughout.

Reviewer #1

This is an important finding and the studies described in the paper explore both the phenomenon and its functional implications in admirable detail and thoroughness. Congratulations on a really excellent series of studies.

There are several issues which need to be addressed to make the claims and interpretations of the paper more robust and reliable.

We sincerely thank the reviewer for his/her appreciation of our work, the highly constructive comments, and the detailed review of the manuscript. We found the comments useful and have incorporated responses that address them in the new version of our manuscript. We acknowledge that in answering his/her comments we have improved the description of the results, experimental methods and interpretations. A detailed point by point discussion of his/her comments is below.

The primary results of this paper depend on the uptake and retention of CTB from injections placed in the corpus callosum. Several considerations arise from the use of large quantities of CTB in the environment of developing neurons: for example, CTB has been shown to influence neurite outgrowth in culture by binding ganglioside GM1 and altering membrane properties (e.g. Rodriguez et al. J Neurosci. 2001;21:8387–95); although the techniques used to corroborate the tracing seem to validate this belief, it would be good to mention the possibility that the technique has influenced the outcome. Likewise, literature suggests that sprouting and injured axons are more likely to take up CTB, and that CTB may even spread transneuronally under conditions of injury (Lai et al., PLoS ONE 2015;10(12):e0144030).

In this revised version of the manuscript, we have made modifications clarifying these issues in the main text Methods section "CTB injections for retrograde labeling" (pg 13) and "Confocal imaging and Quantification" clarifying that quantifications were made in the contralateral hemisphere and describing rates of refinement in double injected animals (pg 15) and in Supplementary Text "CTB injections" discussing myelination.

We acknowledge the possibility that injury may facilitate CTB uptake or that CTB might alter membrane properties by decreasing neurite outgrowth as reported. However, it is important to emphasize that the quantifications of our injections are based on the number of CTB positive somas found in the hemisphere *contralateral* to the site of injection. This ensures that all neurons that are labeled with CTB possess at the moment of injection at least one inter-hemispheric projection in the contralateral territory, regardless of the growth or injury status of these projections. Thus, our conclusions should not be altered by this possibility because we are reporting solely the presence of axons in the contralateral hemisphere regardless of their stage, growth or number of branches.

One could argue that in our doubly-injected brains at different developmental time points (which in the early version referred to injections at P5-P7 and now include double injections at P1-P30 and P5-P30, new Supplementary Figure 2), the earlier injection could cause injury or alter axonal growth status and affect the results obtained from the latter injection time point. However, we have found this not to be the case. When we calculate the CTB distributions and refinement rates in singly injected (P5 or P30) and in doubly-injected (P5 and P30) brains, we find no differences in any cortical layer (new Supplementary Figure 2f), Results section pg 5, and Methods, pg 16. Thus, we believe that injury and/or altered growth status due to CTB injections has not affected our findings. Accordingly, we would like to point out that histology demonstrated very little brain damage after the small craniotomies and microinjections procedures (see Supplementary Figure 1a-h) and we have clarified this in the new Methods, pg 13.

Placing pressure injections in a compact tissue such as white matter will injure axons, and so injection volume may be a determinant of the degree of uptake. How were the various injection volumes determined, and did the injection sites spread to an approximately equal extent of the cc at different ages?

We realize this was not clearly explained in the experimental procedures. As explained above, we have now included a detailed description of the volumes of injection, the number of injection pulses, and the extent of CTB spreading in the CC in the main text Methods section "CTB injections for retrograde labeling" (pg 13-14), and of the experimental criteria to determine the volumes of injection in Supplementary section "CTB injections". In the new Supplementary Figure 1 we also show images illustrating the site of injection and the spreading.

As in most injection protocols, to minimize damage, injections were made progressively at 23nl per injection pulse with a maximum frequency of one pulse per second (hence 23nl/s) until the desired total volume was achieved. Although we cannot discard the possibility of injury, we never detected obvious signs of relevant damage in the cortical plate, in the CC or in callosal GFP axons after our

injections (see new Supplementary Figure 1). This is now mentioned in the main text, Methods section "CTB injections for retrograde labeling" (pg 13-14).

In regard to the determination of the various injection volumes of CTB for the different developmental stages, we experimentally determined that these volumes were the minimum volumes required to maximize labeling of the area of interest. Increasing these volumes of CTB did not increase the number of CTB labeled neurons in each particular area. These volumes were demonstrated to fill both the dorsal and ventral areas of the CC track, an important point because the axons that cross through the CC are topographically organized and fasciculate along the CC according to the cortical location of the efferent neurons. Accordingly, CTB that is injected only at the dorsal or ventral CC leads to a preferential labeling of neurons that are positioned more medially or more laterally, respectively, in the cortex. We made sure that the volumes we selected fulfilled the following criteria upon subsequent histological analysis. **i)** When observing the site of injection, CTB signal spread throughout the entire dorso-ventral width of the CC yet was restricted to only the CC and was not found in the cortical plate, subcortical territories or adjacent tracks such as the cingulum bundle. Supplementary Figure 1 now shows brains perfused within two hours post-injection to illustrate this restricted spreading. These images of P7 and P30 brains also show that the different volumes used for different developmental stages exhibit equivalent degrees of spreading. **ii)** For the rostro-caudal axis, we selected the minimum volume of injection that completely covered the CC path corresponding to the functional area of interest and that also labeled neurons in these areas in saturating numbers. For example, in adult mice, injecting 575 nl of CTB at Bregma -1.4 mm labeled somatosensory areas; after sectioning the brain coronally, we observed CTB spreading in sections spanning 1250 µm, which included the somasotosensory barrels. In P7 mice, a volume of 230 nl injected at Bregma -1.1 mm was sufficient to cover 950 µm along the antero-posterior axis and to efficiently label equivalent somatosensory areas. All these methodological specifications are now described in Methods of the main text and included in a new section titled **"**Determination of total CTB volumes of injection for different developmental stages" in the Supplementary Text (pg 2).

Additionally, the span of P5 to P30 includes the most rapid phase of myelination in the mouse corpus callosum (Sturrock. Neuropathol Appl Neurobiol. 1980;6:415–20) – there is no discussion of this change or whether the drastically reduced free membrane area on myelinated axons would alter their uptake of CTB.

One of the main novel findings of our work is the detection of transient axons early in development and the fact that these axons are detected in most of the neuronal populations that are not callosal in the adult. Importantly, this conclusion is not affected by the possibility that changes in myelination would diminish the efficiency of labeling because neurons that are identified as CTB positive in our experiments have ipso facto an axon in the CC of the contralateral hemisphere. Nevertheless, we had considered during our initial experiments a possible effect of myelination. This could change the efficiency of labeling and diminish the number of detected callosally projecting cells at a given stage resulting in underestimation of the

number of callosal neurons and overestimation of refinement. This is one of the main reasons why we decided to confirm the refinement rates we observed using an independent method, the tracing of genetically labeled Rorb axons. These experiments confirmed the results we observed with our CTB injections. Because of this, and because a number of other reasons detailed below, we concluded that artifacts due myelination are very unlikely. This is now described and discussed in the Supplementary Text section “CTB injections” (Supplementary Text, pg 1), and in the Discussion (pg 11).

The main reasons to discard any significant effects due to myelination were: First, the overall results indicate that our labeling efficiency is high (equal or close to saturating conditions) at all stages. The numbers of callosal neurons in the different layers that we obtain at mature stages are consistent with those obtained previously by traditional injections in the cortical plate, as well as with functional data obtained from electrophysiology (Dehay C, et al., J Comp Neurol. 1986 Dec 1;254(1):20-33; Meissirel et al., J. Neurosci. 1991 Nov;11(11):3297-316; LaMantia and Rakic J Comp Neurol. 1994 Feb 15;340(3):328-36; Schubert et al., Brain Struct Funct. 2007 Sep;212(2):107-19; Fame et al., Trends Neurosci. 2011 Jan;34(1):41-50). This indicates that we are not underestimating the number of callosal projections with our CTB injections. Second, myelination should equally affect all cortical areas, yet we see selective and specific distributions of callosal neurons in the different areas. Finally, as mentioned above, the visualization of the development of axonal projections using the Rorb-Cre line led to the same conclusions as those we drew from the CTB injections. These included the temporal timing of refinement of GFP-labeled Rorb L4 axons, which mimicked the developmental dynamics observed with CTB injections. Electroporated Rorb-Cre animals also showed that the absence of GFP axons in S1L4 neurons coincided with the absence of CTB labeling, while in S2 the presence of axonal connectivity correlated with CTB^+ cells in L4 (Fig. 4j). Results when electroporating auditory territories were also consistent with CTB results (Supplementary Figure 7f). We believe that this *in vivo* axonal tracing is the most reliable confirmation of both the specificity and the efficiency of our CTB experiments. We have briefly discussed these ideas in the revised manuscript in the Discussion section (pg 11).

The use of t tests in a series of pairwise comparisons (i.e. Fig 1 k-o, Fig 6 h-l) is inappropriate for the conclusions drawn: the use of this many independent t-tests with no alpha correction risks type I errors, and each test’s significance refers only to the single timepoint at which the comparison is made. To examine the difference between S1 and V1 (Fig 1 k-o) or Sert-Cre;DTA vs WT conditions (Fig 6 h-l), across all timepoints, those factors need to be part of a model such as a 2-way ANOVA, to evaluate their overall effect.

We sincerely thank the reviewer for this very constructive suggestion. We have now applied ANOVA tests to appropriately compare different conditions. These statistic tests are now described in the Methods section “Statistical analysis” (pg 17). They are also mentioned in all relevant Figure panels and Figure legends, where relevant p values are indicated.

Figures 4a and 5a are misleading: inputs to Po are represented as direct connections from the mystacial pad, and to VPM directly from whisker tips (the peripheral receptors and what drives

them are also not represented in the diagram). Of course both of these nuclei are driven by many common afferents, rather than completely distinct sets as the diagram implies; the statement in the text that VPM is driven by tactile afferents and Po is driven by nociceptors is a questionable dichotomy and no supporting references are cited. These afferents of course terminate in the spinal trigeminal nucleus where second order neurons process them to activate the thalamic nuclei, which the diagrams should show rather than suggesting that the thalamus is driven by primary afferents. The major cortical drive to Po is worth adding since peripheral deafferentation will not reduce activity in Po to the same extent that it would in VPM, an important consideration when attributing axonal pruning guidance to thalamocortical activity.

We have modified the diagram and the text. In the original version, our intention was to simplify the diagram and to convey that S1L4 and S2L4 neurons integrate circuits with distinct functional responses (Bosman et al., Front Integr Neurosci. 2011. Oct 4;5:53. Frangeul et al., Nature. 2016 Oct 6;538(7623):96-98; Goldin et al., Nat Commun. 2018 Oct 3;9(1):4053). However, the reviewer is absolutely correct and we apologize for causing confusion.

The new diagrams in Fig 4a and 5a and f, have been modified to reflect the Infraorbital nerve (ION) as the major input from peripheral receptors to the brainstem and to show that primary afferents relay their information in the appropriate brainstem regions rather than directly to the thalamus. We have also included the barrel field representation along the lemniscal pathway (Brainstem, Thalamus and Neocortex) in Fig 4a. Also, we illustrate the S1L5 cortical input to the Po. We fully agree that these details can be important for the correct interpretation of the data. Accordingly, we have modified the text to remove the description of axonal inputs as an absolute segregation and now clarify the complexity of the afferent inputs to thalamic nuclei. This is included in the text in Result sections (pg 7):

Sensory information from the facial whiskers and the whisker pad are integrated into the SS cortex by the lemniscal and paralemniscal pathways. While S1L4 is the main target of the lemniscal pathway vía ventral posteromedial nucleus (VPM) thalamocortical axons, S2L4 is mainly innervated by the medial posterior nucleus (Po). The VPM primarily conveys input from the principal trigeminal nuclei (PrV; lemniscal pathway) and the Po conveys input from the spinal trigeminal nuclei (SpVi; paralemniscal pathway) (Fig. 4a). Even though these two trigemino-thalamo-cortical pathways share many common afferents, S1L4 and S2L4 neurons are differentially activated by tactile and nociceptive stimuli. While S1L4 neurons respond to tactile stimuli *in vivo* and are not activated by nociceptive input from the whisker pad, S2L4 neurons respond to both nociceptive and tactile stimuli (Bosman et al., Front Integr Neurosci. 2011. Oct 4;5:53. Frangeul et al., Nature. 2016 Oct 6;538(7623):96-98; Goldin et al., Nat Commun. 2018 Oct 3;9(1):4053).

It is also quite plausible that cautery of the mystacial pad may lead to chronic nociceptor activity or central sensitization – is there any evidence for or against this?

We have not found any reported evidence discarding or supporting this form of plasticity.

I found the argument generalising from layer 4 S1 targeted by Rorb-Cre electroporation, to the assertion that "all" pyramidal neurons make transient callosal connections difficult to accept; it should be made more explicit with justification. Even if so, in the discussion the claim that "all" neurons make callosal connections should only refer to pyramidal neurons, of course.

We have made modifications in the text to make sure that our interpretations are clearly explained and to avoid overgeneralizations. We intended to convey that our alternative approach using Rorb-Cre animals to visualize developmental callosal axons fully validated our CTB experiments. These two approaches demonstrated similar dynamics of refinement, particularly in S1L4 neurons, which are known to project only locally in the adult. This validation of our CTB experiments helps demonstrate that most if not all, pyramidal neurons project callosally initially. Nevertheless, we can only agree with the reviewer that a more neutral presentation of the results benefits the manuscript and we have changed the text accordingly:

Abstract: Before: “Using a novel axonal-retrotracing strategy we instead demonstrate that virtually all cortical pyramidal neurons project calosally initially.” Now: “Using a novel axonal-retrotracing strategy we instead demonstrate that most cortical pyramidal neurons project calosally initially.”

Main text. Result section (pg 3), before: “demonstrating the bonafide early callosal fate of all cortical neurons.” Now: “demonstrating the bona fide early callosal fate of these cortical neurons.”

Main Text: Result section (pg 5), before: “Altogether, these results indicate that virtually all cortical pyramidal neurons extend transient exuberant interhemispheric axons by default.” Now: “Altogether, these results indicate that the vast majority of cortical pyramidal neurons extend transient exuberant interhemispheric axons by default.”

Main Text: Results section (pg 9), before: “The plasticity we observed in S1L4 neurons suggested that there exists the potential for terminal callosal connectivity in all cortical neurons” is changed to “The plasticity we observed in S1L4 neurons suggested that there exists the potential for terminal callosal connectivity in most cortical neurons”.

Discussion (pg 11): “The main finding of our work is the demonstration that all cortical neurons display transient callosal axons that are subsequently eliminated” now is “The main finding of our work is the demonstration that most cortical neurons display transient callosal axons that are subsequently eliminated.”

Throughout the manuscript there are dozens of references to anatomical structures and stereotaxic coordinates, without any anatomy reference cited at any stage. This seems a major omission for a paper fundamentally grounded in anatomy.

This is now included in the main text (Methods, “Confocal Imaging and Quantifications” pg 15-16 and Supplementary Text, “Anatomical references and coordinates” (Supplementary Text, pg 2,3). Again, we thank the reviewer for this constructive comment. For adult coordinates we used the atlas of Paxinos (Paxinos, G. and Franklin, K.B.J. (2004) The Mouse Brain in Stereotaxic

Coordinates: Compact Second Edition. Elsevier Academic Press, San Diego, CA.). To set the coordinates to target the same areas in the developing brain we used the Developing Brain from Allen (http://atlas.brain-map.org/). As mentioned above, we have also included a more detailed explanation of coordinates to define the location of functional areas in the adult in Supplementary Text (pg 2,3).

Lastly, although it is appreciated that the finding of callosal exuberance followed by refinement is of great significance and illuminates a novel aspect of cortical development, the novelty of the idea of exuberance and refinement is considerably overstated throughout the paper. It has been known for nearly three decades that cortical neurons in rodents make exuberant and wildly diverse connections early in development before a process of refinement converges on their role in maturity – see for example Dennis O'Leary's papers on multi-targeted corticofugal axonal exuberance in early development, discussed in O'Leary & Koester, Neuron. 1993;10(6):991–1006. In this context, I was pleased but unsurprised to learn that a similar principle applies to transcallosal projection when I read this new set of studies. I think you have done a thorough and admirable job of describing and analyzing the phenomenon, and exploring its anatomical and functional consequences, but the claims of novelty need to be contextualized in the preceding literature, and the statement that this study "obligate[s] a paradigm shift" is excessive.

One of the main novel conclusions from our data is that those areas and layers that were thought to never project callosally in fact do extend developmental axons that are capable of functional connectivity. Our intention was to emphasize that there is potential interhemispheric connectivity in many more neurons than previously thought and that it might be interesting to revisit the concept of how early callosal fate is selected. Nevertheless, as above, we can only agree with the reviewer that it is best to moderate our statements and let the data speak for itself. In this new version we have modified this and other similar statements. We hope that we have found a better balance between precise descriptions and interpreting the impact of our findings and thank him/her for this comment.

Changes in the Main text, Discussion (pg 12): Before "Thus, the mechanisms described here obligate a paradigm shift and provide novel understanding of the complex connectivity of the brain". Now: "Thus, the mechanisms described here provide a novel understanding of how the complex connectivity of the brain is achieved and broaden our perspective of its potential for plasticity".

Reviewer #2 (Remarks to the Author):

During brain development, the phenomena of axonal exuberance followed by pruning is thought to play a role in establishing precise functional circuits. This paper presents further evidence for this very interesting and highly relevant phenomenon. Previous work by Innocenti and others have shown that an excess of cortical neurons send projections to the contralateral cortex through the corpus callosum, and that these exuberant axonal projections are then pruned (the

majority retaining their ipsilateral projections) to form a final and stable pattern of contralateral innervation. The major addition of this study is the proposition that these exuberant projections come from a greater diversity of cortical layers than previously assumed, particularly layer 4, which, in the adult, predominantly contributes to short-range, local projection fates in the adult mouse.

This principal finding of callosal projections from layer 4 is the most novel part of the study and the intellectual contribution of the interpretations is important and interesting. There are a number of issues with the study however, and while many aspects of this manuscript are striking and novel to the field, the evidence for the claims made is unfortunately limited at present. An increased rigour with regards to the data and its analysis is needed and the conclusions should better reflect the results as presented, as there is currently a tendency for sweeping statements that are not borne out by the current data.

We thank the reviewer for his/her positive comments regarding the significance of our data. We also thank the reviewer for the criticisms and detailed revision. We believe that by answering his/her comments we have improved the scientific rigor of our analyses and the manuscript overall.

There are four major issues that impact interpretation of the data as presented. First, the n values on all experiments are too low for statistical comparisons which require the data to have a Gaussian distribution (such as a t-test). Only n=3 animals are quantified and only 2 sections per animal are counted in most experiments. In figure 3 (only) the authors switch to using Chi-squared analysis – but it's not clear why they switched to this non-parametric test even though n=3 animals is still used. It is also unclear why they use a Chi-squared test, which is appropriate for discrete/categorical data, on a seemingly continuous dataset.

Second, as much of the data requires fluorescence levels, it is essential that no manipulation of the images occurred (such as increasing the brightness and contrast in some images in a dataset and not others, or in changing the imaging parameters on the microscope when collecting the images from different experimental groups that must be compared). Third, a significant issue needs to be addressed with regards to how the percentage of CTB+ neurons are determined. The authors have determined this by counting the number of DAPI-positive cells (as an indication of total cells) as the denominator and the number of CTB retrogradely labelled cells as the numerator at two different stages (P5 and P30). However, if the numerator remained the same, but the denominator increased, then the % would appear to decrease, but this would not be due to pruning. As there is a significant increase in the number of cells in the cortex added between P5 and P30 (mostly glia- but nevertheless DAPI-positive) then the authors may be mis-interpreting their findings. Fourth, comparisons between groups are made which are not in line with the rationale and interpretation in the text. Why statistically compare S1 and V1 but not the proportion of cells labelled in each layer in one region, or the absolute number of cells labelled at

each age?
Each of these points are addressed further below, with potential ways of addressing the issue provided.

To address point 1, further experiments are required on more animals to replicate the findings, provide a measure of the variance (at least n=6), and test for Gaussian distribution. Non-parametric tests could be used as an alternative but the minimal replication is still an issue.

We acknowledge that a different statistical analysis from the one we applied is more appropriate to analyze the significance of the refinement we observe during development and we thank the reviewer for pointing this out. Following consultation with an expert in statistics and in agreement with the recommendations of Reviewer #1, we have performed 3-, 2-, 1- way ANOVA analyses (after testing for normal distribution) to evaluate the overall refinement in each area as well as to compare between areas (S1 vs S2 or S1 vs V1, for example), or to compare between experimental conditions (sensory deprivation or SertCre:DTA mutants compared to controls). These analyses are now described in the Methods section (pg 17) and are included in all figure legends.

In reference to the reviewer's 4th point, our new ANOVA analyses demonstrate that, not only do all layers undergo significant refinement, but this refinement differs between areas (S1 vs V1, for example; see Figure 1k-o, or Supplementary Figures 6 and 7). As mentioned in the Results section (pg 5), these distinct refinement dynamics in distinct cortical areas led us to postulate a role for circuit activity (pg 8).

In regard to sample size, we would like to point out that our conclusions are obtained from a number of animals that is greater than 15 mice per condition for these types of comparisons. In the case of refinement in WT animals, when comparing refinement between areas, the sample size is n=24 and includes 6 developmental stages with a minimum of 3 mice per stage (n=3 mice for P3, P5, P7, P14, and P21, and now n=6 mice for P5 and P30). Our conclusions are based on the trend of the number of callosal projections per stage and per area within this sample size. In other words, our conclusions are based on our observations of the refinement curves, not comparisons between two specific stages or between two conditions at a single stage. We apologize as we had failed to apply the proper statistical analysis that takes into account the curve as a whole, perhaps causing the incorrect impression that our conclusions were based on comparisons of 3 mice per condition. The new ANOVA analysis corrects this flaw. It confirms that the number of mice we employed was sufficient to detect curves of refinement and to support our conclusions. We would also like to mention that, nevertheless, we decided to increase the number of mice for the two developmental stages that we have used as the initial and end points of refinement: P5 and P30. These two time points were used to compare refinement rates using different quantification methods (see below). In the revised version, we have performed the quantifications CTB^{+}/DAPI and other alternative quantifications that the reviewer suggested using a larger sample size (n=6). Comparisons between these two stages resulted in significant p

values regardless of the method of quantification (see below and new Supplementary Figures 2e, f and 3).

Hence, given the statistical significance of our data when the correct analysis was applied, we are sure that the reviewer will agree that arbitrarily increasing all data points to 6 animals per stage is not justified or necessary as it would be extremely time-consuming, would not advance the findings of the study, and would not comply with the ethical obligation to minimize animal use.

Again, we apologize for the confusion caused by not applying the correct statistical analysis in the first place. We sincerely thank the reviewer for contributing to this important point.

In regard to the chi-squared test, we have confirmed that for comparisons of populations in which axons are categorized as either positive or negative for synaptophysin, a Chi square analysis is appropriate.

To address point 2, the authors should provide assurance in the methods that the brightness and contrast has not been altered on any images in one group compared to another to enhance the result (see Fig. 2 compare DAPI and GFP in panels e and f compared g and h which are lighter overall). In Fig2 l – provide quantification of these images. It is not clear why quantification of k was performed in l as axons entering the white matter may not be callosal. Similarly, for the panels showing dynamics of contralateral innervation comparing L2/3 and L4 of S1 (Fig 3A-B) the exposure/brightness contrast is clearly different for these panels. E.g. there is some green fluorescence in S1 L4 P30 in the upper layers, but no axons in the white matter can be seen. It needs to be clarified whether and how these images were manipulated (same immuno conditions/microscopy exposure/post-capture manipulation with software etc?) and ideally the effect should be quantified (e.g. fluorescence intensity) with an appropriate normalisation (e.g. ipsilateral fluorescence intensity or background brain intensity, as has been done for other analysis within the manuscript).

In the methods section of this revised version we include a detailed description of how images were acquired and processed for unbiased quantifications (Methods, "Confocal Imaging and Quantification" pg 16). Images were not manipulated to alter the results. Many of the original tile scan images can be seen now in the corresponding supplemental materials and all values obtained from images can be observed in data source files. We have also quantified the requested images that were of concern to the reviewer. This added information is detailed below.

Acquisition and quantifications were performed as published (Rodríguez-Tornos et al., Neuron. 2016 Feb 3;89(3):494-506; Briz CG et al., J Vis Exp. 2017 Feb 15;(120)) following methodologies similarly used by others (Courchet et al., Cell. 2013 Jun 20;153(7):1510-25; Mita S, et al., Cereb Cortex. 2015 Nov;25(11):4111-25; Mizuno et al., Eur J Neurosci. 2010 Feb;31(3):410-24; and Plas et al., Vision Res. 2004 Dec;44(28):3357-64). The Methods section (pg 16) now states: Acquisition and quantifications of Fluorescence signal was performed as published (Rodríguez-Tornos et al., Neuron. 2016 Feb 3;89(3):494-506; Briz CG et al., J Vis Exp. 2017 Feb 15;(120). During acquisition, signal detectors are set to ensure linearity and equivalent conditions between

all samples by determining minimum and maximum threshold signals. The threshold for background noise was determined using regions outside of electroporated areas (Plas et al., Vision Res. 2004 Dec;44(28):3357-64). The maximum threshold signal was set by ensuring that no pixels were saturated. This avoids an artefactual increase in low signals in order to ensure linearity. Quantifications were performed by converting pixel intensity into binary values using Fiji. Contralateral fluorescence values are given relative to the values of fluorescence of the ipsilateral electroporated areas that were outlined manually, and normalized to control conditions.

Precisely because we did not modify fluorescence intensity and treated the images with extreme care to avoid biased manipulations, the reader can appreciate the details of the fluorescence signal in each panel. Indeed, in Fig 3B there is some green fluorescence of S1L4 axons in P30 in the contralateral side and only a few axons in the white matter that are even more difficult to see. These signals are real and we did not want to manipulate them artificially. They simply reflect the few axons that can be observed in P30 brains when we target S1 in our electroporations. The lack of clear GFP signal in the CC is also real and coherent with our data. Most likely the signal in the cortical plate corresponds to the branches of the few axons of the electroporated neurons that remain callosal after refinement (4%). Alternatively, it could reflect the few S2L4 neurons that are labeled in our electroporations as it is virtually impossible to control the area of electroporation to target S1 exclusively. Axons responsible for this contralateral signal cannot be detected in the WM either because there are only a few axons, each giving rise to more easily detectable branches in the cortical plate, or because the few that do cross do so in more anterior or posterior trajectories. The latter reasoning can be explained by the fact that CC trajectories are not parallel to the histological plane, but instead follow a butterfly shape.

In regard to the quantifications made of axons entering the white matter ("*It is not clear why quantification of k was performed in l as axons entering the white matter may not be callosal*") (Fig 2 panels now k-m). We agree that these may not be callosal but we would again like to clarify that our intention is precisely to call attention to the remodeling of these projections and to describe it in great detail. Adult mature S1L4 neurons are known to be primarily locally projecting neurons and to not send axons through the CC (Lübke and Feldmeyer. Brain Struct Funct. 2007 Jul;212(1):3-17; Schubert et al., Brain Struct Funct. 2007. 212, 107-119). This is clearly confirmed by GFP visualization of Rorb axons in the P30 circuit. We quantified axons that enter the white matter without bias, i.e. not assuming that they extend contralaterally. However, we believe that these data clearly demonstrate an important developmental axonal remodeling that has not been reported before and which is coherent with our CTB results. Overall, these data contribute to demonstrate that S1L4 neurons become locally projecting neurons only after experimenting a transitory stage in which they project callosally.

In regard to the comment *"Similarly, for the panels showing dynamics of contralateral innervation comparing L2/3 and L4 of S1 (Fig 3A-B) the exposure/brightness contrast is clearly different for these panels".*

We have now included quantifications in new Figure 3c, described in Result section (pg 6) as suggested by the reviewer, and we have now slightly increased the brightness of L4 axons in the

panels of Fig 3b to demonstrate that we are not trying to hide any axons and to avoid confusion. These images were taken in equivalent conditions for quantification purposes, but at these stages S1L4 neurons lose their contralateral branches and they are more difficult to detect. We thank the reviewer for the suggestion of including quantifications. Nevertheless, we would like to clarify that the point of Figure 3a-b is to show the transitory projections of S1L4 neurons and are therefore somewhat confused by this objection. It would be difficult to manipulate images possessing the clearly detectable projections of S1L4 that we observe at P5 or P10, or to minimize the nearly non-existent (as expected) GFP signal at P30. As the reviewer points out, this is part of the novelty of our report.

To address point 3, the authors need to entertain the possibility that an alternative explanation for the main findings of axonal exuberance and pruning is that the number of neurons remain stable (or have altered dynamics to those shown) and that the composition of the surrounding cortical plate is changing, as is well known to occur throughout these periods. This problem arises because all of the quantifications are expressed as a percentage of DAPI positive nuclei, which does not reflect a stable number or population throughout development. It is therefore unsurprising that the percentage of CTB labelled neurons decreases, as the other later-born populations of the cortical plate (eg glia) increase in number. A complete switch to (or addition of) data presented as absolute cell numbers is critical to conclusively demonstrate the results reported, and without this, the manuscript's entire conclusions would need to be altered to reflect the uncertainty of this effect.

We have corrected this flaw in this new version and have also performed two important sets of experiments to address this concern. The reviewer is correct that we failed to explain the potential contribution of glial cells to our quantifications. These new experiments validate the refinement rates obtained from quantifications using DAPI and definitively discard potential dilution effects due to non-neuronal expansion in our experiments.

At the initiation of this project we considered the possibility that the decreases in the numbers of callosal neurons that we detected over the course of development were affected by the dilution of a fixed number of neurons by expansion of other cell types, such as glia, but ultimately discarded this explanation as extremely unlikely for reasons that are discussed below. In this revised version, and since this possibility cannot be fully discarded without testing, we also addressed the question experimentally.

We have performed two sets of experiments, which we believe completely and definitively discard artifacts due to non-neuronal expansion. Both validate our initial quantifications of CTB+ as a percentage over DAPI, and very importantly, reproduce the refinement rates we observed (see below).

Our initial reason to discard glial contribution to the reductions in the percentages of CTB-labeled neurons was that published reports indicate that glial expansion in the neuronal laminas has a low impact on changes in cell numbers within the period of P10-P30. In the cortical plate, most

glial cells are located in deep layers and their maximum expansion in the P1-P30 period is between P0 and P7 (Qiu et al., Neurosci Bull. 2007Jan;23(1):46-52; Fu et al., Brain Struct Funct. 2013 Sep;218(5):1337-54; Tabata. Front Neurosci. 2015 Apr 7;9:114; Ge et al., Nature. 2012 Mar 28;484(7394):376-80; and Bandeira et al., Proc Natl Acad Sci U S A. 2009 Aug 18;106(33):14108-13; Keller et al., Front Neuroanat. 2018 Oct 23;12:83). We failed to clarify that, in order to allow proper cortical lamination and to avoid potential confounding effects from early proliferation, early cell death, and incomplete neuronal migration of pyramidal and interneuron populations, all our CTB quantifications (including injections performed at P3) were performed in brains of mice no younger than P10, and no older than P32. We apologize that we failed to describe this in sufficient detail. We have now included a longer description of these considerations and the details of our quantifications in the main text, Methods section "Confocal Imaging and quantifications" (pg 15-16).

We would like to clarify that in the cortex, as the reviewer indicates, although glial proliferation proceeds during postnatal development from around P5 to 3 months resulting in huge increases in cell numbers, this proliferation occurs at different stages and locations in the cortex. There are various studies describing glial proliferation and colonization in the cortex. According to these studies, glial proliferation can be roughly subdivided into two periods: an initial one from P5-P30, and a second one that commences at around P30 which also contributes to significant growth.

The P5-P30 period (which does pertain to our quantifications) is the initial phase of glial expansion. It begins at around P3-P5 with the initiation of an intensive proliferation of subventricular precursors. These give rise to cells that primarily populate layer 1 and, more abundantly, the corpus callosum (mostly oligodendrocyte precursors and oligodendrocytes). The addition of these new cells is the main contribution to cell expansion at these stages, and nearly doubles the total number of cortical cells (Ge et al., Nature. 2012 Mar 28;484(7394):376-80; and Tabata. Front Neurosci. 2015 Apr 7;9:114) .

In the neuronal layers, the number of non-neuronal cells in the mature brain is highest in the deep layers of the cortical plate and much lower in the upper layers (estimated numbers are maximum 1:1 in deep layers (Keller et al., Front Neuroanat. 2018 Oct 23;12:83). The precursors of these glial cells appear in the cortex early postnatally. It is reported that during the early phases of postnatal development there is *in situ* proliferation of the glial precursors that is particularly significant between P5 and P7 in deeper layers. After that, it appears that within the period of P7-30, there is reduced proliferation that contributes to very small increases in total cell numbers in the neuronal laminas (Ge et al., Nature. 2012 Mar 28;484(7394):376-80; and Tabata. Front Neurosci. 2015 Apr 7;9:114; Qiu et al., Neurosci Bull. 2007Jan;23(1):46-52). Hence, because we quantified our early CTB injections at P10 or later (now clarified in Methods, pg 15), we see no major effects caused by glial proliferation. This is confirmed by the new experiments and quantifications we performed (see below).

The second very important reason why we discarded any serious dilution effects caused by non-neuronal populations is the extreme refinement rates that we observe. As an example, in S1L4 we observed refinement rates of greater than 90% from P3 to P30. If this reduction in callosal neurons were due to an increase in glial cells it would require the generation of more than 90 glial cells per callosal neuron. This hypothetical number would be in stark contrast to the reported numbers of glial cells in neuronal layers (not greater than 1:1 neuron:non-neuronal), and in particular the very low numbers of glia in L4, (Bandeira et al., Proc Natl Acad Sci U S A. 2009 Aug 18;106(33):14108-13; Keller et al., Front Neuroanat. 2018 Oct 23;12:83).

We performed the following experiments to validate our methods of quantification and to discard artifacts or over-estimation of refinement due to increases in the total cell numbers of the cortex:

The first and, in our opinion definitive, experiment consisted of double injections of CTB tagged with distinct colors at P5 and P30 in the same animal, and subsequent analysis of the labeled cells at P32 (new Supplementary Figure 2d-f), mention in Results section (pg 5), Methods, “Confocal imaging and quantification” (pg 16), and in Supplementary Text “CTB injections” (pg 1,2). In this experiment, the number of cells (neurons) labeled at P5 was used in the denominator rather than DAPI in order to calculate the refinement at P30 (double-labeled neurons). This is important because the experimental design precludes any possible influence from glial expansion. Thus, the refinement that we detect in these double-injected brains is necessarily chiefly neuronal. In addition, by using a neuronal population in the denominator as an internal control, we eliminate the potential to skew cell counts due to imprecise delimitations of different cortical areas at different development stages as may be the case when performing measurements of absolute numbers (also performed for the barrels, see below). It also has the advantage that populations labeled at P5 and at P30 are quantified at the same stage in the same animal. New Supplementary Figure 2f shows the comparison of the refinement rates obtained with this method and the rates obtained by counting CTB+ over DAPI-labeled cells. There are no statistically significant differences between these two methods (or with absolute counts as described below). There are only some minor differences in the mean values and in the precision (variability among the values obtained at the same stage for each method). Counting of double-labeled cells in the same animal is by comparison extremely precise. Nevertheless, using both methods, the variability is low and counting over DAPI has the advantage that it diminishes the number of procedures per animal and it allows comparison between conditions at the same stage. The results from this experiment definitively discard potential artifacts or dilution effects due to glial proliferation or the migration of interneurons, and it confirms the utility of calculating the refinement rates using DAPI+ nuclei. The results of the comparison between quantifications are in accordance with the literature’s description of the dynamics of glial expansion in neuronal layers. They also discard any meaningful contribution from CPN death to refinement because we obtain indistinguishable values of refinement using the three quantification methods described above. This is now mentioned in the Results section (pg 5) and in the Method section (pg 16), and in Supplementary Text “CTB injections” (pg 1,2).

The second approach we used to confirm and validate our quantification technique was, as suggested by the reviewer, quantifications of absolute numbers. The results are shown in new Supplementary Figure 3 and mentioned in Methods section (pg 16). We have counted the total number of CTB^+ cells in the S1 region as delimited by the clear anatomical landmarks of this area (i.e the barrels). These quantifications at different developmental stages revealed refinement rates that were indistinguishable from those obtained by counting over DAPI, and also indistinguishable from quantifications in double injected mice (data shown in new Supplementary Figure 3j-n and Supplementary Figure 2f). We therefore feel confident that we can discard potential dilution effects due to non neuronal expansion in our experiments.

We would like to mention that, unfortunately, we do not have access to any automated sterology system for cell counting in the brain. Also, we found some disadvantages of absolute cell counts, which perhaps are more often performed in studies comparing different conditions in adult animals, and less often used when comparing developmental stages. When describing changes in specific areas, as opposed to measurements of total numbers of cells in the brain, one of the disadvantages of absolute cell counts is the necessity to very precisely define the area of interest at all stages of development (Keller et al., Front Neuroanat. 2018 Oct 23;12:83). Errors in delimiting areas are less pronounced in S1 because of the barrel field structures, but delimiting other regions, such as S2 or M1, or even the visual cortex, where specific cytoarchitectures and landmarks only develop at later stages, may produce more error in the measurements. Our quantifications over DAPI cells in cortical columns have the advantage of limiting variability in defining equivalent developmental areas (there is a chance of error when selecting the precise column within an area, but less than when having to delimit the total developmental area with precision).

Hence, although the main conclusions are not altered, we acknowledge that the non-neuronal population is a relevant one to consider when performing these experiments, particularly in the deep layers, and we apologize for the confusion our previous version may have caused. We have corrected all the graphs to clearly reflect that the presented values are the % of CTB+ neurons over DAPI. We have also explained in detail in Methods how quantifications were made (pg 15-16) and that quantifications were performed at P10 or later (pg 15-16) to facilitate comparison between stages. We have also discussed the comparisons between the different quantification methodologies in Methods (pg 15-16).

In summary, in response to the most important concern of the reviewer, all quantifications confirm the refinement rates we observe using DAPI, and we have made clarifications describing the methodology (new Supplementary Figures 2e, f and 3 and Methods sections (pg 15-16) of the revised version). The new quantifications are mentioned in the Results section (pg 5, 6).

We thank the reviewer for this comment and agree that these validations significantly strengthen our conclusions and are an important improvement in the manuscript. They have also laid the groundwork for future experiments in our group and in others. Injecting CTB and counting labeled cells over DAPI stained nuclei is a highly accessible methodology that facilitates comparisons between different developmental stages, and we now demonstrate that it gives accurate data

when analyzing brains between P10-P30. We hope these analyses will lead to fruitful advances in the field and a broad use of these injection approaches.

Additional specific comments below are predicated upon the assumption that a switch in all parts to a quantification of absolute cell number would reveal the same patterns as those reported by percentage:

• Interpretation: Throughout the introduction, results and discussion, there are repeated iterations of the statement (or variations thereof) that "all cortical neurons project callosally initially/ by default". The results of the paper themselves show that this is not true – e.g. L6 neurons of S1 never exhibit more than 60% of interhemispherically-projecting neurons out of all DAPI-positive nuclei. These conclusions should be modified to reflect the true nature of the findings, that select populations of all cortical layers (of unknown molecular identity) have the potential to project callosally initially.

We agree with the reviewer and have modified all of these sentences in order to more accurately reflect the data.

• Many of the most striking and novel results presented by this paper are not the ones actually quantified and/or statistically analysed. For instance, nowhere is there a quantification of the absolute number of retrogradely labelled cells from the callosal tract in any cortical area throughout development, with a statistical comparison over time.

The closest to this is Fig 1k-o, which compares areas (not ages), and which quantifies %CTB neurons. This quantification is not appropriate to show that there is an exuberance and refinement of callosally-projecting neurons, as it relies on a stability of cortical composition that simply isn't there. E.g., there is increased production of glia and interneurons throughout development in the cortex that could mean a stable absolute number of callosally-projecting cells would appear to decrease as a percentage of total number, simply because of a progressive increase in other cell types. The qualitative images seem like this is not the case, but an appropriate quantification and statistical analysis comparing absolute number of contralaterally-projecting neurons throughout development is absolutely critical to conclusively demonstrate the results purported. This change to absolute number of cells would also increase the validity of subsequent analyses throughout the paper (e.g. sensory deprivation/thalamic manipulation etc), as they face the same problem of potentially manipulating cortical composition rather than refinement of callosal populations, as concluded by the authors.

As described above, we have now counted the absolute numbers of cells in all layers of all stages for S1 (new Supplementary Figure 3). We have limited this type of quantification to this area due to the difficulties in delineating other cortical areas with similar precision. We have also now validated the use of DAPI to quantify CTB labeled neurons by comparing these values to quantifications of absolute numbers and by comparing to refinement rates obtained from double

injected animals. Importantly, we see the same refinement rates using all three quantifications (Supplementary Figure 2f). The new version also now provides appropriate statistical comparisons. We believe these validations strengthen our work and thank the reviewer for this very useful criticism.

A figure/quantification showing the changing distribution of labelled cells as proportions per layer throughout development would also be welcome.

We have now included a figure representing the changing distribution of labeled cells for S1, the region for which we counted absolute numbers of CTB+ cells (new Supplementary Figure 3i). These representations are useful to show the contribution of each layer to the total interhemispheric connectivity at each stage. However, they do not to represent the process of refinement as clearly as the representation of the proportion of CTB cells per layer (Figure 1 k-o). For example, since deep layers refine much less than L2-3 and L4 after P5, their proportional contribution to the total interhemispheric connectivity increases during postnatal maturation correlating with the refinement of upper layers (new Supplementary Figure 3i).

• *Figure 2e-i shows qualitative descriptions of a striking decrease in contribution of L4 neurons to the callosal tract throughout development. It is unclear why the authors have not quantified this phenomena, instead quantifying (in a different set of ages) L4 neurons with axons entering the white matter (which could conceivably be projecting ipsilaterally; Fig 2 k,l). A rigorous quantification of this phenomena, showing supplementary insets of the electroporated cells at each age (such that cell body distribution can be assessed) and with appropriate normalisation, would greatly enhance the impact of this figure and the interpretation of the entire manuscript.*

We have now included these quantifications and the requested insets of the ipsilaterally electroporated cells (Fig 2j and Supplementary Figures 4a,b).

As described above, we agree the axons quantified in Figure 2k-m may not be callosal, but we wanted to highlight the remodeling of these projections and to describe it in as great detail as possible. We present these panels and quantifications to illustrate that during development they do send axons to the WM but that these axons are transitory, and with the intention of demonstrating an important developmental axonal remodeling which is coherent with our CTB results and that has not been previously described. Overall, these data contribute to demonstrate that S1L4 neurons undergo significant remodeling and become locally projecting neurons only after experiencing a transitory stage in which they project callosally.

Cell counts of the number of electroporated cells (absolute numbers, not percentages) would also help to interpret the issue of cell death raised on page 5 and go towards evidencing the statement that "These transient S1L4 callosal axons likely retract or disassemble, as we did not observe broken GFP axonal fragments in the CC or cortical plate, or neuronal death, during their period of refinement".

We agree with the reviewer. In new Supplementary Figure 4a, b, we have included quantifications showing that in electroporated Rorb-Cre brains, the number of GFP^+ cells per barrel does not change with developmental stage. This is mentioned in the Results section (pg 6). The issue of cell death is also addressed by our double injections shown in new Supplementary Figure 2 as mentioned above.

• *Given that Fig. 4d-f aims to demonstrate that the refinement of contralateral projections from L4 S2 neurons is less than for L4 S1 neurons, it would be beneficial to quantify this effect and directly compare to L4 S1 quantifications throughout development (e.g. Fig 2e-i).*

We have now included these quantifications and comparisons (main Fig 4m and Supplementary Figure 5e-h).

• *In panels Fig1k-o, and again for Fig6h-l, the earliest age of injection is missing for one of the conditions. Upon cursory perusal of the figure, this may give an impression of differing dynamics between conditions that may not be the case. The reason for the absence of these datapoints should be clarified, and they should be incorporated if possible.*

We have now included an explanation for this in the Figure 6 legend. The reason for the absence of this data point is the impossibility of performing injections earlier than P7 in SertCRE;DTA mutant mice due to poor recovery after the injection surgeries. Mice appear to be more vulnerable at earlier stages, but at later stages are able to recover and feed properly.

• *On page 6, the text states that "The location of these contralateral columns is the same as SL2/3 callosal columns". It is unclear from this whether or not the authors have performed an electroporation in S1 or S2 (presumably at E15) to make this comparison.*
Further, the figures referred to in order to evidence this statement (Fig. 4h, i) do not show location at all in the context of medial-lateral position in the contralateral cortex, but rather demonstrate altered laminar distribution (again, unfortunately the proposed similarities/differences in location or laminar distribution are not demonstrated by quantification and statistical comparison).

We have clarified this in the text and added new Supplementary panels and additional statistical comparisons. In this revised version we clarify that, when comparing the locations of the S2L4 to SL2/3 columns, we refer to the columns formed by axons of the entire SL2/3 population at the S1/S2 border (Results pg 6) and included the new Supplementary Fig 5a showing this pattern. Our intention is to describe the positions of the previously unreported S2L4 axons that remain callosal and provide a comparison with the well-known pattern of innervation of a typically callosal population (L2/3). It is interesting to observe their similar patterns: they appear to target the same areas but different layers.
For SL2/3 we show columns observed in a brain electroporated in both S1 and S2, as both are reported to innervate the same contralateral area indistinguishably. These patterns have been

previously reported by us and others (Suárez et al. Neuron. 2014 Jun 18;82(6):1289-98; Rodriguez-Tornos et al., 2016 Neuron Feb 3;89(3):494-506). We have now included panels showing the pattern of innervation of GFP signal from SL2/3 neurons targeted after electroporating at E15 in Supplementary Fig 5a. The S1/S2 columns formed by contralateral axons from S2L4 magnified in Figure 4h are those appearing in Figure 4d and f. The complete SL2/3 image that we cropped for Figure 4h is now shown in the new Supplementary Figure 5a to facilitate comparison. We have also now performed the required statistical comparison to show differences in laminar distribution (shown in Fig 4i).

• *The representative images of CTB cells traced following bilateral cauterization (Fig. 5c and d) show a striking increase in labelled S1 L2/3 cells after bilateral cauterization at P30. However, this phenomena is not reflected in the quantifications, which reveal a decrease (Supplementary fig h).*

We think that there might be confusion here due to how we cropped the WT image used for the inset. We included in Supplementary Figure 8 (Supplementary Figure 6 in the original version) the full image showing the whole somatosensory area of interest. In these images, it can be observed that there is no reduction. However, as the reviewer can appreciate in this and in other figures, the density of CTB^+ cells is always lower in the area of L3 that borders L4. This difference in density is not as extreme in the bilaterally cauterized mice likely due to the lack of thalamic input. We have modified how this inset was cropped (Fig. 5c). It now shows a greater area of SL2/3 to more accurately reflect our results. We have also modified the text in the Results section to clarify this (pg 8). Also, we point out that in the original version the same quantifications that are now shown in new Supplementary Fig 8 showed that a significant decrease is observed only when unilateral cauterizations are performed. Quantifications of SL2/3 in bilaterally cauterized animals show no statistically significant changes, although the trend to a small reduction indicates that a small decrease cannot be discarded. This is agreement with published data (Suárez et al. Neuron. 2014 Jun 18;82(6):1289-98).

This is concerning because it is well known that the callosal tract is topographically arranged with respect to layers and mediolateral areas, such that a change in depth of injection could change the layer distribution and affect the results.

The spreading and extent of our injections and considerations about the importance of the depth of the injections are now fully described in the Supplementary Text (pg 2) and illustrated in new Supplementary Figure 1. We took these factors into account when performing our experiments. Illustrative images of brains at least two days after injections can be observed in Fig 1c-f and in the new Supplementary Fig 1b. We made sure that our injections properly filled the CC, included all axonal tracks of the CC, and did not spread to other regions this in now shown in new Supplementary Figure 1 e-h and mention in Supplementary Text (pg 2). As the reviewer has mentioned, the CC is topographically arranged. Indeed, we have observed during the optimization of our protocol that incorrect depth of injection leading to preferential filling of the

dorsal or ventral CC path gives rise to selective labeling of certain areas (such as more medial or lateral regions), but within the labeled area, the distribution of CTB labeled neurons is indistinguishable from our regular injections that completely fill the CC. Below, we include an image of an injection in the dorsal path. It can be observed that there is no labeling in lateral areas when we injected in the dorsal CC. Medial areas have the expected laminar distributions and somatosensory areas have poor labeling.

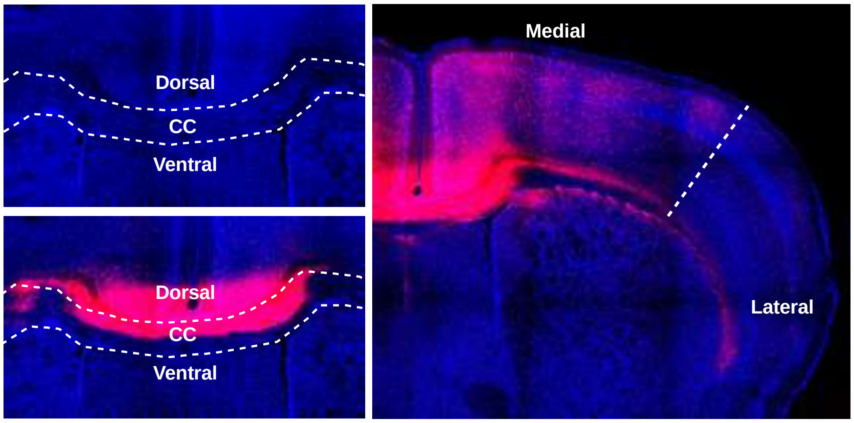

These inconsistencies highlight the need for clear quantification of absolute numbers of retrogradely labelled cells, as well as sufficient animal numbers and appropriate statistical tests to be certain of the effects described.

We hope that we have satisfactorily addressed the reviewer's concerns about our methods of quantification, and that our statistical analyses are now acceptable.

• *In Figure 5i, it would be of interest to statistically compare the dynamics of the Sert-Cre;DTA animals with the data collected from S2, as the authors are implying that this manipulation causes S1 to respecify to a more S2-like pattern of elimination. Also, are the absolute numbers of CTB positive neurons different between P5 and P30? – these should be compared within a group.*

We have now included a comparison of the dynamics S1 Sert-Cre;DTA and S2 in Fig 5j and clarify it in the Results section (pg 9). ANOVA analyses confirmed refinement between P5 and P30 (Fig 5i-j).

• *For supplementary figure 8, cell counts and/or normalised fluorescent analysis of the*

transcription factors described is needed to recognise a significant change between conditions, as the effect appears to be more subtle than would be expected with a normal range of variability in staining among control animals.

We have now included these quantifications. They are shown in the same figure, now named Supplementary Figure 10.

• *Text - Page 2: introduction "that facilitates the high functions of the human brain" Do the authors mean "that facilitates higher order functions of the human brain"? "capacity to emit interhemispheric projections" – wrong use of the term "emit"*

We have altered the text accordingly.

• *The English grammar needs to be checked throughout the manuscript*.
This has been checked by a native English speaker.

• *The title is not very informative and the abstract is quite confusing.* We have change the title to "Early callosal fate of most cortical neurons" and we have simplified the abstract.

It is recommended that the English grammar should be checked throughout.

A native English speaker has checked it.

Reviewers' comments:

Reviewer #1 (Remarks to the Author):

I appreciate the time and care, and considerable extra work, you have taken in addressing the review comments and the issues they raise. For my part most of the issues I discussed have been adequately dealt with in the light of the additional data presented and the changes to analysis and figures. I'd disagree that "myelination should equally affect all cortical areas" since the distribution of cortical myelin is quite heterogeneous (see any myelin stain of the mouse cortex) and this isn't necessarily reflected in the callosal axons anyway, since it is possible for axons to be myelinated along some stretches and unmyelinated in others. However given that 70% of callosal axons remain unmyelinated in the adult brain, this is probably not an issue.
I still feel that O'Leary's 1980s-90s work on exuberance and pruning of corticofugal axons should be at least acknowledged, to comment on your findings and suggest followup reading. Disregarding these important, closely related findings contributes to the fragmentation of our science and deprives the reader of important context. I'm sure you would not wish your work to be disregarded 30 years from now.

Matthew Kirkcaldie

Reviewer #2 (Remarks to the Author):

The authors have revised the manuscript providing more control experiments and a much better statistical analysis of their data. This is a very nice study and the authors have made some good progress in addressing the previous concerns.

However, as urged in the previous reviews, the claims of all/most cortical neurons project initially across the corpus callosum, has not been addressed and is still made continuously throughout the manuscript. The authors feel that this is their most significant finding, where in fact, their most significant finding is the results regarding layer 4 neurons specifically. This is the most compelling and novel data in the paper and is nicely verified with both CTB tracing and transgenic animals – these should be the focus of the paper. The authors statement that "This genetic axonal tracing is a highly reliable and independent confirmation of both the specificity and the efficiency of our CTB experiments" only replicates the data for L4 axons. It does not verify the data for all of the other layers.

In the previous reviews of this paper it was pointed out that papers from O'Leary and especially Innocenti (among others, of callosal projections, pruning and plasticity – still not referenced) had previously shown axonal pruning as a mechanism of cortical wiring. Therefore, these statements in the current discussion are not acceptable:
1) "We herein report a previously unrecognized wiring mode of cortical neurons that is based on an unexpected axonal plasticity and uncommitted callosal fates."
2) "These heretofore unreported transient axons"
3) "Our finding that there is broad axonal overshooting in the cortex is an important advancement in our understanding of the origin and functionality of the complex networks of the cortex."
4) "…other aspects of axonal development that have not been given sufficient attention previously"
Moreover, the statement that axons "overshoot and subsequently discard alternative networks during their selective wiring" is an over interpretation of their current data as no evidence was provided to show what other axonal projections (intracortical or descending projections) the neurons that lose their callosal projection have.

The authors could consider that the claim that "most cortical neurons" have an early callosal fate does not really make sense evolutionarily. Although all vertebrates have descending pathways from the

telencephalon, the analogous commissural structure across vertebrates is the anterior commissure, not the corpus callosum, which requires lateral projection. In order for most cortical neurons to be initially specified callosally would mean that mice have a completely different development than other mammals, such as marsupials and monotremes, which don't have a corpus callosum, and instead send their axons laterally and then medially across the corpus callosum.

Perhaps the authors think that cortical neurons project initially to all possible targets (not just the corpus callosum) and then prune some of them? This is different than projecting callosally, pruning, and then projecting to other targets. Since they provide no data on other possible targets it is not possible to know which of these models is correct.

It is very important that the authors cite and acknowledge previous literature showing axonal pruning as a mechanism of brain wiring specificity. Most of the references cited in the introduction are review articles, not the primary evidence and original journal articles that is required of a scholarly introduction to a primary paper. Reviews should not be cited in this context.

The authors are also urged to reduce their strong statements and over-interpretations with regards to the methods and to include at least supplementary figures 3 and 4 in their primary/main figures. These are great additions to the paper and go some way to addressing the methodological concerns of using CTB as previously pointed out (transneuronal labelling and labelling of fibres of passage), but these concerns will be front of mind for anyone that reads this paper and should be acknowledged and discussed in full in the manuscript.

Related to this, in the previous review it was pointed out to the authors that quantifications by percentage of total DAPI-positive cells were not a valid way to assess axonal pruning given that the number of DAPI-positive cells increases. The authors responded to this by saying that "We failed to clarify that, in order to allow proper cortical lamination and to avoid potential confounding effects from early proliferation, early cell death, and incomplete neuronal migration of pyramidal and interneuron populations, all our CTB quantifications (including injections performed at P3) were performed in brains of mice no younger than P10, and no older than P32. The revised methods reveal a misreporting in the original manuscript, which stated that "48 hours after all injections, mice were perfused with formalin and brains were fixed overnight". The authors now claim that animals were all collected at P10 for the P3, P5 and P7 injections, and 48 hours following surgeries for P14, P21 and P30. Apart from the fact that this is a large and worrying omission from the original manuscript, there is now the issue that the early ages of injection (P3, P5 and P7) have a much greater time between injection and collection than the older ages (P14, 21 and 30). This could result in increased numbers of neurons being labelled by CTB injection, and confound the results purporting to show refinement over development. Absolute numbers are now provided in the supplementary figures but this has done little to change the figures in the paper which still report the percentage of DAPI. Since they have found no difference why not report the actual numbers rather than the percentages?

Also related to the methods, the authors are encouraged to address the possibility that axons in the process of outgrowth/navigation (i.e. at earlier postnatal stages), may take up CTB more efficiently than axons that have already crossed (at late postnatal stages). This is yet another confound that may be misinterpreted as refinement over development.

All of these do not require further experiments but require the authors to modify their text substantially to downplay their claims that "All/most cortical neurons project callosally initially". Focusing on the L4 neurons would be the best way to 1) acknowledge previous literature showing populations of pyramidal neurons in most layers of the cortex have callosal projections and 2) that pruning of callosal axons occurs, 3) address and acknowledge that their promiscuous labelling might arise due to methodological issues, 4) highlight the most interesting and compelling evidence for this discovery of early L4 projections across the corpus callosum which are subsequently lost, 5) the L4 neurons would likely be most affected by sensory input and fits with their final data showing this.

There are also a few additional points which are now confusing:
Supplementary Fig.5 d – the GFP positive cells do not appear to co-label with CTB in the figure? In the figure legend it states "Coronal section from a Rorb-Cre mouse brain electroporated with floxed- GFP at E14 and injected with CTB in the CC at P30. Co-labeling of GFP and CTB confirms that L4 neurons projecting GFP+ callosal axons are those identified in our CTB labeling experiments."

There is something confusing about Fig.6, in the text it states, "Analysis of the developmental dynamics of callosal projections revealed that the differences in the number of CPN between Sert-Cre;DTA and WT arise from reduced refinement before P7 (Fig. 6h-l)." There are datapoints plotted for P5 and P7 and this statement appears to based on these two data points but in the figure legend it states, "No data from P3 or P5 Sert-Cre;DTA mice was collected because at these stages they recover poorly after the injection procedure." Is there a mistake in the figure? Was the same WT data used in both figure 1 and figure 6? Were the controls in figure 6 littermates of the Sert-Cre;DTA mice, or from a different strain entirely?

In figure S2e, it is unclear what has been quantified, as the y axis label says "%CTB colocalization, with the conditions of "555+/647+" and "647-/555+", presumably actually referring to the number or % of cells labelled in this pattern with the two fluorophores? This confusion is also a problem for S2b. Similarly, it is unclear how "% of refinement" was calculated in S2f, were the values obtained from P30 divided by the values obtained from P5? Was there any population of 647+/555- cells (it certainly looks as if there may be from the representative images in this figure)? Do the statistical differences between black and blue bars in S2e therefore only represent the proportion of single vs double labelling upon injection with 2 fluorophores? Clarification of precisely what these quantifications are remain necessary, as the authors purport that they are critical to addressing the major concerns of the initial review (and should perhaps therefore be in the main figures also).

Minor:
There is a cut and paste mistake in the legend for Fig.3 where "SL2/3 and S1L4 l axons " should be "S1L2/3 and S1L4 axons"
Legend for Fig. 4 "Two-Ways ANOVA" should be "Two-Way ANOVA"

MINISTERIO
DE CIENCIA, INNOVACIÓN Y
UNIVERSIDADES

CSIC
Consejo Superior de Investigaciones Científicas
Centro Nacional
de Biotecnología

Marta Nieto,
CNB, CSIC,
Darwin 3,
Campus de Cantoblanco,
Madrid 28049.
Tel 34 91 585 4837
Fax 34 91 585 4506
E-mail: mnlopez@cnb.csic.es

NCOMMS-18-31256 "Overshooting-wiring mode of cortical neurons". New tittle "Transient callosal projections of L4 neurons are eliminated for the acquisition of local connectivity"

Response to Reviewers' comments (Remarks to the Author from the reviewer are in bold italic):

Reviewer #1 (Remarks to the Author):

I appreciate the time and care, and considerable extra work, you have taken in addressing the review comments and the issues they raise. For my part most of the issues I discussed have been adequately dealt with in the light of the additional data presented and the changes to analysis and figures. I'd disagree that "myelination should equally affect all cortical areas" since the distribution of cortical myelin is quite heterogeneous (see any myelin stain of the mouse cortex) and this isn't necessarily reflected in the callosal axons anyway, since it is possible for axons to be myelinated along some stretches and unmyelinated in others. However given that 70% of callosal axons remain unmyelinated in the adult brain, this is probably not an issue.

I still feel that O'Leary's 1980s-90s work on exuberance and pruning of corticofugal axons should be at least acknowledged, to comment on your findings and suggest followup reading. Disregarding these important, closely related findings contributes to the fragmentation of our science and deprives the reader of important context. I'm sure you would not wish your work to be disregarded 30 years from now.

Matthew Kirkcaldie

Dear Dr. Kirkcaldie:

We sincerely thank you for your extensive review and the appreciation of our efforts. We believe that, in the process of responding to your comments, the manuscript has significantly improved. As for the remaining comments, we have made the following changes and additions in this new version:

We opted to delete the text in the Supplementary text section on CTB injections referring to possible differences due to myelin: “Second, we observe selective and specific distributions of callosal neurons in the different areas in the same animal at the same stage”.

Also, we would like to mention that in this version we have made important changes in writing in order to set the focus of the manuscript on our findings on layer 4. We have emphasized that results from CTB injections for layers other than L4 should be confirmed via independent methods as we can not fully evaluate all possible caveats, including the effects of myelin heterogeneity.

In regard to the acknowledgment of O’Leary’s work, we regret having given the impression that we disregard this seminal body of research in the field. We in fact frequently returned to it during our investigations. It is true that we relied too much on citing reviews, but we did so in the interest of brevity and in order to limit the number of citations. In the introduction and discussion of this revised version, we cite and discuss in greater depth these important early investigations.

We again thank you for the constructive process of this review.

Reviewer #2 (Remarks to the Author):

The authors have revised the manuscript providing more control experiments and a much better statistical analysis of their data. This is a very nice study and the authors have made some good progress in addressing the previous concerns.

We thank the reviewer for his appreciation of our revision and of the added experiments and figures. We also thank the reviewer for the detailed feedback. In this revised version we made the major change in the focus of the manuscript on our findings for L4 (including changing the title) requested by the reviewer and have made an extensive revision and rewriting, including presenting absolute data in main figures. We also address all the remaining concerns.

In brief, we consider that all of these main changes respond to the reviewer’s main concerns as summarized in the paragraph copied below:

“All of these do not require further experiments but require the authors to modify their text substantially to downplay their claims that “All/most cortical neurons project callosally initially”. Focusing on the L4 neurons would be the best way to 1) acknowledge previous literature showing populations of pyramidal neurons in most layers of the cortex have callosal projections and 2) that pruning of callosal axons occurs, 3) address and acknowledge that their promiscuous labelling might arise due to methodological issues, 4) highlight the most interesting and compelling evidence for this discovery of early L4 projections across the corpus callosum which are subsequently lost, 5) the L4 neurons would likely be most affected by sensory input and fits with their final data showing this”

Below there is a point by point response. We believe that all these changes appropriately respond to the reviewer's concern and have improved the manuscript. We thus would like to thank the reviewer for clarifying the main points that should be addressed.

Point by point responses:

However, as urged in the previous reviews, the claims of all/most cortical neurons project initially across the corpus callosum, has not been addressed and is still made continuously throughout the manuscript. The authors feel that this is their most significant finding, where in fact, their most significant finding is the results regarding layer 4 neurons specifically. This is the most compelling and novel data in the paper and is nicely verified with both CTB tracing and transgenic animals – these should be the focus of the paper. The authors statement that "This genetic axonal tracing is a highly reliable and independent confirmation of both the specificity and the efficiency of our CTB experiments" only replicates the data for L4 axons. It does not verify the data for all of the other layers.

We have made significant changes in the manuscript to address this point. These include changes in the title and in the text of the manuscript to focus on layer 4. For layers L2/3 and L5-6, we restrict ourselves to a more neutral description of the results of our CTB injections, in the hope that these experiments will aid future investigations. We thus have completely remodeled the introduction, results and discussion sections.

We have deleted the sentence "*This genetic axonal tracing is a highly reliable and independent confirmation of both the specificity and the efficiency of our CTB experiments".*

In the discussion section pg 11 of this new version we now state: "*In contrast, the use of Rorb-Cre mice allowed us to visualize the presence of L4 transient axons, thus providing a highly reliable and independent confirmation of the results we obtained for L4 populations in our CTB experiments*".

In the previous reviews of this paper it was pointed out that papers from O'Leary and especially Innocenti (among others, of callosal projections, pruning and plasticity – still not referenced) had previously shown axonal pruning as a mechanism of cortical wiring. Therefore, these statements in the current discussion are not acceptable:

1) "We herein report a previously unrecognized wiring mode of cortical neurons that is based on an unexpected axonal plasticity and uncommitted callosal fates."

2) "These heretofore unreported transient axons"

3) "Our finding that there is broad axonal overshooting in the cortex is an important advancement in our understanding of the origin and functionality of the complex networks of the cortex."

4) "…other aspects of axonal development that have not been given sufficient attention previously"

Moreover, the statement that axons "overshoot and subsequently discard alternative networks during their selective wiring" is an over interpretation of their current data as no evidence was provided to show what other axonal projections (intracortical or descending projections) the neurons that lose their callosal projection have.

We have deleted all these sentences. In addition, we now discuss extensively the *O'Leary and Innocenti* important early studies in the introduction and discussion.

The authors could consider that the claim that "most cortical neurons" have an early callosal fate does not really make sense evolutionarily. Although all vertebrates have descending pathways from the telencephalon, the analogous commissural structure across vertebrates is the anterior commissure, not the corpus callosum, which requires lateral projection. In order for most cortical neurons to be initially specified callosally would mean that mice have a completely different development than other mammals, such as marsupials and monotremes, which don't have a corpus callosum, and instead send their axons laterally and then medially across the corpus callosum.

We have deleted all claims about cortical neurons in general and about the early callosal fate of cortical neurons. We actually think that the most likely interpretation is the one given below by the reviewer, but as pointed out, we have no data to support one view or the other. We, therefore, have revised this new version as explained immediately below.

Perhaps the authors think that cortical neurons project initially to all possible targets (not just the corpus callosum) and then prune some of them? This is different than projecting callosally, pruning, and then projecting to other targets. Since they provide no data on other possible targets it is not possible to know which of these models is correct.

We suspect this is most likely what is occurring, but as the reviewer points out, we have not addressed this experimentally. We have added a small paragraph in the discussion section to briefly mention these points (pg 12). "*In this regard, our experiments did not assess whether L4 adult connectivity emerges from the selective elimination of callosal axons and the concomitant stabilization of other projections, or from the elimination of failed callosal connections followed by the growth of new projections to other targets*". We thank the reviewer for this constructive comment.

As stated above, in this revised version we focus principally on L4 neurons and we only refer and discuss their callosal and acallosal potential wiring and not to other layers.

It is very important that the authors cite and acknowledge previous literature showing axonal pruning as a mechanism of brain wiring specificity. Most of the references cited in the introduction are review articles, not the primary evidence and original journal articles that is required of a scholarly introduction to a primary paper. Reviews should not be cited in this context.

We thank the reviewer for this comment. We have corrected this, specifically in the Introduction and the Discussion.

The authors are also urged to reduce their strong statements and over-interpretations with regards to the methods and to include at least supplementary figures 3 and 4 in their primary/main figures. These are great additions to the paper and go some way to addressing the methodological concerns of using CTB as previously pointed out (transneuronal labelling and labelling of fibres of passage), but these concerns will be front of mind for anyone that reads this paper and should be acknowledged and discussed in full in the manuscript.

We have transformed former Supplementary Fig. 2 and 3 into the new **main Fig. 2** and pannels from former Supplementary Fig. 4 are now added in **main Fig. 3e-f**. We only left some panels in the new supplementary Fig. 2. We thank the reviewer for this suggestion.

We have discussed the issue of methodological concerns of our CTB injections in the Discussion (pg 12), and as explained above, we have rewritten all of our conclusions that refer only to interpretations of results from CTB injections and have focused most of our discussion on L4. The specific concern about transneuronal labeling is now mentioned in pg 6, result section "*However, we could not fully discard caveats in the methodology, such as, for example, possible transneuronal processing of CTB or differences in CTB uptake due to heterogeneity in myelination. Importantly, one of the most striking observations was evidence of transient callosal projections in the majority of L4 neurons"* In Discussion pg 11*, "However, it is possible that our injection methodology could result in some degree of promiscuous or transneuronal labeling, or neurons could take up CTB with higher efficiencies at younger stages or depending on the degree of axonal myelinization. These and other unforeseen methodological issues with our injections could affect the reported refinement rates. Thus, the CTB labeling and refinement dynamics we report for layers other than L4 should be confirmed by independent methods".* On Supplementary Text, we discuss the experiments we have performed to evaluate the specificity of our CTB experiments including the effects of transneuronal labeling.

We would like to clarify that controls of the specificity transneuronal labeling were mentioned in the previous versions in Supplementary Text and also in the specific response to reviewer #1, but that we now expand the explanations. We agree that, to a certain extension, transneuronal labeling is a possibility that we cannot fully discard due to the amounts of CTB that we inject and because we cannot evaluate all possible differences in CTB uptake at the differences stages. Therefore, we agree that it is best to mention and discuss these issues. We hope that the reviewer agrees with these changes.

In regard to concerns about "labeling of fibres of passage", we respectfully would like to point out that we do not fully understand this point, but interpret the comment as a concern about labeling of "fibres of passage" as opposed to the terminal axon. In fact, we are actually purposely labeling callosal axons that could be considered *fibres on passage*. Since we label them in the contralateral hemisphere, this ensures that we label somas of neurons with a callosal projection.

When discussing the caveats of CC injections, we have opted to not mention this term specifically as in all cases this possibility is included in our referring to caveats in general in the Discussion section (pg 11).

Related to this, in the previous review it was pointed out to the authors that quantifications by percentage of total DAPI-positive cells were not a valid way to assess axonal pruning given that the number of DAPI-positive cells increases. The authors responded to this by saying that "We failed to clarify that, in order to allow proper cortical lamination and to avoid potential confounding effects from early proliferation, early cell death, and incomplete neuronal migration of pyramidal and interneuron populations, all our CTB quantifications (including injections performed at P3) were performed in brains of mice no younger than P10, and no older than P32. The revised methods reveal a misreporting in the original manuscript, which stated that "48 hours after all injections, mice were perfused with formalin and brains were fixed overnight". The authors now claim that animals were all collected at P10 for the P3, P5 and P7 injections, and 48 hours following surgeries for P14,P21 and P30. Apart from the fact that this is a large and worrying omission from the original manuscript, there is now the issue that the early ages of injection (P3, P5 and P7) have a much greater time between injection and collection than the older ages (P14, 21 and 30). This could result in increased numbers of neurons being labelled by CTB injection, and confound the results purporting to show refinement over development. Absolute numbers are now provided in the supplementary figures but this has done little to change the figures in the paper which still report the percentage of DAPI. Since they have found no difference why not report the actual numbers rather than the percentages?

We again would like to reassure that this omission was not intentional. We would like to point out that in addition to correcting the description of the methods and explaining the elections of the stages at which quantifications were made, the response to the original concern about quantifying neurons as a percentage of DAPI included experiments in which we compared these quantifications with absolute counts, and also with double injections. They demonstrated the validity and accuracy of the DAPI-based quantifications to faithfully reflect refinement rates. We have now changed these countings and comparisons to the main figure as requested.

As for concerns about how the number of days between injections might affect quantifications, they are best addressed by injections performed at P1, P3 and P5 that were shown also in the previous version. Injections at P1 and P3 labeled fewer cells than injections at P5, despite the latter having shorter periods of exposure to CTB. This is now discussed in Supplementary Text. These injections demonstrate that longer exposure to CTB does not result in increased labeling. These results were shown in the former Supplementary Fig. 2 and are now shown in the main Fig. 2o-p, and Supplementary Fig. 1c, 2d-f.

We have also added two new control experiments in Supplementary Figure 2a-b. Supplementary Figure 2a-b show control experiments of absolute numbers of CTB cells after injections performed at P5 and analyzed 5 or 27 days later. It shows that values were indistinguishable

(Supplementary Fig. 2a). We also performed experiments in which CTB injections were performed at P30 and analyzed 2 days or 7 days later, which also resulted in undistinguishable values (Supplementary Fig. 2b). We now expanded these explanations in Supplementary text.

We would also like to point out that, if there were increased labeling due to increased exposure to CTB, this would be reflected by both quantifications using DAPI and counts of absolute numbers of cells.

In regard to the figures expressing absolute numbers, as stated above, we have now included the quantifications and controls of absolute numbers previously shown in Supplementary Fig. 2 and 3 in the new **main Fig. 2**. Accordingly, we now describe these quantifications and control, and the validity of all three methods (percentage of CTB^+ over DAPI, absolute counts, and double injections) to analyze refinement rates in the Results section (pg 4-5). Here, we also indicate the importance of performing these controls and the advantages and disadvantages of the three types of quantifications.

In regard to the presentation of absolute numbers for the rest of the figures, we interpret that the reviewer agrees, once we experimentally proved that all methods are equally valid for defining refinement rates (and that this is now shown in main **Fig. 2q)**, that it is acceptable to show data as percentages of total cells instead of absolute counts in the rest of the figures. We would like to stress that this decision is not intended to bias any interpretation, but motivated by the following reasons: 1. All quantifications show identical refinement rates as demonstrated in S1. 2. We cannot identify appropriate anatomical landmarks to precisely define specific visual and auditory regions throughout development as can be done for the somatosensory region using the barrel fields. Thus, quantifications would have many more errors. 3. We believe that the most appropriate way to compare and communicate differences in refinement dynamics for different areas and layers is by using the proportion of callosal neurons, even when expressed as a percentage of DAPI and not relative to neurons. Percentages best reflect that initially all regions possess similar proportions of callosals and allow the comparison of changes over the course of development. If we, for example, had represented the absolute numbers of callosals of S1 and S2, (Fig. 5m), we would have encountered the issue that S2 is a much smaller region than S1. Thus, the initial and subsequent absolute numbers would be much smaller and the similarities that we observe by percentage would be masked, poorly evaluated and difficult to communicate. For similar reasons, other studies of the developmental dynamics of layer specific neuronal populations report the changes as a percentage of DAPI cells (Harb K, et al., Elife. 2016 Jan 27;5:e09531; Alfano et al., Nat Commun. 2014 Dec5;5:5632), 3. Very importantly, quantifications of absolute numbers of all data would require at least several months and produce no significant advances in the main results. 4. Either method of quantification would be equally affected by the possible caveats regarding injections.

In summary, we hope that the reviewer agrees that all the changes in the manuscript now properly address the issue of quantifications.

Also related to the methods, the authors are encouraged to address the possibility that axons in the process of outgrowth/navigation (i.e. at earlier postnatal stages), may take up CTB more efficiently than axons that have already crossed (at late postnatal stages). This is yet another confound that may be misinterpreted as refinement over development.

These issues were more extensively addressed in the response to reviewer #1. We have now addressed this in the discussion. "However, these do not fully discard some promiscuous labeling or a certain degree of transneuronal labeling, nor it evaluates the possibility that neurons uptake CTB with higher efficiencies at younger stages or how labeling is affected by the myelinization stage of the axons. This and other unforeseen methodological issues could affect the reporting of transient CPN by our injections. Thus, the CTB labeling and refinement dynamics we report for layers other than L4 should be confirmed by independent methods".

We would also like to reiterate that we had performed experiments that evaluate the saturating conditions of the injections to the best of possibilities. Also, that if we interpret this point correctly, this potential difference in the efficiency of CTB uptake would be reflected in low efficiency and non-saturating labeling of mature callosals and therefore, that the concern is that our injections in the adult and late postnatal mouse would possibly underestimated the number of callosal neurons. As elaborated in the previous revision, the adult patterns that we find are in agreement with previous reports and with the known interhemispheric connectivity in the adult. For clarity, we have changed how this is expressed in the manuscript. Results, pg 4: "*CTB injections in the CC of postnatal day (P) 30 animals and analysis of the opposite hemisphere 48h later revealed broad labeling of different cortical areas (Supplementary Fig. 1a- b). It revealed area- and layer-specific CPN distributions (Fig. 1d, f, h, j) in agreement with previous reports (Fame et al., 2011), including very few CPN in L4 (Fig. 1h, j)".*

Or perhaps the concern refers to higher transneuronal labeling in young animals, which was addressed as discussed above. In any case, as the reviewer points out, all of these concerns are best resolved by focusing on L4, as this data is confirmed by the visualization of projections.

All of these do not require further experiments but require the authors to modify their text substantially to downplay their claims that "All/most cortical neurons project callosally initially". Focusing on the L4 neurons would be the best way to 1) acknowledge previous literature showing populations of pyramidal neurons in most layers of the cortex have callosal projections and 2) that pruning of callosal axons occurs, 3) address and acknowledge that their promiscuous labelling might arise due to methodological issues, 4) highlight the most interesting and compelling evidence for this discovery of early L4 projections across the corpus callosum which are subsequently lost, 5) the L4 neurons would likely be most affected by sensory input and fits with their final data showing this.

We thank the reviewer for taking the time to summarize these points. As stated earlier, the reviewer can observe that we have modified the manuscript substantially and included all of these changes in the new version.

There are also a few additional points which are now confusing:

Supplementary Fig.5 d – the GFP positive cells do not appear to co-label with CTB in the figure? In the figure legend it states "Coronal section from a Rorb-Cre mouse brain electroporated with floxed- GFP at E14 and injected with CTB in the CC at P30. Co-labeling of GFP and CTB confirms that L4 neurons projecting GFP+ callosal axons are those identified in our CTB labeling experiments."

These are the low magnifications that correspond to the panels shown on the main Fig. 5j (former Fig. 4j). This was indicated in the figure legend of this figure. Now changed in figure legend Supplementary Fig.4 d: "Coronal section from a Rorb-Cre mouse brain electroporated with floxed-GFP at E14 and injected with CTB in the CC at P30 corresponding to the insets shown in main Fig. 5j". We apologize because we realize that as it was formerly expressed, it appears as if colocalization should be observed in S1 and S2. In this revised version, we have included in the Supplementary Fig. 4d, the panel showing high the magnification of S2 that are also shown in main Fig. 5j. We specified in the figure legend: "*co-labeling of GFP and CTB in S2 confirms that L4 neurons projecting GFP+ callosal axons are those identified in our CTB labeling experiments*".

There is something confusing about Fig.6, in the text it states, "Analysis of the developmental dynamics of callosal projections revealed that the differences in the number of CPN between Sert-Cre;DTA and WT arise from reduced refinement before P7 (Fig. 6h-l)." There are datapoints plotted for P5 and P7 and this statement appears to based on these two data points but in the figure legend it states, "No data from P3 or P5 Sert-Cre;DTA mice was collected because at these stages they recover poorly after the injection procedure." Is there a mistake in the figure? Was the same WT data used in both figure 1 and figure 6? Were the controls in figure 6 littermates of the Sert-Cre;DTA mice, or from a different strain entirely?

This issue was raised in the first review, at which point we added: "*No data from P3 or P5 Sert-Cre;DTA mice was collected because at these stages they recover poorly after the injection procedure*." In an attempt to further clarify the issue, we changed to sentence to: "*No data from P3 or P5 Sert-Cre;DTA mice was collected because at these stages* ***mutants*** *recover poorly after the injection procedure*." We have also altered the text to state: *"Analysis of the developmental dynamics of callosal projections indicated that the differences between Sert-Cre;DTA and WT originate from early in development, as the numbers of CPN were higher in mutants at all comparable stages of the analysis (Fig. 7h-l)."*

In Figures, dashed lines indicated the comparison with data described in previous figures. Data in former Fig. 6, now Fig. 7 includes data of WT from main Fig.1. We have added this clarification in Figure legends. Data from controls is now shown in new Supplementary Fig. 10.

WT and mutants are not from different strains. As stated in material and methods, WT and Sert-Cre;DTA mice were obtained from the same crosses and colonies and experimental groups might include littermate pairs, but in the majority of experiments mice are not littermates. This is now better reflected in Methods (pg 15): *"Slc6a4tm1(cre)Xz/J (Sert-Cre) mice (Jax stock #014554 and #009669), and R26:lacZbpA(flox)DTA (DTA) heterozygous mice {Brockschnieder, 2006 #127}*

were maintained in heterozygosis in the C57BL/6JRccHsd background (Envigo laboratories, formerly Harlan). Sert-CRE;DTA mutants were obtained by crossing heterozygous mice. WT animals were obtained from these colonies". For electrophysiology experiments shown in Figure 7, we did use two pairs of littermates out of 6 mice and this is now reflected in the figure legend.

In figure S2e, it is unclear what has been quantified, as the y axis label says "%CTB colocalization, with the conditions of "555+/647+" and "647-/555+", presumably actually referring to the number or % of cells labelled in this pattern with the two fluorophores? This confusion is also a problem for S2b.

As the reviewer states, this refers to the % of cells labeled with the two fluorophores. Former Fig. 2e is now split into main Fig. 2o-p (to show refinement) and Supplementary Fig 2e-f. Examples of cortical columns of double injected mice are shown in both. For clarification, the x axis of Fig. 2p is now labelled: % 647^{-}/ Total 555^{+}. In this version Supplementary Fig. 2c and 2f: in the x axis: % cells, legends indicate: $555^{+}/647^{+}$ and $647^{-/}555^{+}$.

Similarly, it is unclear how " % of refinement" was calculated in S2f, were the values obtained from P30 divided by the values obtained from P5?

Yes, the y axis values (% CTB localization) representing % of refinement were obtained by dividing values at P30 by the values obtained from P5 injections. We have changed the figure legend. In this version Supplementary Fig. 2f is now main 2q. In former Supplementary Fig. 3: "Values are normalized to the percentage of the mean values obtained in P5 mice for each quantification method. Now in legend of Fig. 2: "For comparison, values are expressed as a percentage of CTB colocalization which represents refinement of P5 CTB^{+} cells". We have also added an additional diagram in Fig. 2o (right-most panel) to illustrate how we identify the cells that have refined callosal projections. This figure serves to emphasize the cells in layer 4 that are single-labeled and thus refined.

Was there any population of 647+/555- cells (it certainly looks as if there may be from the representative images in this figure)?

There are very few single 647+; 555- cells as observed in the graph shown in Supplementary Fig. 2f (former Supplementary Fig. 2b and 2e) (Their proportions can be extracted from the figure: 100- 555+/647+). These quantifications serve as a control for saturation of our injections, as explained in the Methods and Supplementary Text.

As for the representative images of double injections, there was a problem with the conversion of colors (RGB systems) when the PDF was created from the Illustrator file. We have corrected this. Cortical columns of double injected mice showing both single and double fluorescence images are now shown in main Fig. 2o and Supplementary Fig. 2c and 2e. Because the merging of two colors in these images not always results in clear blending of colors, we now show high magnifications of cells in Supplementary Fig. 2e.

Do the statistical differences between black and blue bars in S2e therefore only represent the proportion of single vs double labelling upon injection with 2 fluorophores?

Yes, the statistical differences refer to the proportion of single and double labeled cells. These differences in double labeling among layers indicate that there is specific refinement and do not support just a random distribution of the CTB for each probe, in which case the distributions should be indistinguishable. This is shown in Supplementary Fig 2e, as before. For clarity, the statistical differences in refinement rates among the different layers are shown in main Fig. 2p explained above and below.

Clarification of precisely what these quantifications are remain necessary, as the authors purport that they are critical to addressing the major concerns of the initial review (and should perhaps therefore be in the main figures also).

In this revised version we have moved this analysis of refinement to the main figures, and for clarity we now separate the graphs into two figures. Fig. 2p represents refinement rates obtained from double injected animals and shows the statistical difference of L4 compared to all other layers. The panels demonstrating the differences between 555+/647+" and "647-/555+" are in Supplementary Fig. 2e. We have also clarified how refinement rates were obtained in double injected animals in the Results section (pg 5): "*We also assessed the possible contributions of cell death to refinement rates. To do so, we performed dual sequential injections of CTB coupled to distinct fluorescent probes in the same animal at P5 and P30. In these S1 double-injected animals, we counted the fraction of cells labeled with the first fluorophore and not with the second, thus reflecting the elimination of neurons that had an early callosal projection and survive in the mature circuit (Fig. 2o-p and Supplementary Fig. 2c-f). As with the single injections, the highest refinement rates were observed in S1L4. Further, these double injections indicated that most neurons that comprise L4 in P30 animals have eliminated a P5 callosal projection (Fig. 2o-p)*".

Minor:

There is a cut and paste mistake in the legend for Fig.3 where "SL2/3 and S1L4 l axons

" should be "S1L2/3 and S1L4 axons"

Legend for Fig. 4 "Two-Ways ANOVA" should be "Two-Way ANOVA".

These have been corrected. We thank again the reviewer for such a detailed revision and for the constructive review. We hope that the reviewer finds that the new version of the manuscript has addressed all these important concerns.

REVIEWERS' COMMENTS:

Reviewer #2 (Remarks to the Author):

The authors have done an excellent job of addressing the previous concerns and the paper is vastly improved.

It describes very interesting data on the reassignment of layer 4 neurons from having a callosal projection to local connectivity. Previously the Nakajima lab (Oishi et al, 2016 PNAS) showed that Ror beta inhibits Brn2

expression to delineate L4 from L2/3 neurons. In addition, the Hanashima lab's recent paper (Hou et al., 2019 Nat. Comm) also provides evidence that Foxg1 overexpression can transform L4 neurons into callosal projection neurons. The authors should cite this work as a possible mechanisms for the reassignment of L4 neurons from projecting callosally to becoming purely local circuit neurons.

Marta Nieto,
CNB, CSIC,
Darwin 3,
Campus de Cantoblanco,
Madrid 28049.
Tel 34 91 585 4837
Fax 34 91 585 4506
E-mail: mnlopez@cnb.csic.es

Jerome Staal, PhD
Associate Editor (Life Sciences)
Nature Communications

NCOMMS-18-31256-C "Transient callosal projections of L4 neurons are eliminated for the acquisition of local connectivity"

Madrid 6 th September 2019

Dear Jerome,

Thank you very much for revising our manuscript. We are very happy that the reviewer is satisfied with our revision. In this revision, we have included the last suggestions of the reviewer as detailed below.

Reviewer #2 Remarks to the Author:

The authors have done an excellent job of addressing the previous concerns and the paper is vastly improved. It describes very interesting data on the reassignment of layer 4 neurons from having a callosal projection to local connectivity. Previously the Nakajima lab (Oishi et al, 2016 PNAS) showed that Ror beta inhibits Brn2 expression to delineate L4 from L2/3 neurons. In addition, the Hanashima lab's recent paper (Hou et al., 2019 Nat. Comm) also provides evidence that Foxg1 overexpression can transform L4 neurons into callosal projection neurons. The authors should cite this work as a possible mechanisms for the reassignment of L4 neurons from projecting callosally to becoming purely local circuit neurons.

Response:

We thank the reviewer for his appreciation of our revision. In this new version we cite these work in the Discussion section. "Molecularly, transcription factors such as Brn1/2, which inhibits Rorb and several L4 characteristics in L2/3 (Oishi et al, 2016 PNAS), or Foxg1, required for interhemispheric connections (Hou

et al., 2019 Nat. Comm), might be involved in the maturation of L4 neurons as callosal or ipsilateral-only connected neurons in specific sensory circuits”.

Sincerely,

Marta Nieto. PhD
